# AvmM catalyses macrocyclization through dehydration/Michael-type addition in alchivemycin A biosynthesis

**DOI:** 10.1038/s41467-022-32088-4

**Published:** 2022-08-03

**Authors:** Hong Jie Zhu, Bo Zhang, Wanqing Wei, Shuang He Liu, Lang Xiang, Jiapeng Zhu, Rui Hua Jiao, Yasuhiro Igarashi, Ghader Bashiri, Yong Liang, Ren Xiang Tan, Hui Ming Ge

**Affiliations:** 1 State Key Laboratory of Pharmaceutical Biotechnology, Chemistry and Biomedicine Innovation Centre, Institute of Artificial Intelligence Biomedicine, School of Life Sciences, Nanjing University, Nanjing, 210023 China; 2 State Key Laboratory of Coordination Chemistry, Jiangsu Key Laboratory of Advanced Organic Materials, Chemistry and Biomedicine Innovation Centre, School of Chemistry and Chemical Engineering, Nanjing University, Nanjing, 210023 China; 3 State Key Laboratory Cultivation Base for TCM Quality and Efficacy, School of Medicine and Life Sciences, Nanjing University of Chinese Medicine, Nanjing, 210023 China; 4 Biotechnology Research Center and Department of Biotechnology, Toyama Prefectural University, Toyama, 939-0398 Japan; 5 Laboratory of Molecular and Microbial Biochemistry, School of Biological Sciences, The University of Auckland, Auckland, 1010 New Zealand

**Keywords:** Enzyme mechanisms, X-ray crystallography, Biosynthesis

## Abstract

Macrocyclization is an important process that affords morphed scaffold in biosynthesis of bioactive natural products. Nature has adapted diverse biosynthetic strategies to form macrocycles. In this work, we report the identification and characterization of a small enzyme AvmM that can catalyze the construction of a 16-membered macrocyclic ring in the biosynthesis of alchivemycin A (**1**). We show through in vivo gene deletion, in vitro biochemical assay and isotope labelling experiments that AvmM catalyzes tandem dehydration and Michael-type addition to generate the core scaffold of **1**. Mechanistic studies by crystallography, DFT calculations and MD simulations of AvmM reveal that the reactions are achieved with assistance from the special tenuazonic acid like moiety of substrate. Our results thus uncover an uncharacterized macrocyclization strategy in natural product biosynthesis.

## Abstract (web summary)

Macrocyclization is an important process in bioactive natural product synthesis. Here, the authors report on the study of a macrocyclic ring constructing enzyme in the biosynthesis of alchivemycin A and using gene deletion, biochemical assays and isotope labelling show the enzyme catalyses tandem dehydration and Michael-type addition.

## Introduction

**Fig. 1:**
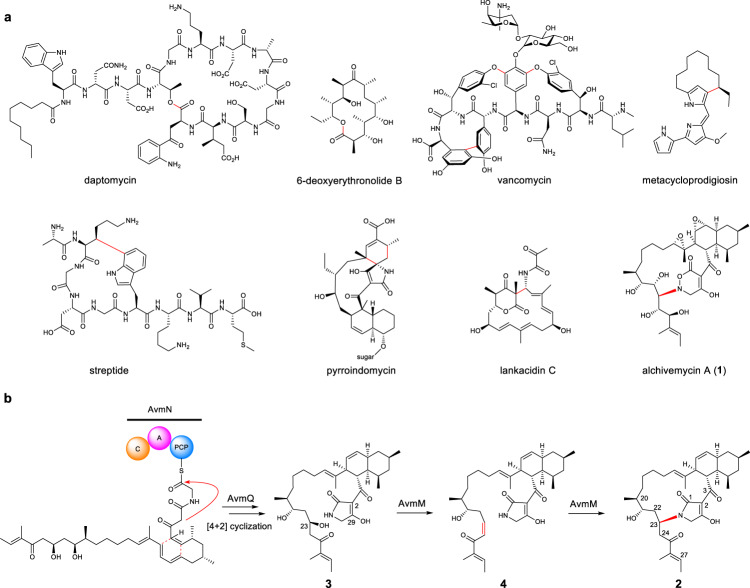
Macrocyclic reactions involved in natural product biosynthesis. **a** Representative macrocyclic natural products formed through diverse strategies. New bonds formed through macrocyclizations are highlighted in red. The macrocycles are formed by TE-tethering in daptomycin and 6-deoxyerythronolide B, a cytochrome P450 in vancomycin, a rieske oxygenase in metacloprodigiosin, a radical SAM in streptide, a dual function enzyme catalyzing an amide oxidation and Mannich reaction in lankacidin C, a Diels-Alderase in versipelostatin, and a new enzyme catalyzing a β-elimination and Michael addition in alchivemycin A. **b** Proposed macrocyclic mechanism for AVM (**1**). The configuration of C23 hydroxyl group was proposed based on sequence alignment of the corresponding KR domain and the existence of conserved “W” motif. The cis-geometry of C23-24 double bond was confirmed by the coupling constant.

Macrocyclization from linear precursors to cyclized products can afford constrained three-dimensional structures, which are important for their proper biofunctionality^1–3^. The thioesterase (TE)-mediated macrocyclization is regarded as the most canonical strategy in polyketide and nonribosomal peptide biosynthesis, where a hydroxyl or an amino group in the polyketide or peptide acyl chain acts as an internal nucleophile to attack the TE-tethered carbonyl carbon, generating a macrolactone or a macrolactam (Fig. 1a)^4–7^. Another effective way to form macrocycles is oxidative coupling catalyzed by P450, rieske oxygenase, or radical *S*-adenosyl-L-methionine (SAM) enzymes, as exemplified by the biosynthesis of vancomycin^8^, metacycloprodigiosin^9^ and streptide^10^. In addition, nature adapts a number of noncanonical enzymatic machineries to form diverse macrocycles. For instance: a [4 + 2] Diels-Alderase, PyrI4, is responsible for the macrocyclization of the aglycone of pyrroindomycin^11^ and its structurally-related compounds including versipelostatin, kijanimicin, and tetrocarcin^12–14^; a dual functional enzyme, LkcE, catalyzes a unique amide oxidation and a subsequent Mannich reaction to form the polyketide macrocycle in the lankacidin biosynthesis^15^. Therefore, understanding how nature generates macrocyclic molecules from acyclic precursors may provide new inspiration for the development of bio/synthetic methodologies (Fig. 1a).

Alchivemycin A (AVM, **1**) is a potent antibacterial polycyclic polyketide with an unprecedented skeleton originally isolated from a plant-derived actinomycete *Streptomyces* sp. TP-A0867. AVM contains a 17-membered macrocyclic ring, featuring a *cis*-decalin, a rare 2*H*-tetrahydro-4,6-dioxo-1,2-oxazine (TDO) moiety, and two epoxide rings^16^. AVM is biosynthetically derived from a hybrid *cis*-AT polyketide synthase-nonribosomal peptide synthetase (PKS-NRPS) pathway (Supplementary Fig. 1)^17^. Recently, we have elucidated the late-stage modification steps leading to the formation of **1** from a core structure **2**, in which six redox enzymes were characterized to install a TDO ring, two epoxide rings and three hydroxyl groups^18^. However, it remains elusive how the macrocyclic ring structure **2** is generated by PKS-NRPS megaenzymes and other accessory enzymes.

In this work, we report the characterization of an uncharacterized enzyme, AvmM, that is responsible for the macrocyclization of AVM ring through an unprecedented β-elimination and Michael-addition type mechanism.

## Results and discussion

**Fig. 2:**
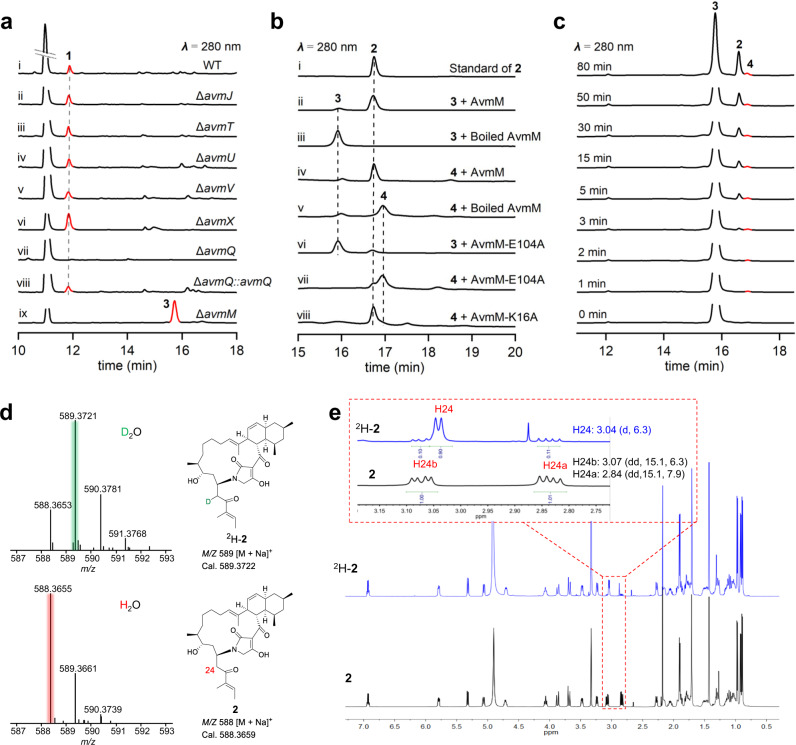
Characterization of AvmM as a macrocyclase. **a** HPLC analysis of metabolic extracts from *S*. sp. TP-A0867 wild-type and mutant strains. **b** In vitro enzyme assays of **3** or **4** incubated with the wild-type or mutant AvmM constructs. The conversion rates from **3** or **4** to **2** are as follow: ii) ~90%; iii) 0%; iv) ~100%; v) 0%; vi) ~11%; vii) 15%; viii) ~100%. **c** Time course in vitro assays of **3** with L182A mutant AvmM. The newly identified intermediate **4** is colored as red. **d** LC-MS comparison of **2** and D_2_O labeled ^2^H-**2**. **e** Proton NMR comparison of **2** and D_2_O labeled ^2^H-**2**. These experiments are repeated at least twice with similar results.

To map the steps between the nascent PKS product and **2** (Fig. 1b), we analyzed the *avm* biosynthetic gene cluster (BGC) in depth and turned our attention to enzymes with unassigned functions. Besides PKS-NRPS (AvmA-F, and AvmN), six redox (AvmO1, AvmO2, AvmO3, AvmO4, AvmP, and AvmR) and two RNA polymerases (AvmK and AvmL), the *avm* BGC encodes four hypothetical proteins (AvmJ, AvmM, AvmT, and AvmU), four ribosomal proteins (AvmS1, AvmS2, AvmS3 and AvmS4), one phosphotransferase (AvmV), one Dieckmann cyclase (AvmQ)^19^, and a XRE family transcriptional regulator (AvmX) (Supplementary Fig. 1a)^18^. We thus knocked out these genes individually through homologous recombination. Deletion of *avmJ*, *avmT*, *avmU* and *avmV* had no effects on the production of **1** (Fig. 2a, traces ii–v, Supplementary Fig. 2). The production of **1** in the *avmX* (transcriptional regulator) mutant strain was slightly increased in comparison to that of the wild-type (Fig. 2a, trace vi). AvmQ showed moderate sequence homology (43%/99%, identity/coverage) to TrdC, a Dieckmann cyclase that drives tetramic acid formation in tirandamycin B biosynthesis (Supplementary Fig. 3)^19^. Gene inactivation of *avmQ* completely abolished the production of **1** without accumulation of any metabolites (Fig. 2a, trace vii). Complementation of *avmQ* gene into the Δ*avmQ* mutant partially (~80 % compared to WT) restored the production of **1** (Fig. 2a, trace viii), suggesting that AvmQ is essential in AVM biosynthesis. AvmQ most likely functions as a Dieckmann cyclase and catalyzes the formation of tetramic acid, leading to the release of the product from the PKS-NRPS assembly line (Fig. 1b).

The only remaining gene in the cluster, *avmM*, encodes a small protein with 197 amino acid residues. A BLAST search of AvmM in the NCBI database resulted in no hits (Supplementary Fig. 4), while a further structural search with Phyre^2^ server showed homology to the partial sequence of a conjugative transposon lipoprotein (Supplementary Fig. 5). To understand the exact role of AvmM, we deleted the *avmM* gene. The mutant abolished the production of **1**, and resulted in the accumulation of a new metabolite **3** with *m*/*z* = 606.3763 ([M + Na]^+^) (Fig. 2a, trace ix and Supplementary Fig. 6). After a large-scale (5-L) fermentation, **3** was purified and characterized by extensive analysis of NMR data (Supplementary Table 6 and Supplementary Figs. 33–38). The molecular formula of **3** was determined to be C_35_H_53_NO_6_, which is 18 Da greater than that of **2**. The ^1^H NMR spectrum is almost identical to that of **2**, except for the appearance of a *N*H signal at *δ*_H_ 7.79 (brs), which showed HMBC correlations with C-2 and C-29, indicating the presence of a tetramic acid moiety. A hydroxyl group is proposed to be attached at C-23 based on its chemical shift value at *δ*_C-23_ 69.7, in line with the proposed molecular formula. Thus, **3** is determined to be an acyclic molecule (Fig. 1b). The absolute configuration of C-23 in **3** is predicted to be *S*, as its corresponding ketoreductase (KR) contains a diagnostic W-motif for A-type KR that could stereoseletiviely produce L-β-hydroxyl group (Supplementary Figs. 1b, 7)^18,20^. To verify if **3** is a biosynthetic pathway intermediate, we knocked out *avmA* gene in the wild-type strain. The resulting Δ*avmA* mutant strain was not able to produce **1**. When **3** was supplied into Δ*avmA* mutant, the production of **1** could be restored, indicating that **3** is an on-pathway intermediate en route to **1** (Supplementary Fig. 8).

To confirm the function of AvmM, we overexpressed AvmM in *Escherichia coli* BL21(DE3) and purified it to homogeneity with an N-terminal His_6_-tag (Supplementary Fig. 9a). Incubation of **3** with AvmM in the phosphate buffer (pH 7.0) led to the conversion to **2**, whereas the control reaction using boiled AvmM did not yield any new peaks (Fig. 2b, traces i–iii, Supplementary Fig. 10), indicating that AvmM is a macrocyclase capable of converting **3** to **2** with the concomitant loss of one molecule of H_2_O. Given the 23-OH is at the β-position of C-25 ketone carbonyl group, the β-elimination and subsequent Michael addition was proposed as the possible mechanisms for the AvmM-catalyzed macrocyclization. To investigate the reaction mechanism of AvmM, we performed the AvmM-catalyzed reaction in the deuterium water substituted phosphate buffer (D_2_O/H_2_O = 9:1, pH 7.0). The mass increment of +1 Da at 589.3721 for the enzymatic product **2** was detected (Fig. 2d), indicating that one hydrogen in **2** originated from water. To further assign the deuterium position, ^2^H-**2** was prepared via a large-scale enzymatic reaction. The ^1^H NMR spectrum of ^2^H-**2** showed the proton signal at H-24a had almost completely disappeared, and the coupling pattern for H-24b in ^2^H-**2** became a doublet instead of a double doublet in the original **2** (Fig. 2e). These results indicated that the deuterated position in ^2^H-**2** is H-24a. Our data support a mechanism by which AvmM catalyzes a β-elimination step on **3** to yield a C23 = C24 double bond, followed by a subsequent nucleophilic attack by the N atom of tetramic acid to the β-carbon (C-23) to generate an enolate intermediate. A general acid will then donate a proton to the α-carbon (C-24) and afford the macrocyclic **2** (Fig. 1b).

**Fig. 3:**
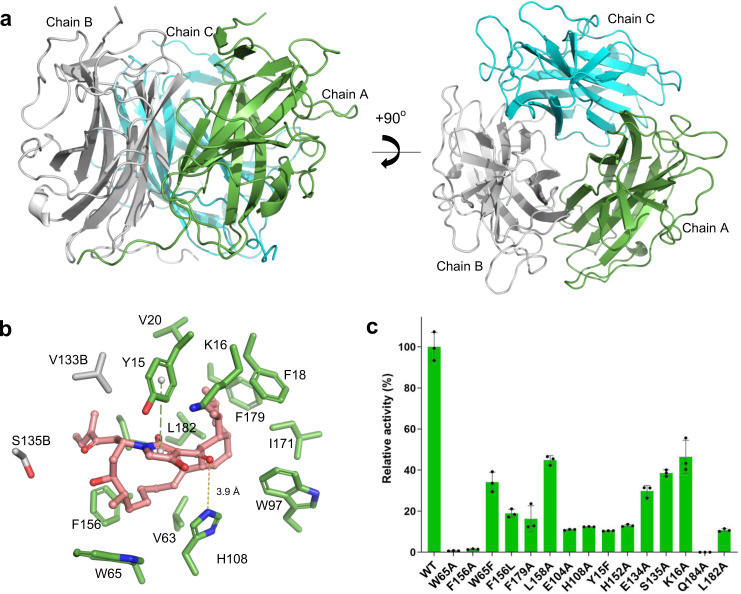
Crystal structures of AvmM. **a** Cartoon representations of the homotrimeric AvmM. **b** Close-up active-site view of co-crystal structure of the AvmM active site with product **2**. The hydrogen bonding and π–π stacking interaction are presented by gold dash line and green dash line. **c** Relative activity of AvmM and its site-specific mutants on enzymatic reactions. *n* = 3 biologically independent experiments; Data are presented as mean values + /− SD and error bars represent SD (standard deviation).

To understand the structural details of the AvmM-catalyzed macrocyclization, we set out to characterize the crystal structure of AvmM. However, AvmM shows no sequence homology to proteins with known structures, and it contains only two Met residues, which prevented us from using SAD (single-wavelength anomalous diffraction) phasing methods to determine its structure (Supplementary Fig. 4). Therefore, we generated a double mutant variant AvmM (L60M/L113M) to incorporate two additional selenomethionines (Se-Met), enhancing the anomalous signal and showing no impact on the activity (Supplementary Fig. 11). Consequently, the structure of AvmM was determined by SAD phasing using a Se-Met derivative crystal, and the resolution was extended to 2.09 Å resolution in a native crystal (Supplementary Table 7). Consistent with the apparent molecular weight (66.2 kDa) determined by the gel-filtration analysis (Supplementary Fig. 9), AvmM was characterized as a homotrimer (Fig. 3a), with each monomer adopting a jelly roll fold^21,22^. The overall monomeric structure comprises of eight main β-strands arranged in two parallel four-stranded sheets, and three small β sheets. Using the DALI search program^23^, AvmM shares low structural similarities to some components of large proteins, all of which adopted a jelly roll fold (Supplementary Fig. 12). Additionally, AvmM has a long C-terminal loop, which interacts with adjacent monomers to stabilize its quaternary structure (Supplementary Fig. 13).

**Fig. 4:**
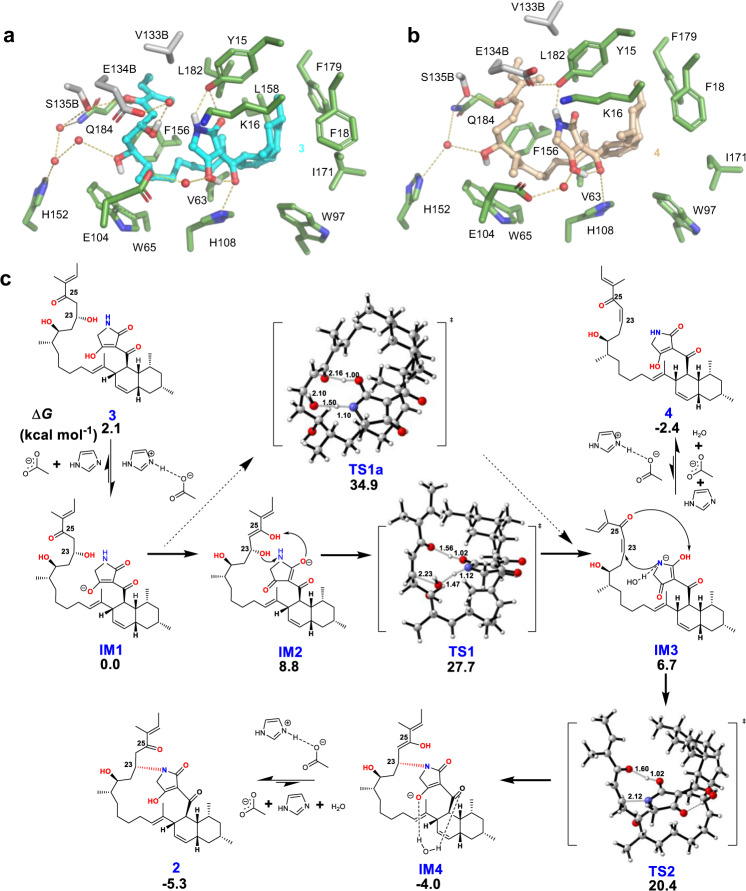
MD and DFT analysis. **a**, **b** Close-up view of MD representative snapshots of AvmM active site complexed with **3** and **4**. Water molecules are shown in red spheres and hydrogen bonds are in gold dash lines. **c** DFT-computed Gibbs free energies (in kcal mol^−1^) at the CPCM(water)-B3LYP-D3/6-311 + +G(2d,p)//CPCM(water)-B3LYP-D3/6-31 + G(d) level of theory and transition state structures (carbon: gray, hydrogen: white, oxygen: red, nitrogen: blue, and distances are shown in Å).

Exhaustive efforts to get AvmM in complex with the substrate **3** by optimizing protein/ligand concentrations, pH values, temperatures, precipitant concentration or even rescreening the crystal condition were not successful, probably due to the relatively low binding affinity (Supplementary Fig. 14). However, we were able to obtain the complex structure of AvmM with the product **2** in a resolution of 2.5 Å (Supplementary Table 7). The overall structure of AvmM-**2** complex is highly similar to the AvmM apo-form with a r.m.s.d. value of 0.18 Å for 574 Cα atoms (Supplementary Fig. 15a). The enhanced polder omit map shows a clear electron density for product **2** in the active site (Supplementary Fig. 16). The rigid *cis*-decalin system interacts with F18, W97, I171, F179 and L182 through hydrophobic interaction, while H108 shows a weak hydrogen bonding with C3 carbonyl group (Fig. 3b). Additionally, residue Y15 was found to form a T-shaped π–π stacking interaction with the tetramic acid moiety (Fig. 3b). Furthermore, analyzing the *B*-factor of AvmM structures indicated that the loop1 and loop2 in chain A are highly flexible, and clear deviations of Y15, K16 in loop1 and E104, H108 in loop2 were observed by comparing chains A between the *apo* and complex structures (Supplementary Fig. 17). To gain deeper insights into the reaction mechanism about how AvmM accommodates the highly flexible side chain of **3** and forges the C-N bond to yield **2**, the substrate **3** optimized by density functional theory (DFT) calculations (Supplementary Fig. 18a) was docked into the identified active site cavity by AutoDock Vina^24^, resulting in the well fit conformation with a highest affinity (Fig. 4a). We further performed molecular dynamics (MD) simulations to gain the dynamically stable AvmM-**3** complex structure (Supplementary Fig. 18b). Importantly, the orientation of the docked substrate **3** is highly consistent with **2** in the active site of AvmM-**2** complex structure (Supplementary Fig. 15b). The *cis*-decalin of **3** was surrounded by a series of hydrophobic residues including V63, W97, L158, F179 and L182; and the polyketide side chain curled in the opposite side of cavity, stretched deeply to occupy the inner active site pocket (Fig. 4a). This curled conformation is achieved via hydrophobic interactions with the nonpolar residues W65 and F156. The hydrophilic residues also interact with substrate **3** through an elegant hydrogen bond network. The chain B S135 of AvmM interacts with C-25 carbonyl, while chain B E134 form a water-mediated hydrogen bond with OH-23. In addition, the tetramic acid moiety forms hydrogen bonds with H108 and Y15. More importantly, the OH-23 hydroxyl is in a *syn* periplanar to the pro-*R* hydrogen at C-24 (Supplementary Fig. 19), thus after *syn*-elimination, the specific twisted **3** would generate a *Z*-type C23 = C24 double bond. Interestingly, comparison of the docking result with crystal complex suggested that the formation of 16-member ring causes the polyketide side chain to move away from Chain B of the protein, disrupting the hydrogen bonds observed in the docking results.

To verify the observed interactions, we performed site-directed mutagenesis. Substitution of two hydrophobic residues W65 and F156 to Ala almost completely abolished activity, while W65F and F156L variants could partially rescue their activity (Fig. 3c), supporting the idea that W65 and F156 may provide hydrophobic interactions to the side chain. Mutations of other two hydrophobic residues F179 and L158, which surrounded the *cis*-decalin, also only remained 16% and 45% activity. Consistent with our simulations, mutation of E104 and H108 dramatically decreased the activity of AvmM to 11% and 12% (Fig. 2b, trace vi, Fig. 3c), confirming their roles as key catalytic residues. The activity of the Y15F, H152A, E134A, S135A and K16A mutants were also significantly decreased to only 10%-46%, while Q184A mutant completely lose the activity, highlighting the importance of the hydrogen bond network for AvmM activity (Fig. 3c). We further performed the size exclusion chromatography (SEC) and circular dichroism (CD) analysis to confirmed that the Q184 mutations have no impact on the overall structure (Supplementary Fig. 9d, e). In addition, L182A mutant only retains 11% activity compared to the wild-type, but interestingly, it generated a new product **4** that has 18 Da (H_2_O) less than **3** (Fig. 2c), based on its deduced molecular formula C_35_H_51_NO_5_ as judged by the HRESIMS data *m/z* 588.3655 [M + Na]^+^ (calculated for C_35_H_51_NO_5_Na^+^, 588.3659). However, due to its inherent instability and extremely low conversion rate, we were only able to purify ~0.2 mg **4** from a large-scale enzymatic reaction. The overall ^1^H NMR of **4** is similar to that of **3** (Supplementary Figs. 40–42). The apparent differences are in the presence of two coupled olefinic protons at *δ*_H_ 6.29 (dt, *J* = 11.5, 7.9 Hz, H23) and *δ*_H_ 6.68 (d, *J* = 11.5 Hz, H24) in **4**, and the absence of an oxygenated proton signal at *δ*_H_ 4.28 corresponding to H23 in **3** (Supplementary Figs. 40–42). Moreover, the HSQC spectrum showed that *δ*_H_ 6.29 and *δ*_H_ 6.68 protons are attached to the carbons at *δ*_C_ 144.0 and *δ*_C_ 126.6, respectively (Supplementary Figs. 43–46), indicating that the newly generated double bond is conjugated with a carbonyl group^25,26^. This assignment is further supported by the ^1^H-^1^H COSY correlations of *δ*_H_ 3.58 (H21)/*δ*_H_ 2.64 (H22)/*δ*_H_ 6.29 (H23)/*δ*_H_ 6.68 (H24) (Supplementary Figs. 43–46). Thus, **4** is a dehydrated Δ^23^ derivative of **3** (Fig. 1b). It is noteworthy that the geometry of C23 = C24 double bond was deduced as *Z* on the basis of *J-*coupling constant (*J*_H23/H24_ = 11.5 Hz). Incubation of **4** with AvmM led to total conversion to **2** (Fig. 2b, traces iv–v), supporting that **4** is an on-pathway intermediate to **2**. Docking of **4** into the AvmM active site showed it possesses an almost identical orientation to **3** (Supplementary Fig. 15c). Further analysis of E104A mutant by using **4** as substrate revealed that the activity was also significantly decreased to ~15% (Fig. 2b, trace vii), suggesting that E104 is critical for both dehydration and Michael addition steps, while the K16A showed no effect on its activity (Fig. 2b, trace viii).

In the canonical dehydration step in PKS, a histidine residue acts as a base to deprotonate the α-proton of substrate, then re-donates the proton to the β-hydroxy group to promote elimination of water^27^. Intriguingly, within the 5 Å range of substrate **3**, there are no residues that could function as a general base to abstract the α-proton (H-24) in **3**. Therefore, the dehydration step catalyzed by AvmM might be distinct from the reaction catalyzed by canonical dehydratase in PKS. To reveal the unique reaction mechanism, we performed DFT calculations. Based on the MD structure of AvmM**-3** complex, we found that pre-organized active site residues anchor the closed conformation of **3** and orient the tetramate moiety approaching the OH-23. Specially, E104 and H108 form direct or water-mediated hydrogen bonds with the tenuazonic acid like moiety, respectively (Fig. 4a). The tenuazonic acid like moiety of **3** is in a deprotonated form in buffer of pH 7.0, since its p*K*a is estimated to be less than 4^28^. In the presence of acetate ion and imidazole (a simplified model of Glu and His), the deprotonation is spontaneous and computed to be exergonic by 2.1 kcal mol^−1^ in water (Supplementary Fig. 20a). Further MD analysis indicated that the distance between E104 and H108 is about 10 Å and a water-mediated hydrogen network was observed (Supplementary Fig. 20b, c), suggesting that E104 and H108 may act as a general acid/base pair via a proton relay to abstract the proton to initiate the reaction. After deprotonation, the amide in the anionic intermediate **IM1** could function as a general acid to protonate the OH-23, and meanwhile, the C1 carbonyl group could act as a general base to abstract the α-proton (H-24) to complete the dehydration through a substrate-assisted acid/base catalysis (Fig. 4c). However, the computed energy barrier for the direct proton transfer from the C24 to eliminate the OH-23 by the amide via transition state **TS1a** is as high as 34.9 kcal mol^−1^, which in principle rules out the possibility (Fig. 4c). Alternatively, ketone **IM1** can tautomerize to enol **IM2** that is then converted into the transient intermediate **IM3** via transition state **TS1** (Fig. 4c). The calculated activation barrier of 27.7 kcal mol^−1^ for this pathway means that this transformation may occur in the enzyme. Reviewing the apo crystal structure of AvmM and MD snapshot of AvmM-**3** complex (Fig. 4a), we observed that the C25 carbonyl group in **3** forms a proton relay mainly with H152 and Q184 in chain A and S135 and E134 in chain B of AvmM, as well as the amide of **3** with Y15 through hydrogen bonds. We hypothesized that these interactions might stabilize the intermediate and transition state and further lower the energy barrier. As expected, our calculations revealed that the binding of acetamide and phenol could reduce the elimination barrier by 2.1 and 1.7 kcal mol^−1^, respectively (Supplementary Fig. 21). This supports that active site residues, especially Q184 and Y15, can accelerate the reaction, making it proceed smoothly at room temperature. Subsequently, **IM3** is either re-protonated to the isolated compound **4** or nucleophilically attacks on the β-carbon of α, β-unsaturated ketone with the proton transfer via transition state **TS2** to obtain **IM4**, which is finally protonated to generate product **2**, similar to other glycosylation and methylation on the amine group^29–31^.

**Fig. 5:**
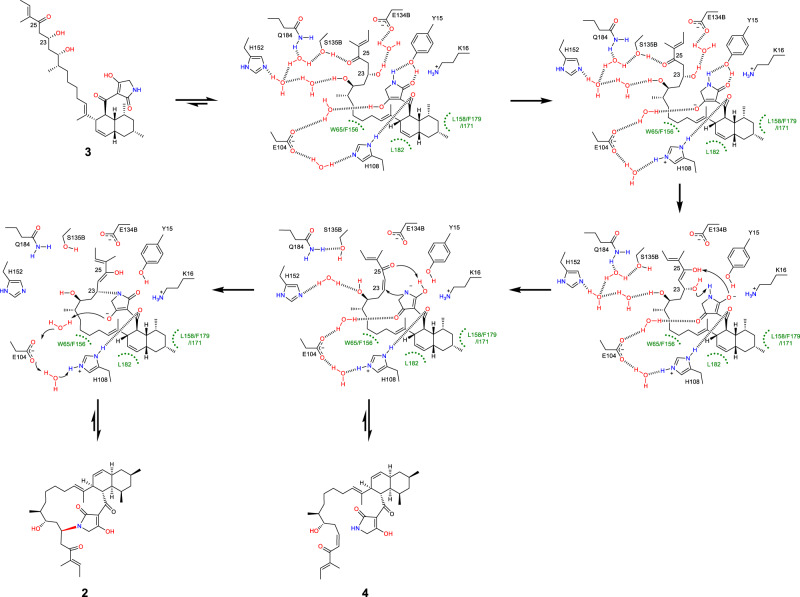
Proposed reaction mechanism for AvmM. The bold dashed line indicates the putative hydrogen bonds. E104 and H108 are linked by three water molecules and only one is shown for clarity.

Thus, the crystal structures of AvmM, together with molecular docking, MD simulations, DFT calculations and site-directed mutagenesis, allow us to propose a putative catalytic mechanism (Figs. 4c and 5). Once **3** enters the active site of AvmM, the *cis*-decalin ring and the polyketide chain would be clamped by a series of hydrophobic residues. The hydrophilic residues in the active site also interact with **3** through hydrogen bonds. The C-25 ketone group and tetramate moiety on **3** form hydrogen bonds with S135B and Q184 as well as Y15, respectively. Initiated by the key residue of E104 and H108, a consequent β-elimination reaction will take place to yield the intermediate **IM3** with the assistance of the tenuazonic acid like moiety of **3**. After re-protonation mediated by E104 and H108, the isolated and characterized **4** would be generated. A subsequent Michael addition of the N atom to the β-carbon (C23) will occur to give an enolate intermediate, which can be further transformed to **2** by accepting a proton from a general acid. The formation of the 16-membered macrocyclic ring makes **2** more rigid and dramatically changes the conformation of flexible side chain in **3**. Finally, due to the loss of hydrogen bond interactions on the side chain, the release of product **2** is facile to finish the catalytic cycle.

In summary, our in vivo gene deletion combined with our in vitro biochemical assay, crystallographic analyses, DFT calculations and MD simulations uncovered a novel macrocyclization strategy in natural product biosynthesis through tandem β-elimination and Michael addition with assistance from the special moiety in substrate. Although a growing number of enzymes capable of catalyzing Michael-type additions for constructing six-membered carbocyclic and heterocyclic rings have been observed^32–35^, AvmM represents a unique novel enzyme that can catalyze the formation of a 16-membered macrocyclic ring. Our findings expand the catalytic repertoire for naturally occurring macrocyclases, and set the stage for further macrocyclase bioengineering.

## Methods

### General experimental procedures

All 1D and 2D NMR experiments were measured on a Bruker Avance III 400 MHz or a Bruker Avance 600 MHz. UV spectra were obtained on a Nanodrop 2000 spectrometer (Thermo Scientific, USA) with a 10 mm cuvette. High resolution LC-MS data were measured on an Agilent 6530 TOF LC-MS spectrometer with a Porshell 120 EC-C18 column (4.5 × 50 mm, 2.7 μm, Agilent Techonologies). Optical rotation values were collected in methanol on a Rudolph Autopol IV automatic polarimeter. Semipreparative RP-HPLC was conducted on an Agilent 1260 HPLC system with a DAD detector equipped with an Eclipse XDB-C18 column (C-18, 9.4 × 250 mm, 5 µm, Agilent Techonologies). PCR amplifications were performed on a Bio-Rad S1000™ Thermal Cycler. Recombinant proteins were purified on a GE ÄKTA pure chromatography system with a 5 mL Histrap HP column (GE lifesciences).

### Gene inactivation and complementation

Double cross-over homologous recombination was used for genes (*avmJ*, *avmX*, *avmT*, *avmU*, *avmV*, *avmM* and *avmQ*) disruption. In order to construct the plasmid for inactivation of the target gene, the upstream and downstream homology arms were amplified with up-F/R primers and down-F/R primers (Supplementary Table 2) using genomic DNA of *Streptomyces* sp. TP-A0867 as template. Then, the amplified upstream and downstream fragments were ligated with the plasmid pKC1139 linearized by *Hin*dIII and *Eco*RI, to generate knockout plasmid. The individual plasmid was then conjugated into *Streptomyces* sp. TP-A0867 using the standard procedure^36^. After cultured for 5 days at 30 °C, the colonies with apramycin resistant were transferred to MS plates supplied with apramycin antibiotics (50 μg/mL). The apramycin-sensitive colonies were picked as candidate for double-crossover mutants, and the target mutant strain was obtained through subculture and screening. The correctness of these candidate clones was confirmed by diagnostic PCR analysis using the primers listed in Supplementary Table 2.

To complement *avmQ* into the mutant strain Δ*avmQ*, the fragment containing *avmQ* was amplified with 152-AvmQ-F/R primers (Supplementary Table 2) using genomic DNA of *Streptomyces* sp. TP-A0867 as a template, and was ligated with the linearized pSET152-*KasO*p* (digested by *Nde*I and *Eco*RI), to give the recombinant plasmid. The resulting plasmid was transformed into Δ*avmQ* strain by conjugation to afford Δ*avmQ::avmQ* strain with apramycin resistant.

### Fermentation and analysis of intermediates

*Streptomyces* sp. TP-A0867 and mutant strains were inoculated into 250-mL flasks containing 50 ml V22 medium (soluble starch 1%, glucose 0.5%, NZ-case 0.3%, yeast extract 0.2%, Tryptone 0.5%, K_2_HPO_4_ 0.1%, MgSO_4_·7H_2_O 0.05% and CaCO_3_ 0.3%) at 30 °C and 200 rpm for 2 days. The seed culture was then inoculated into 250-mL flasks containing 50 mL production medium (glucose 0.2%, soluble starch 2.5%, polypeptone 0.5%, yeast extract 0.5%, NZ-amine 0.5%, and XAD-16 resin 1%) at 30 °C and cultivated for 6 days. Then, the fermentation broth was extracted with ethyl acetate and concentrated for subsequent experiments.

The LC-MS analysis was performed with a 20 min gradient elution system from 20% to 100% (1–13 min), 100% (13–17 min) and 10% (17–20 min) acetonitrile using Porshell 120 EC-C18 column (4.5 × 50 mm, 2.7 μm) in water supplied with 0.1% formic acid at a flow rate of 0.5 mL/min.

### Isolation of the compound 3

To chracterize compound **3** accumulated in the mutant strain, a 5-L large-scale fermentation for Δ*avmM* strain was carried out using the fermentation contidtion described above. The fermentation broth was extracted with ethyl acetate, and then concentrated under reduced pressure. The crude extract was fractionated on a Sephadex LH20 column. The object fraction was separated by a semi-preparative HPLC using a linear gradient system from 20% to 100% (1–13 min), 100% (13–35 min) acetonitrile/water at a flow rate of 3 mL/min to give **3** (12.5 mg, t_*R*_ = 32.0 min).

### Isolation of the compound ^2^H-2 and 4

To obtain sufficient ^2^H-**2**, a 5 mL-total-volume (D_2_O:H_2_O = 9:1) large scale enzymatic reactions were performed. The solution contained 50 mM phosphate buffer (pH 7.0), 1.2 mM **3** and 40 μM AvmM. After incubation at 30 °C for 2 h, 5 mL acetonitrile was added to quench the reaction. Compound ^2^H-**2** (1.1 mg, t_*R*_ = 41.0 min) was purified from the concentrated reaction mixture by semi-preparative HPLC using 80% acetonitrile in H_2_O at a flow rate of 2.5 mL/min.

To get sufficient **4**, a 50 mL-total-volume large scale enzymatic reactions were performed. The solution contained 50 mM PBS buffer (pH 7.0), 1 mM **3** and 10 μM AvmM-L182A. After incubation at 30 °C for 1 h, 5 mL acetonitrile was added to quench the reaction. Compound **4** (0.2 mg, t_*R*_ = 32.5 min) was purified from the concentrated reaction mixture by semi-preparative HPLC using a gradient elution system from 20% to 100% (1–18 min), 100% (18–35 min) acetonitrile at a flow rate of 3 mL/min.

### Physicochemical data of compound 3

Compound **3**, white solid; [α]^25^_D_ + 74.1 (*c* 0.21, MeOH); UV (MeOH) λ_max_ (log *ε*) 285 (4.04), 229 (4.08); HRESIMS (positive) *m/z* 606.3763 [M + Na]^+^ (calcd for C_35_H_53_NO_6_Na, 606.3765). ^1^H and ^13^C NMR data see Supplementary Table 6.

### Protein expression and purification

DNA fragments containing target gene of *avmM* was amplified from genomic DNA of *Streptomyces* sp. TP-A0867 with primers listed in Supplementary Table 2. The purified PCR product was ligated with linearized pET28a (linearized by NdeI and HindIII) to afford pHG8026. The pHG8026 was further introduced into *E*. *coli* BL21(DE3). The transformant was cultivated in 400 mL LB medium at 37 °C (220 rpm) until OD_600_ value reached around 0.4–0.6. The culture was cooled to 4 °C and induced with 0.125 mM IPTG, continued to cultivate at 16 °C (220 rpm) for 18 h. After centrifugation at 10,000 x *g* for 10 min, cells were resuspended in 25 mL lysis buffer (100 mM Tris, pH 8.0, 15 mM imidazole, 300 mM NaCl, 10% glycerol) and lysed on ice by sonication. After centrifugation at 22,000 x *g* for 30 min, the supernatant was filtered and purified by ÄKTA FPLC system equipped with a 5 mL Histrap HP column (GE lifesciences).The proteins were pooled and desalted by a PD10 column (GE Healthcare) with 100 mM phosphate buffer (pH 8.0) and 10% glycerol and stored at −80 °C.

For selenomethionine (SeMet)-labeled AvmM, a mutated plasmid AvmM (L60M/L113M) was genereate for selenomethionine incorpration and then the plasmid was transfomed into BL21(DE3) *E. coli* cells. The fresh BL21(DE3) harbouring corresponding plasmid was cultured overnight at 37 °C in 5 mL of LB medium containing 50 µg/mL kanamycin. Second day, the dense seed culture was tranfered into 400 mL fresh M9 minimal medium and incubated at 37 °C, 220 rpm until an OD_600_ of 0.6 was reached. After addition of 100 mg/L lysine, threonine, phenylalanine; 50 mg/L leucine, isoleucine, valine and 50 mg/L SeMet, incubation was continued for another 15 min. Finishing all steps mentioned above, the expression and purification of SeMet-substituted AvmM (L60M/L113M) was the same as normal protein, except for addition of 5 mM *β*-mercapto ethanol in all used buffers.

### In vitro assay of AvmM

The AvmM and mutants catalyzed reactions were performed in a 100 μL reaction system containing 50 mM PBS buffer (pH 7.0), 100 μM substrate **3** or **4**, 10 μM AvmM. After incubation at 30 °C for 5 min, 100 μL acetonitrile was added to quench the reaction. Then, the reaction mixture was centrifuged at 22,000 x *g* for 10 min, and the supernatant was subjected to LC-MS analysis. The LC-MS analysis was performed with a 25 min gradient elution system from 20% to 100% (1–13 min), 100% (13–22 min) and 20% (22–25 min) acetonitrile using Porshell 120 EC-C18 column (4.5 × 50 mm, 2.7 μm) in water supplied with 0.02% formic acid at a flow rate of 0.5 mL/min. The time course analysis of AvmM was performed in a similar way, except that the temperature was changed to room temperature.

### Analytical size-exclusion chromatography

The molecular weights (MWs) and quaternary state of AvmM and other Q184 mutated proteins in solution were determined by size-exclusion chromatography using a HiLoad^TM^ 16/600 Superdex^TM^ 200 pg column (GE Healthcare Life Sciences) connected to an ÄKTA Express system (GE Healthcare Life Sciences). The column was pre-equilibrated with two column volumes of 50 mM NaCl, 20 mM Tris buffer, pH 8.0, and calibrated with cytochrome c (13.7 kDa), ovalbumin (44 kDa), Aldolase (160 kDa), and ferritin (440 kDa). The chromatography was carried out at 4 °C at a flow rate of 1 mL/min. The column void volume was determined by using Blue Dextran as standard. The calibration curve of *K*av versus log (MW) was prepared using *K*av = (*V*_e_ - *V*_o_)/ (*V*_t_ - *V*_o_), where *V*_e_, *V*_o_, and *V*_t_ is the elution volume, column void volume, and total bed volume, respectively.

### D_2_O labeling experiments

The AvmM catalyzed reaction was carried out in a 100 μL reaction system (D_2_O:H_2_O = 9:1) containing 50 mM PBS buffer (pH 7.0), 100 μM substrate **3**, 10 μM AvmM. After incubation at 30 °C for 5 min, 100 μL acetonitrile was added to quench the reaction. The LC-MS analysis was performed with a 25 min gradient elution system from 20% to 100% (1–13 min), 100% (13–22 min) and 20% (22–25 min) acetonitrile using Porshell 120 EC-C18 column (4.5 × 50 mm, 2.7 μm) in water supplied with 0.02% formic acid at a flow rate of 0.5 mL/min.

### Chemical complementation of compound 3 into Δ*avmA* mutant

The Δ*avmA* mutant were cultured in a 50 mL scale at 30 °C in fermentation medium. After 48 h cultivation, compound **3** (0.5 mg, 10 mM) dissolved in DMSO were individually supplemented into fermentation broth individually and cultured for another 48 h. The metabolic extract was analyzed by LC-MS. The LC-MS analysis was performed with a 20 min gradient elution system from 20% to 100% (1–13 min), 100% (13–17 min) and 20% (17–20 min) acetonitrile using Porshell 120 EC-C18 column (4.5 × 50 mm, 2.7 μm) in water supplied with 0.1% formic acid at a flow rate of 0.5 mL/min.

### Protein crystallization and structure elucidation

A broad screening of crystallization conditions was performed for AvmM using a sitting-drop method in MRC2D plate. The best crystal of AvmM used for data collection was obtained by mixing 0.5 μL of the protein (10 mg/mL in 50 mM NaCl, 20 mM Tris, pH 8.0) and 0.5 μL of reservoir (0.2 M Magnesium acetate tetrahydrate, 0.1 M Sodium cacodylate pH 6.5 and 20% w/v PEG 8000). Crystals appeared after seven days at 22 °C. The SeMet-substituted AvmM (L60M/L113M) was crystalized the same as above. For crystallization of AvmM-**2** complex, purified AvmM was incubated with saturated **2** in 50 mM NaCl, 20 mM Tris, pH 8.0 at 4 °C for 4 h and then the crystals were obtained the same as before. To further increase the occupancy of **2**, tip full powder of **2** was add into the reservoir together with crystals for 5 h. Finally, the obtained crystals were briefly soaked in the crystallization buffer containing additional 20% glycerol before flash-freezing for protection.

One set of single-wavelength anomalous diffraction data for SeMet-AvmM (L60M/L113M) was collected at BL17U1 beamline at the Shanghai Synchrotron Radiation Facility (SSRF) at wavelengths of 0.97897 Å, whereas data for AvmM and AvmM-**2** complex were collected at BL18U1 beamline at SSRF at wavelengths of 0.97853 Å. All diffraction datasets collected were processed and scaled using iMosflm^37^. The Se-SAD phase was determined and a partial structural model of Se-AvmM (L60M/L113M) was traced in PHENIX. AutoSol^38^. The structural of Se-AvmM (L60M/L113M) was initially built with PHENIX.Auto-Build and then built manually with Coot^39^ and then refined with PHENIX^40^. Subsequently, AvmM and AvmM-**2** complex were solved with molecular replacement using Se-AvmM (L60M/L113M) as search model. Finally, additional TLS refinement was performed in PHENIX. The final refinement statistics are listed in Supplementary Table 7. For enhancing the electron density map, a PHENIX. Polder map was utilized with omission of ligand region^41^. Structural diagrams were prepared using the program PyMOL (http://www.pymol.org/).

### Biolayer interferometry analysis

The Octet RED96 System (fortéBio, Pall Life Science) was used to measure the binding kinetics of AvmM proteins to the substrate **3** and product **2**. The binding is measured by the shift of wavelength due to the interaction between the two molecules on the surface of the biosensor. All assays were carried out at 30 °C with continuous 1000 rpm shaking and 200 µL per well for each solution. PBS with 0.1% BSA, 0.02% Tween-20 and 0.2% DMSO was used as the assay buffer. AvmM proteins were tethered on Ni-NTA biosensors (ForteBio) by dipping sensors into protein solutions. The final protein concentration is 19.67 µM. Average saturation response levels of 8–9 nm were achieved in 5 min for AvmM protein. Sensors with proteins tethered were washed in assay buffer for 10 min to eliminate nonspecifically bound protein molecules and establish stable base lines before starting association-dissociation cycles with test compound. DMSO only references were included in all assays. Raw kinetic data collected were processed in the Data Analysis software provided by the manufacturer using double reference subtraction in which both DMSO only reference and inactive reference were subtracted. Octet data was analyzed and processed using the Octet data analysis 11.1 software.

### Docking experiments

The docking experiments were performed using AutoDock Vina 1.1.2^24^. The ligands were generated by sketcher in ccp4^42^ and edited and optimized by Phenix, REEL^43^, eLBOW^44^ and Gaussian 16 package^45^. AutoDock-Tools 1.5.6 was used to prepare the ligands and macromolecule AvmM. The default parameters were used to set the torsion constraints for **3** and **4**, and charges and hydrogen atoms were added to AvmM proteins.

### DFT calculations

The DFT calculations were performed with the Gaussian 16 package^45^. The geometry optimizations of minima and transition states involved were carried out at the B3LYP-D3/6-31 + G(d) level. The vibrational frequency calculations were calculated at the same level to ensure that all of the stationary points were transition states (one imaginary frequency) or minima (no imaginary frequency) and to evaluate zero-point vibrational energies (ZPVE) and thermal corrections at 298 K. Single-point energy calculations were performed at the B3LYP-D3 level with the 6-311 + +G(2d,p) basis set. Solvation by water was taken into account by using the CPCM model^46–48^ for all above calculations. Gibbs free energy is the sum of the electronic energy and ZPVE and thermal corrections.

### Molecular dynamics simulations

All molecular dynamics (MD) simulations were performed by Amber 16 package^49^. The docking structures of AvmM complexed with **3** and **4** were used as the starting conformations for MD simulations on the protein-ligand complexes with Se-Met 60, 113 and 161 mutated back to Leu, Leu and Met, respectively. The protonation states of charged residues were determined at constant pH 7.0 based on pKa calculations via the H + + 4.0 program^50^ and the consideration of the local hydrogen bonding network. All His residues were assigned as HIE. All Asp and Glu residues were deprotonated, while Lys and Arg were protonated. **3** and **4** were fully optimized at the B3LYP-D3/6-31 + G(d) level of Gaussian 16 using the CPCM model^47–49^ in water, and the partial charges were fitted with HF/6-31 G(d) calculations and the restrained electrostatic potential (RESP)^51,52^ protocol implemented by the Antechamber module. The force field parameters for **3** and **4** were adapted from the standard general amber force field 2.0 (gaff2)^53^ parameters, while the standard Amber14SB force field was applied to describe the protein. Each system was neutralized by adding Na^+^ ions and solvated into a truncated octahedron TIP3P^5^ water box with a 10 Å buffer distance on each side. These two systems consisted of 37004 and 37016 atoms for AvmM with **3** and **4**, respectively. After equilibrated with a series of minimizations interspersed by short MD simulations during which restraints on the protein backbone heavy atoms were gradually released (with force constant of 10, 2, 0.1 and 0 kcal/(mol·Å^2^)), each system was heated from 0 to 310 K for 50 ps in which harmonic potentials were used to positionally restrain the protein backbone heavy atoms (with force constant of 10 kcal/(mol·Å^2^)). Finally, 30 ns MD simulations with periodic boundary condition at constant temperature and pressure were carried out in which the protein backbone heavy atoms were restrained with a force constrant of 5 kcal/(mol·Å^2^) during the first 10 ns and then the rest 20 ns were unrestrained. The pressure was maintained at 1 atm and coupled with isotropic position scaling. The temperature was controlled at 310 K with Berendsen thermostat method. Long-range electrostatic interactions were treated with particle mesh Ewald (PME)^54^ method and 12 Å cutoff was applied to both PME and van der Waals (vdW) interactions. Time step of 2 fs was employed along with SHAKE algorithm for hydrogen atoms, and periodic boundary condition was used. Each system was checked for stability (structure, energy, and temperature fluctuations) and convergence (root mean square deviations-RMSD of structures).

### Site-directed mutagenesis of AvmM

For mutagenesis of AvmM, a quick change site-directed mutagenesis method was applied. Mutated fragments were amplified with primers listed in Supplementary Table 2 by using plasmid pHG8031 as template. The purified PCR products were incubated with DpnI, T4 polynucleotide kinase and T4 DNA ligase, according to the standard procedure of Q5^®^ Site-Directed Mutagenesis Kit purchased from NEB (USA). Each mutation was confirmed by sequencing. The recombined plasmids were expressed in *E. coli* BL21(DE3) and purified as described above for native protein.

However, for mutagenesis of Y15F, W65F, E134A, S135A, F156L, Q184K and Q184N, the primers are designed with overlaps. The amplified circular mutant fragments can be directly transferred into *E. coli* DH5*α*. After verified by sequencing and correct mutants are transferred to *E. coli* BL21(DE3) for expression and purification.

### Reporting summary

Further information on research design is available in the Nature Research Reporting Summary linked to this article.

## Supplementary information

A Supplementary Information file

Reporting Summary

## Data Availability

Data supporting the findings of this work are available within the paper and its Supplementary Information files. A reporting summary for this Article is available as a Supplementary Information file. The Atomic coordinates data of AvmM generated in this study have been deposited in the Protein Data Bank (PDB) database under accession code 7FE0, 7FE5 and 7FE6. The source data underlying Figs. 2a–c, 3c; and Supplementary Figs. 6a, 8a, 9b, 9e, 10, 14 are provided as a Source Data file. Source data are provided with this paper.

## Source data

Source Data
