# Supplementary material for: AvmM catalyses macrocyclization through dehydration/Michael-type addition in alchivemycin A biosynthesis
Source: Nat Commun. 2022 Aug 3;13:4499. doi: 10.1038/s41467-022-32088-4 (PMC9349299; doi:10.1038/s41467-022-32088-4)
Supplement: Supplementary file 1 — A Supplementary Information file [file 41467_2022_32088_MOESM1_ESM.pdf]

Supporting Information

AvmM Catalyses Macrocyclization through Dehydration/Michael-type Addition in Alchivemycin A Biosynthesis

Hong Jie Zhu,[1,#] Bo Zhang,[1,#] Wanqing Wei,[2,#] Shuang He Liu,[1] Lang Xiang,[1] Jiapeng Zhu,[3] Rui Hua Jiao,[1] Yasuhiro Igarashi,[4] Ghader Bashiri,[5] Yong Liang,[2,*] Ren Xiang Tan,[1,*] Hui Ming Ge[1,*]

[1]State Key Laboratory of Pharmaceutical Biotechnology, Chemistry and Biomedicine Innovation Centre, Institute of Artificial Intelligence Biomedicine, School of Life Sciences, Nanjing University, Nanjing 210023, China

[2]State Key Laboratory of Coordination Chemistry, Jiangsu Key Laboratory of Advanced Organic Materials, Chemistry and Biomedicine Innovation Centre, School of Chemistry and Chemical Engineering, Nanjing University Nanjing 210023 (China)

[3]State Key Laboratory Cultivation Base for TCM Quality and Efficacy, School of Medicine and Life Sciences, Nanjing University of Chinese Medicine, Nanjing 210023, China

[4]Biotechnology Research Center and Department of Biotechnology, Toyama Prefectural University, Toyama 939-0398, Japan

[5]Laboratory of Molecular and Microbial Biochemistry, School of Biological Sciences, The University of Auckland, Auckland 1010, New Zealand

[#]These authors contributed equally: Hong Jie Zhu, Bo Zhang, Wanqing Wei.

*email: yongliang@nju.edu.cn; rxtan@nju.edu.cn; hmge@nju.edu.cn

Supplementary Tables
Supplementary Table 1. Bacterial plasmids and strains.
Supplementary Table 2. Primers used in this study.
Supplementary Table 3. Annotation of the *avm* cluster.
Supplementary Table 4. ^{1}H (400 MHz) and ^{13}C NMR (100 MHz) data of **2** (δ in ppm, J in Hz, methanol-d_4).
Supplementary Table 5. ^{1}H (600 MHz) and ^{13}C NMR (150 MHz) data of **2** (δ in ppm, J in Hz, acetone-d_6).
Supplementary Table 6. ^{1}H (600 MHz) and ^{13}C NMR (150 MHz) data of **3** (δ in ppm, J in Hz, acetone-d_6).
Supplementary Table 7. Data collection and refinement statistics of AvmM-Se, AvmM and AvmM-**2** complex.
Supplementary Figures
Supplementary Figure 1. Biosynthesis of alchivemycin A (**1**).
Supplementary Figure 2. Gene in-frame deletion in *Streptomyces* sp. TP-A0867.
Supplementary Figure 3. Alignment of AvmQ with the reported and possible secondary sequence of Dieckmann cyclase.
Supplementary Figure 4. Database comparison result of AvmM.
Supplementary Figure 5. The top 10 results given in the Phyre^{2} protein structure comparison.
Supplementary Figure 6. HPLC/MS analysis of metabolic extracts from *S*. sp. TP-A0867 wild-type and *avmM* mutant strain.
Supplementary Figure 7. Analysis of catalytic residues of *avm* KR domains of PKS.
Supplementary Figure 8. LC-MS analysis of chemical complementation of compound **3** into the Δ*avmA* mutant strain.
Supplementary Figure 9. SDS page and size-exclusion chromatography (SEC) of the purified proteins.
Supplementary Figure 10. Time-course analysis of AvmM catalyzed reaction.
Supplementary Figure 11. In vitro assays of AvmM and AvmM-L60M/L113M.
Supplementary Figure 12. The crystal structure of AvmM monomer with other similar proteins searched by Dali sever.
Supplementary Figure 13. The crystal structure of AvmM.
Supplementary Figure 14. Analysis of the interaction between AvmM and product **2** (top panels) or substrate **3** (lower panels) by biolayer interferometry.
Supplementary Figure 15. Comparison of docking and complex results.
Supplementary Figure 16. Electron density of the **2** in the AvmM co-crystal structure.
Supplementary Figure 17. Flexibility analysis of AvmM.
Supplementary Figure 18. MD and DFT analysis of AvmM and substrate **3** and **4**.
Supplementary Figure 19. Proposed mechanism of dehydration step catalyzed by AvmM.
Supplementary Figure 20. The computed Gibbs free energies (in kcal mol^{-1}) of **3** interacted with H108 (imidazole) or/and E104 (acetate ion) and MD analysis.
Supplementary Figure 21. The computed Gibbs free energies (in kcal mol^{-1}) of **IM1** and **TS1** stablized by Q184 (acetamide) or Y15 (phenol).
Supplementary Figure 22. ^{1}H NMR spectrum of **2** in methanol-d_4 (600 MHz).
Supplementary Figure 23. ^{1}H NMR spectrum of ^{2}H-**2** in methanol-d_4 (600 MHz).
Supplementary Figure 24. Comparison of ^{1}H NMR spectra of **2** and ^{2}H-**2** in methanol-d_4 (600 MHz).
Supplementary Figure 25. ^{13}C NMR spectrum of ^{2}H-**2** in methanol-d_4 (150 MHz).
Supplementary Figure 26. HSQC NMR spectrum of ^{2}H-**2** in methanol-d_4.
Supplementary Figure 27. Partially amplified HSQC NMR spectrum of ^{2}H-**2** in methanol-d_4.
Supplementary Figure 28. ^{1}H NMR spectrum of **2** in acetone-d_6 (600 MHz).
Supplementary Figure 29. ^{13}C NMR spectrum of **2** in acetone-d_6 (150 MHz).
Supplementary Figure 30. ^{1}H-^{1}H COSY NMR spectrum of **2** in acetone-d_6.
Supplementary Figure 31. HSQC NMR spectrum of **2** in acetone-d_6.
Supplementary Figure 32. HMBC NMR spectrum of **2** in acetone-d_6.
Supplementary Figure 33. ^{1}H NMR spectrum of **3** in acetone-d_6 (600 MHz).
Supplementary Figure 34. ^{13}C NMR spectrum of **3** in acetone-d_6 (150 MHz).
Supplementary Figure 35. DEPT-135 NMR spectrum of **3** in acetone-d_6 (150 MHz).
Supplementary Figure 36. ^{1}H-^{1}H COSY NMR spectrum of **3** in acetone-d_6.
Supplementary Figure 37. HSQC NMR spectrum of **3** in acetone-d_6.
Supplementary Figure 38. HMBC NMR spectrum of **3** in acetone-d_6.
Supplementary Figure 39. HR-ESIMS spectrum of **3**.
Supplementary Figure 40. ^{1}H NMR spectrum of **4** in acetone-d_6 (600 MHz).
Supplementary Figure 41. Comparison of ^{1}H NMR spectra of **3** and **4** in acetone-d_6 (600 MHz).
Supplementary Figure 42. Comparison of NMR data of compound **3** and **4**.
Supplementary Figure 43. ^{1}H-^{1}H COSY NMR spectrum of **4** in acetone-d_6.
Supplementary Figure 44. HSQC NMR spectrum of **4** in acetone-d_6.
Supplementary Figure 45. Partially amplified ^{1}H-^{1}H COSY NMR spectra of **4** in acetone-d_6.

Supplementary Figure 46. Partially amplified HSQC NMR spectrum of **4** in acetone-d_6.
Supplementary Figure 47. HR-ESIMS spectrum of **4**.
Coordinates
Supplementary References

Supplementary Table 1. Bacterial plasmids and strains.

Plasmid/strain	Relevant characteristics	Reference
**Plasmid**		
pKC1139	*E. coli*-*Streptomyces* shuttle plasmid used for gene disruption, temperature sensitive	1
pSET152-*kasO*p*	pSET152 derived plasmid containing the promoter *kasO*p*	2
pET-22b(+)	Protein expression vector used in *E. coli*, encoding C-terminal His-tag, ampicillin resistance	Novagen
pET-28a(+)	Protein expression vector used in *E. coli*, encoding N-terminal His-tag, kanamycin resistance	Novagen
pHG8023	pKC1139 derived plasmid for disruption of *avmJ*	This study
pHG8024	pKC1139 derived plasmid for disruption of *avmX*	This study
pHG8025	pKC1139 derived plasmid for disruption of *avmT*	This study
pHG8026	pKC1139 derived plasmid for disruption of *avmU*	This study
pHG8027	pKC1139 derived plasmid for disruption of *avmV*	This study
pHG8028	pKC1139 derived plasmid for disruption of *avmM*	This study
pHG8029	pKC1139 derived plasmid for disruption of *avmQ*	This study
pHG8030	pSET152-*kasO*p* derived plasmid for complementation of *avmQ* in HG8016	This study
pHG8031	pET-28a(+) derived plasmid for expressing N-terminal His-tag AvmM	This study
***E. coli* strains**		
DH5*α*	General cloning host	3
BL21 (DE3)	Heterologous host for protein expression	NEB
ET12567/pUZ8002	Methylation-deficient host used for *E. coli-Streptomyces* intergeneric conjugation	1
***S. seoulensis* strains**		
Streptomyces sp. TP-A0867	Wild type strain for alchivemycin A (**1**) production	4
HG8001	Δ*avmA*, in-frame deletion mutant strain in WT, **1** non-producing	5
HG8010	Δ*avmJ*, in-frame deletion mutant strain in WT, **1** producing	This study
HG8011	Δ*avmX*, in-frame deletion mutant strain in WT, **1** producing	This study
HG8012	Δ*avmT*, in-frame deletion mutant strain in WT, **1** producing	This study
HG8013	Δ*avmU*, in-frame deletion mutant strain in WT, **1** producing	This study
HG8014	Δ*avmV*, in-frame deletion mutant strain in WT, **1** producing	This study
HG8015	Δ*avmM*, in-frame deletion mutant strain in WT, **1** non-producing	This study
HG8016	Δ*avmQ*, in-frame deletion mutant strain in WT, **1** non-producing	This study
HG8017	complementation of Δ*avmQ* mutant by *avmQ*, **1** producing	This study

Supplementary Table 2. Primers used in this study.

Name	Sequence	Enzyme sites
**a. for amplification of homologous arms from genomic DNA for gene disruption (5’-3’)**		
Δ*avmQ*-UP-F	AACGACGGCCAGTGCCAAGCTTCGGAGATCCTGGAGGCGA	*Hin*dIII
Δ*avmQ*-UP-R	CGAAGCGGATCTCGCGCGGCCGGTGAAGGGGTAGT	
Δ*avmQ*-Down-F	CGCGCGAGATCCGCTTCG	
Δ*avmQ*-Down-R	AGCTATGACATGATTACGAATTCCGGAACCCTCCCCCGAAC	*Eco*RI
Δ*avmM*-UP-F	AACGACGGCCAGTGCCAAGCTTTGAAACGGCAAGACGATGT	*Hin*dIII
Δ*avmM*-UP-R	GTAGAAGAGCTTGAAGTCCTCCGAACCAGATGACCAGGTTGAG	
Δ*avmM*-Down-F	GGAGGACTTCAAGCTCTTCTAC	
Δ*avmM*-Down-R	AGCTATGACATGATTACGAATTCCCTCGGAGGTGTTCAGTTC	*Eco*RI
Δ*avmJ*-UP-F	AACGACGGCCAGTGCCAAGCTTCGCATGGACCTGACGAA	*Hin*dIII
Δ*avmJ*-UP-R	TTCTTCACCAGCAGGTTCTGCTCTTCTCGCCCGTCTTG	
Δ*avmJ*-Down-F	CAGAACCTGCTGGTGAAGAA	
Δ*avmJ*-Down-R	AGCTATGACATGATTACGAATTCGGCGCCATTGTTCGTATTG	*Eco*RI
Δ*avmX*-UP-F	AACGACGGCCAGTGCCAAGCTTCGGCCGTTCATGGAATCAC	*Hin*dIII
Δ*avmX*-UP-R	ACTTTGACTCCATCTCTTCCAAGTGCGCAAGATCATGGAGAG	
Δ*avmX*-Down-F	CTTGGAAGAGATGGAGTCAAAGT	
Δ*avmX*-Down-R	AGCTATGACATGATTACGAATTCGCCGTGGTTGTCGATCAT	*Eco*RI
Δ*avmT*-UP-F	AACGACGGCCAGTGCCAAGCTTCCCAGCTCGTAGCGATTG	*Hin*dIII
Δ*avmT*-UP-R	ATCTTGCCTACGCCGTTGCTCGCAGATGCCAGCTC	
Δ*avmT*-Down-F	CAACGGCGTAGGCAAGAT	
Δ*avmT*-Down-R	AGCTATGACATGATTACGAATTCATCCACGAACACCCGAAC	*Eco*RI
Δ*avmU*-UP-F	AACGACGGCCAGTGCCAAGCTTCGGTGAAGATCGGCGTATT	*Hin*dIII
Δ*avmU*-UP-R	CAACGCAGAGGGAGACCGTTCTCCGTGGTGCAGTT	
Δ*avmU*-Down-F	GGTCTCCCTCTGCGTTG	
Δ*avmU*-Down-R	AGCTATGACATGATTACGAATTCGCAGGAGACTGACCACATT	*Eco*RI
Δ*avmV*-UP-F	AACGACGGCCAGTGCCAAGCTTCAACGGCGTAGGCAAGATC	*Hin*dIII
Δ*avmV*-UP-R	CGTGGTTGTCGATCATGATGTCAGAGCGATGGTGCAGTT	
Δ*avmV*-Down-F	ACATCATGATCGACAACCACG	
Δ*avmV*-Down-R	AGCTATGACATGATTACGAATTCGCGGGGCGACTTGAGGC	*Eco*RI
**b. for screening of the double-crossover mutants (5’-3’)**		
ScreenΔ*avmQ*-F	CGATCGAATTCTCGGTCTTCTAC	
ScreenΔ*avmQ*-R	CATCAGGTGTGTCCTCTCAAC	
ScreenΔ*avmM*-F	GTTCCCGTACCCGACTTTC	
ScreenΔ*avmM*-R	TCGATCACCTTGTGGAACTG	
ScreenΔ*avmJ*-F	GAAGGGCCGCTACATCAA	
ScreenΔ*avmJ*-R	GAAGGAAGGAGACTGAGACAAC	
ScreenΔ*avmX*-F	GATGAGTCTGTCGGCGTTTA	
ScreenΔ*avmX*-R	GTCGGCGAGTTCTGTGAG	
ScreenΔ*avmT*-F	GGAAGAGATGGAGTCAAAGTCTC	
ScreenΔ*avmT*-R	GTTCTCCGTGGTGCAGTT	
ScreenΔ*avmU*-F	CAACGGCGTAGGCAAGAT	
ScreenΔ*avmU*-R	CAGAGCGATGGTGCAGTT	
ScreenΔ*avmV*-R	GTGGGTCGCTGGTGTTC	
ScreenΔ*avmV*-R	CGAGCAGGTGCCGAAAG	
**c. for genes introduction (5’-3’)**		
152-AvmQ-F	ACGTACTAGTCTGACATATGACCGATCTCCGCCTTCCACTG	*Nde*I
152-AvmQ-R	CTATGACATGATTACGAATTCCCGCGCACCCTGAAGGTG	*Eco*RI
**d. for protein expression (5’-3’)**		

28a-AvmM-F	GGTGCCGCGCGGCAGCCATATGACGTCCACGGTCTCCACCGA	*Nde*I
28a-AvmM-R	GCTCGAGTGCGGCCGCAAGCTTTCAGTAGAGGGAGGACGGGC	*Hin*dIII
28a-AvmM-Y15F-F	GTGAGTTCAAGGGTTTCCGGGTCA	
28a-AvmM-Y15F-R	CCCTTGAACTCACGGTAGACCGGC	
28a-AvmM-K16A-F	CCGTGAGTACGCCGGTTTCCGGGTCAATGACAACATC	
28a-AvmM-K16A-R	TAGACCGGCCCGTCGGTG	
28a-AvmM-W65A-F	CCTGGTCATCGCCTTCTCGGACCGGGACGATCTGC	
28a-AvmM-W65A-R	TTGAGCCCGGTGTCCGGG	
28a-AvmM-W65F-F	TCATCTTCTTCTCGGACCGGGACG	
28a-AvmM-W65F-R	GAGAAGAAGATGACCAGGTTGAGC	
28a-AvmM-E104A-F	GTTCCCCGCCGCCGGCGGCGTCC	
28a-AvmM-E104A-R	TGCGGGTCCCAGCATTTCG	
28a-AvmM-H108A-F	GGGCGGCGTCGCCATCGCCTGCCAG	
28a-AvmM-H108A-R	TCGGCGGGGAACTGCGGG	
28a-AvmM-E134A-F	AGGTCGCCTCGGTCACCGCCCATG	
28a-AvmM-E134A-R	ACCGAGGCGACCTCCGGCAGCGGG	
28a-AvmM-S135A-F	TCGAGGCCGTCACCGCCCATGTGC	
28a-AvmM-S135A-R	GTGACGGCCTCGACCTCCGGCAGC	
28a-AvmM-H152A-F	CACCTCCGAGGCCGCGGGCATCT	
28a-AvmM-H152A-R	ATCCCTTCACGGAAGGCC	
28a-AvmM-F156A-F	GCCGCGCTGCCGGGCATGCTG	
28a-AvmM-F156A-R	GATGCCCGCGTGCTCGGA	
28a-AvmM-F156L-F	GCATCCTGGCGCTGCCGGGCATG	
28a-AvmM-F156L-R	AGCGCCAGGATGCCCGCGTGCTC	
28a-AvmM-L158A-F	GCCCCGGGCATGCTGGACGCG	
28a-AvmM-L158A-R	CGCGAAGATGCCCGCGTGC	
28a-AvmM-F179A-F	GCCGGCCGGCTCCAGCAGGCC	
28a-AvmM-F179A-R	GACATTGCCGAACAGACCG	
28a-AvmM-L182A-F	GCCCAGCAGGCCAGCTTCCGGC	
28a-AvmM-L182A-R	CCGGCCGAAGACATTGCCG	
28a-AvmM-Q184A-F	CCGGCTCCAGGCCGCCAGCTTCC	
28a-AvmM-Q184A-R	CCGAAGACATTGCCGAAC	
28a-AvmM-Q184K-F	TCCAGAAGGCCAGCTTCCGGCTGG	
28a-AvmM-Q184K-R	CTGGCCTTCTGGAGCCGGCCGAA	
28a-AvmM-Q184N-F	TCCAGAATGCCAGCTTCCGGCTGG	
28a-AvmM-Q184N-R	CTGGCATTCTGGAGCCGGCCGAA	

Supplementary Table 3. Annotation of the *avm* cluster[a]

Gene	Size[b]	Proposed function	Identity%/Coverage%	Accesion number
avmH	77	preprotein translocase subunit SecE	99/98	SEC40549.1
avmI	281	transcription termination/antitermination protein	92/99	CDR07753.1
avmS1	144	50S ribosomal protein L11	98/99	EFG08697.1
avmS2	239	50S ribosomal protein L1	97/99	AGP51590.1
avmJ	296	hypothetical protein D3C57_121880	91/99	RLV81079.1
avmS3	187	50S ribosomal protein L10	86/93	KDN76512.1
avmS4	129	50S ribosomal protein L7/L12	97/99	AGP55862.1
avmK	1160	DNA-directed RNA polymerase subunit beta	96/99	AGP55861.1
avmL	1299	DNA-directed RNA polymerase subunit beta´	98/99	AGP55860.1
avmO1	527	FAD-dependent monooxygenase	40/94	CAK50794.2
avmO2	511	FAD-dependent monooxygenase	46/99	L8EUQ6.1
avmM	197	hypothetical protein M271_21665	97/99	AGP55857.1
avmQ	270	dieckmann cyclase	43/99	ADY38535.1
avmN	1117	non-ribosomal peptide synthetase	60/92	AVV61987.1
avmP	405	cytochrome P450	48/96	2ZBX_A
avmR	294	F420-dependent oxidoreductase	25/55	Q46FV4.1
avmA	3107	type I polyketide synthase	87/93	AGP55852.1
avmB	2890	type I polyketide synthase	63/83	AGP60456.1
avmC	3731	type I polyketide synthase	89/99	AGP55849.1
avmD	7654	type I polyketide synthase	86/99	RLV81094.1
avmE	4354	type I polyketide synthase	87/97	AGP55844.1
avmF	3637	type I polyketide synthase	83/57	AGP55841.1
avmO4	308	phytanoyl-CoA dioxygenase	26/85	5EPA_A
avmO3	535	FAD-dependent monooxygenase	42/97	L8EUQ6.1
avmX	416	XRE family transcriptional regulator	90/93	AGP55634.1
avmT	200	hypothetical protein M271_33565	72/95	AGP58127.1
avmU	156	hypothetical protein	53/63	MBP2064842.1
avmV	282	aminoglycoside phosphotransferase	87/91	OMI38235.1

[a]The sequence has been deposited in GenBank with the accession numbers MW490597 and MZ617354-MZ617366.
[b]Numbers are in amino acids.

Supplementary Table 4. ^{1}H (400 MHz) and ^{13}C NMR (100 MHz) data of **2** (δ in ppm, J in Hz, methanol-d_4).[5]

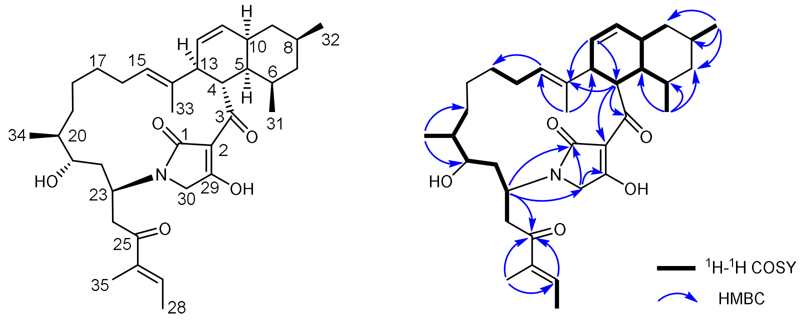

2 **2**

No.	δ_C	δ_H (J in Hz)
1	174.4, C	
2	105.0, C	
3	192.8, C	
4	38.3, CH	4.06 (t, 10.9)
5	43.8, CH	2.27 (m)
6	39.3, CH	1.79 (m)
7	39.3, CH_2	1.27 (m), 1.06 (m)
8	34.6, CH	1.51 (m)
9	39.3, CH_2	1.75 (m)
10	41.2, CH	2.16 (m)
11	134.2, CH	5.79 (ddd, 9.8, 5.3, 2.7)
12	129.7, CH	5.32 (br d,9.8)
13	54.8, CH	3.23 (br d,10.9)
14	135.8, C	
15	129.3, CH	5.06 (dd, 9.2, 2.0)
16	29.7, CH_2	2.06 (m), 1.94 (m)
17	30.6, CH_2	1.30 (m), 1.14 (m)
18	28.0, CH_2	1.04 (m), 1.01 (m)
19	34.9, CH_2	1.54 (m), 1.10 (m)
20	34.4, CH	1.75 (m)
21	68.0, CH	3.48 (ddd, 10.8, 4.6, 1.7)
22	37.2, CH_2	1.84 (overlap)
23	45.9, CH	4.71 (m)
24	41.9, CH_2	3.06 (dd, 15.1, 6.4), 2.83 (dd, 15.1, 7.9)
25	201.2, C	
26	139.5, C	
27	140.8, CH	6.93 (q, 6.9)
28	15.1, CH_3	1.92 (d, 6.9)
29	193.4, C	
30	51.8, CH_2	3.88 (d, 17.7), 3.68 (d,17.7)
31	21.5, CH_3	0.92 (d, 7.3)
32	22.9, CH_3	0.97 (d, 6.6)
33	12.5, CH_3	1.43 (s)
34	13.4, CH_3	0.87 (d, 6.9)
35	11.1, CH_3	1.71 (s)

Supplementary Table 5. ^{1}H (600 MHz) and ^{13}C NMR (150 MHz) data of **2** (δ in ppm, J in Hz, acetone-d_6).

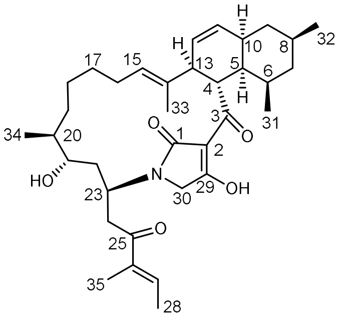

2

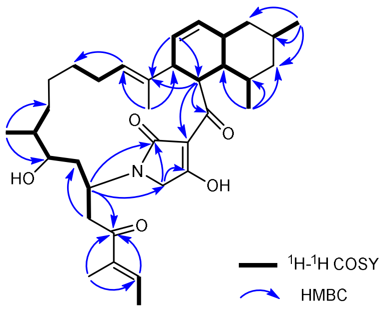

2

No.	δ_C	δ_H (J in Hz)
1	174.0, C	
2	104.6, C	
3	190.8, C	
4	37.6, CH	4.04 (t, 10.9)
5	43.1, CH	2.23 (m)
6	38.7, CH	1.78 (m)
7	38.9, CH_2	1.25 (m), 1.09 (m)
8	34.7, CH	1.49 (m)
9	38.9, CH_2	1.76 (m)
10	40.7, CH	2.14 (m)
11	133.9, CH	5.78 (ddd, 9.8, 5.2, 2.7)
12	129.4, CH	5.31(br d,9.8)
13	54.2, CH	3.21 (br d,10.9)
14	135.3, C	
15	128.7, CH	5.05 (br d, 10.2)
16	29.4, CH_2	2.04 (m), 1.92 (m)
17	30.0, CH_2	1.30 (m), 1.15 (m)
18	27.6, CH_2	1.06 (m), 1.03 (m)
19	34.1, CH_2	1.50 (m), 1.08 (m)
20	33.9, CH	1.79 (m)
21	66.9, CH	3.53 (m)
22	37.3, CH_2	1.84 (overlap)
23	45.0, CH	4.77 (m)
24	41.9, CH_2	3.17 (dd, 15.6, 7.5), 2.82 (dd, 15.6, 6.6)
25	199.3, C	
26	138.9, C	
27	139.0, CH	6.91 (q, 6.7)
28	15.0, CH_3	1.85 (d, 6.7)
29	191.8, C	
30	51.7, CH_2	3.90 (d, 17.4), 3.71 (d,17.4)
31	21.3, CH_3	0.90 (d, 7.1)
32	22.9, CH_3	0.94 (d, 6.5)
33	12.4, CH_3	1.40 (s)
34	13.5, CH_3	0.89 (d, 6.7)
35	11.2, CH_3	1.69 (s)

Supplementary Table 6. ^{1}H (600 MHz) and ^{13}C NMR (150 MHz) data of **3** (δ in ppm, J in Hz, acetone-d_6).

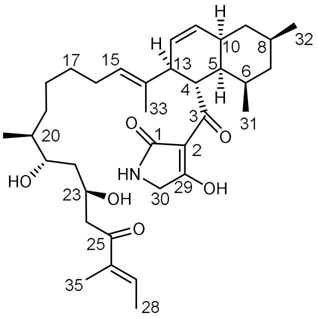

3

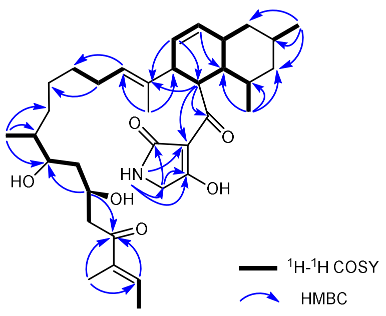

3

No.	δ_C	δ_H (J in Hz)
1	177.2, C	
2	104.5, C	
3	192.1, C	
4	37.9, CH	4.04 (t, 10.9)
5	42.3, CH	2.27 (dt, 10.9, 3.5)
6	38.6, CH	1.77 (m)
7	38.6, CH_2	1.27 (m), 1.07 (m)
8	34.0, CH	1.49 (m)
9	38.8, CH_2	1.71 (m), 1.09 (m)
10	40.3, CH	2.15 (m)
11	133.5, CH	5.81 (ddd, 9.9, 5.4, 2.5)
12	129.0, CH	5.36 (br d, 9.9)
13	53.2, CH	3.16 (br d, 10.9)
14	135.7, C	
15	128.1, CH	5.17 (t, 7.3)
16	28.5, CH_2	2.0 (m), 1.84 (m)
17	30.9, CH_2	1.19 (m)
18	27.6, CH_2	1.29 (m), 1.20 (m)
19	39.2, CH_2	1.51 (m)
20	33.5, CH	1.50 (m)
21	74.9, CH	3.71 (ddd, 10.8, 4.6, 1.7)
22	40.1, CH_2	1.63 (overlap)
23	69.7, CH	4.28 (m)
24	45.4, CH_2	2.91 (overlap), 2.86 (overlap)
25	201.2, C	
26	139.1, C	
27	139.1, CH	6.88 (q, 6.9)
28	14.9, CH_3	1.86 (d, 6.9)
29	193.3, C	
30	52.5, CH_2	3.80 (d, 17.7), 3.74 (d,17.7)
31	21.2, CH_3	0.92 (d, 7.3)
32	22.8, CH_3	0.94 (d, 6.5)
33	12.6, CH_3	1.48 (s)
34	14.6, CH_3	0.85 (d, 6.9)
35	11.0, CH_3	1.73 (s)
NH-1		7.79 (br s)

Supplementary Table 7. Data collection and refinement statistics of AvmM-Se, AvmM and AvmM-**2** complex.

	AvmM-Se	AvmM	AvmM-**2** complex
**Data collection**			
Space group	$P\ 4_1\ 2_1\ 2$	$P\ 4_1\ 2_1\ 2$	$P\ 4_1\ 2_1\ 2$
Cell dimensions			
a, *b*, *c* (Å)	73.9, 73.9, 243.6	73.8, 73.8, 244.7	73.5, 73.5, 242.4
α, *β*, *γ* (°)	90.0, 90.0, 90.0	90.0, 90.0, 90.0	90.0, 90.0, 90.0
Resolution (Å)	23.56 - 2.20 (2.27 - 2.20)	30.60 - 2.09 (2.15 - 2.09)	80.80 - 2.50 (2.60 - 2.50)
R_{merge} (%)	13.1 (119.9)	16.2 (71.7)	19.5 (182.1)
$I / \sigma I$	10.3 (2.2)	7.1 (2.3)	11.2 (1.9)
$CC_{1/2}$	1.00 (0.53)	0.99 (0.68)	1.00 (0.63)
Completeness (%)	99.7 (96.3)	99.3 (93.1)	100 (100)
Redundancy	11.7 (11.2)	8.8 (8.5)	15.8 (16.6)
**Refinement**			
Resolution (Å)	22.14 - 2.20 (2.28 - 2.20)	29.47 - 2.09 (2.17 - 2.09)	60.60 - 2.50 (2.59 - 2.50)
No. reflections	34646 (3321)	40242 (3691)	23921 (2322)
R_{work} / R_{free} (%)	18.68/21.87 (27.70/32.34)	20.30/24.80 (26.14/28.50)	18.96/24.20 (30.76/36.39)
No. atoms	4638	4751	4545
Protein	4431	4431	4431
Ligand/ion	8	6	48
Water	199	314	66
B-factors	49.7	40.2	60.2
Protein	49.8	40.1	60.2
Ligand/ion	92.3	82.0	68.7
Water	46.6	41.5	51.9
R.m.s. deviations			
Bond lengths (Å)	0.008	0.007	0.009
Bond angles (°)	1.22	0.90	1.18
**PDB ID**	7FE0	7FE5	7FE6

Values in parentheses are for highest-resolution shell.

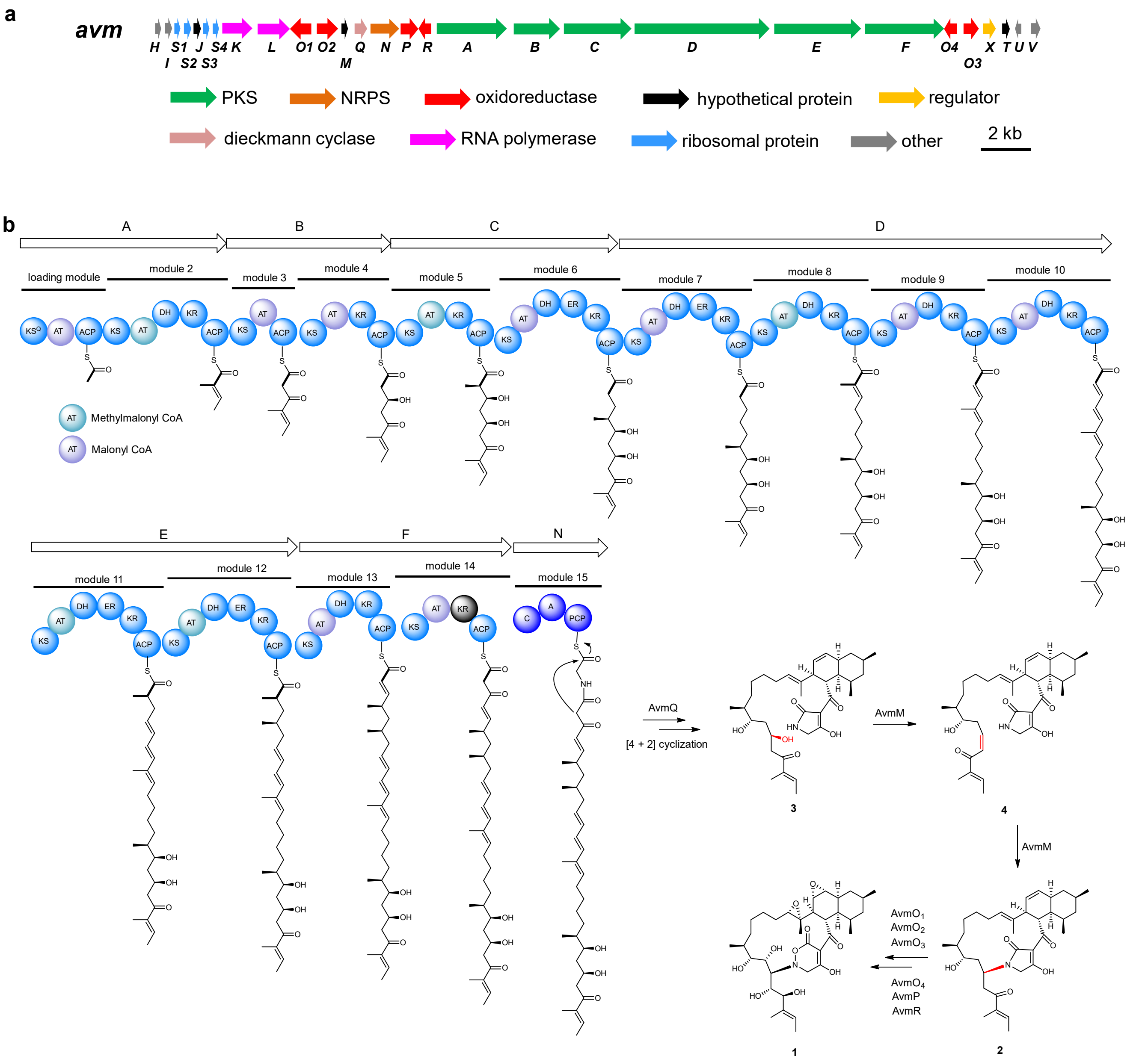

Supplementary Figure 1. Biosynthesis of alchivemycin A (**1**). **a,** Biosynthetic gene cluster of *avm* from *S.* sp. TP-A0867. **b,** Proposed biosynthetic pathway for **1**. KS, Ketosynthase domain; AT, Acetyltransferase domain; DH, Dehydratase domain; KR, Ketoreductase domian; ACP, Acyl carrier protein domain; ER, Enoylreductase domain; C, Condensation domain; A, Adenylation domain; PCP, Peptide carrier protein domain. The light blue circle presents PKS domains, while the dark blue circle presents NRPS domains. The black circle indicates the KR domain in module 14 is non-function.

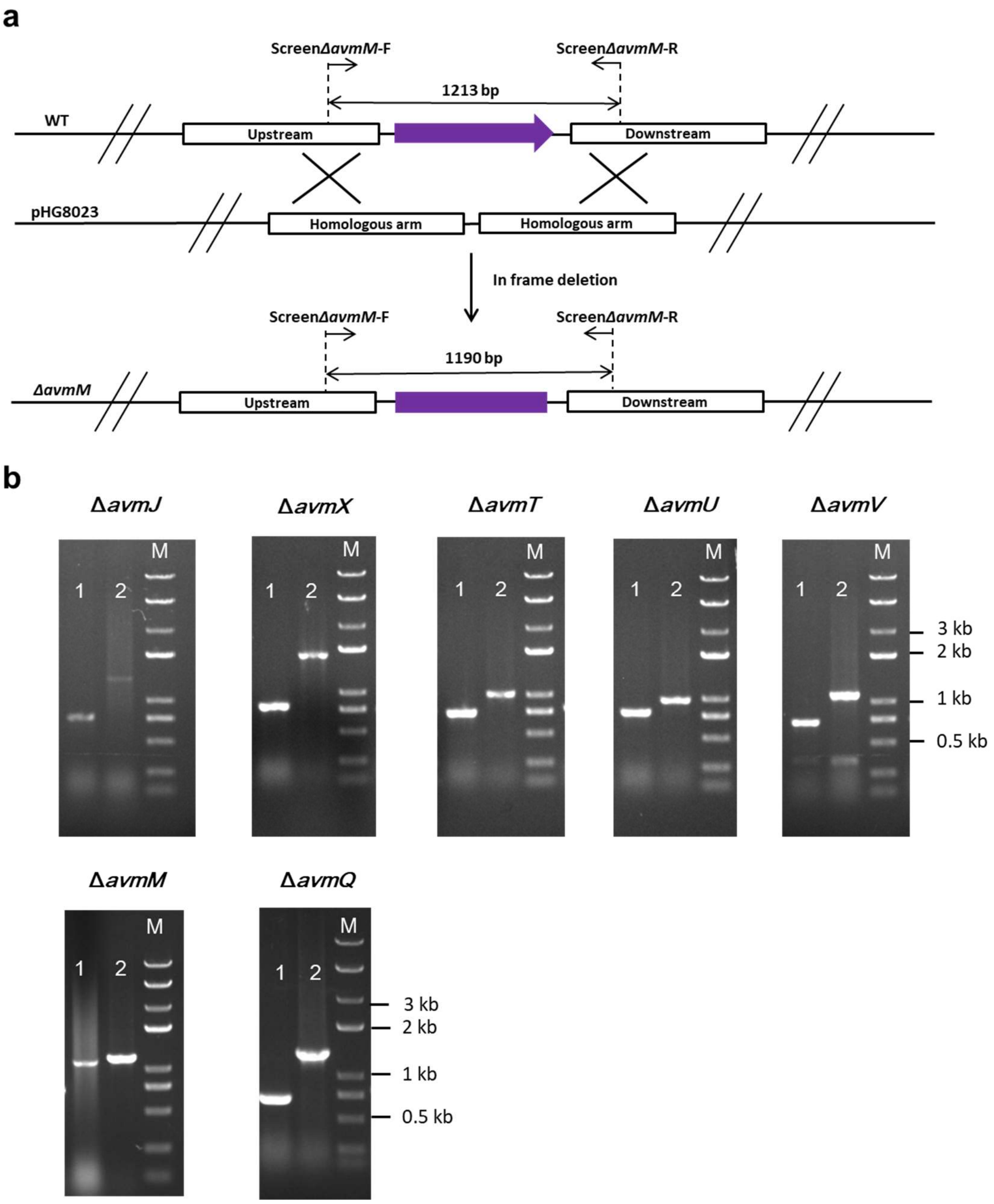

Supplementary Figure 2. Gene in-frame deletion in *Streptomyces* sp. TP-A0867. a) The strategies used in gene in-frame deletion. b) PCR verification of *avm* mutants: Lane1, amplified with Screen-F/R and mutants; Lane 2, amplified with Screen-F/R and WT; Lane M, Trans2K® Plus II DNA marker. These experiments are repeated at least twice with similar results.

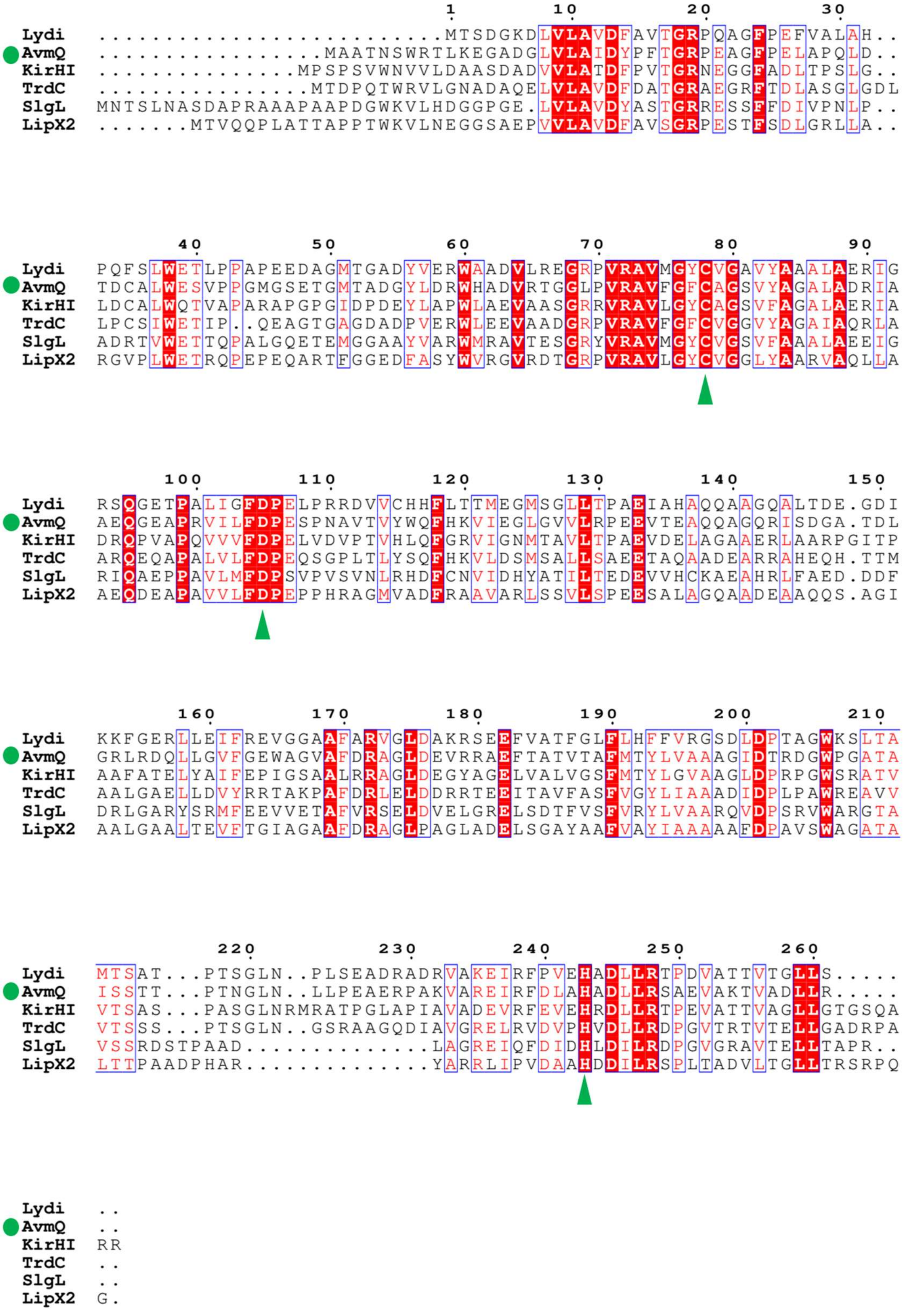

Supplementary Figure 3. Alignment of AvmQ with the reported and possible secondary sequence of Dieckmann cyclase. ▲ designates conserved catalytic triad residues.[6]

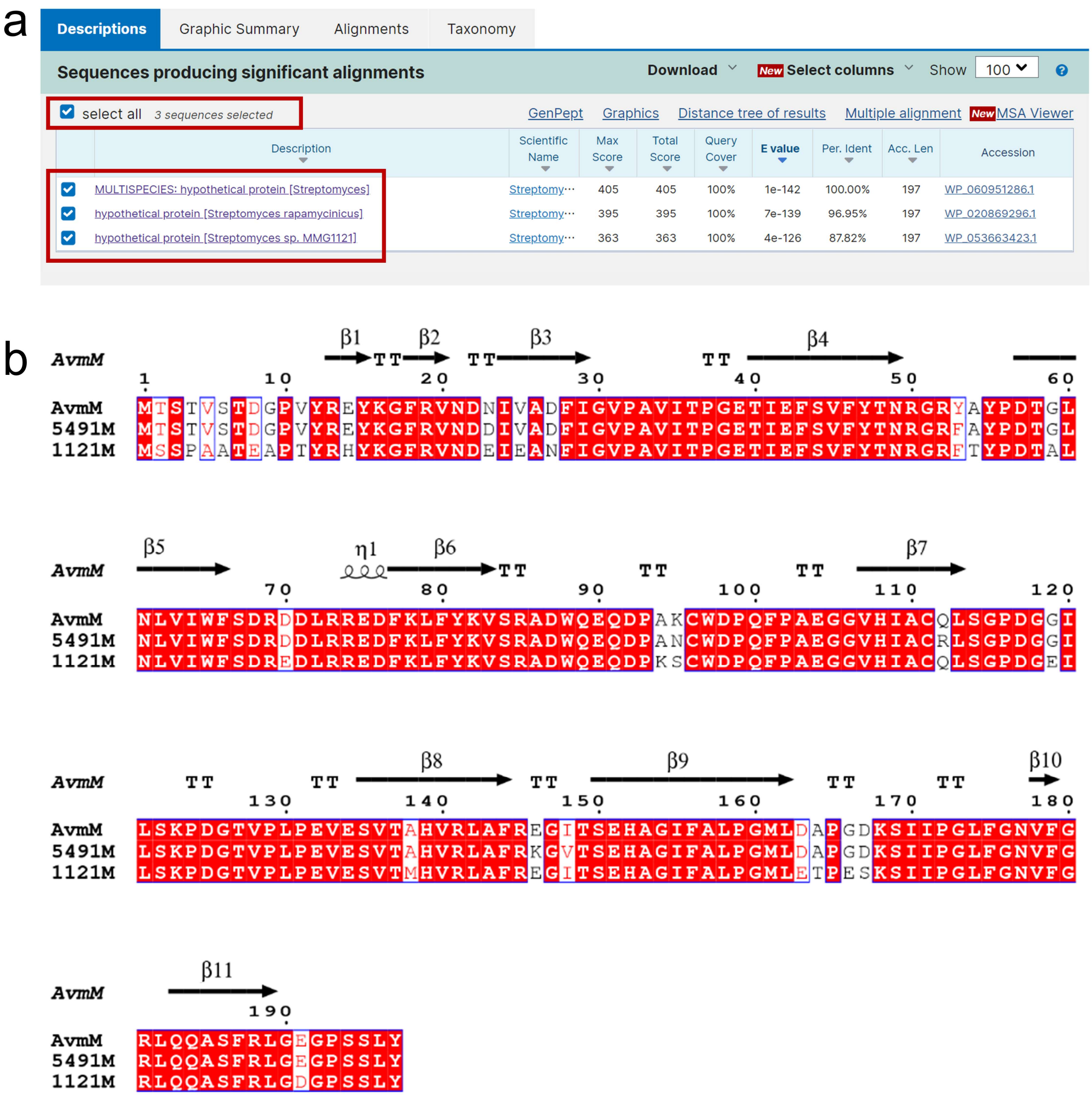

Supplementary Figure 4. Database comparison result of AvmM. a) Blast result of AvmM. b) Sequence alignment of AvmM and two homologous proteins 5491M and 1121M. The secondary structure elements of AvmM are presented at the top. The proteins 5491M (WP_020869296) and 1121M (WP_053663423) are from *S. rapamycinicus* NRRL 5491 and *Streptomyces* sp. MMG1121, respectively.

No.	Template	Alignment Coverage	3D model	Confidence	I.D. %	Template Information
1	c4LBAb	Residues 31-92 (30%)	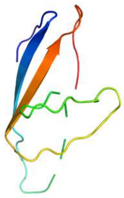	63.6	25	conjugative transposon lipoprotein
2	c3L3Ba	Residues 31-92 (30%)	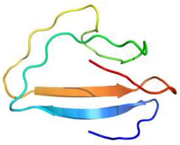	49.3	35	conserved protein found in conjugate transposon
3	c2L7Qa	Residues 31-82 (25%)	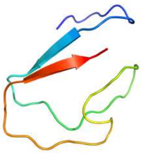	41.5	37	conserved protein found in conjugate transposon
4	d2HTHb1	Residues 120-151 (15%)	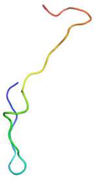	27.8	25	human EAP45/ESCRT-II GLUE domain
5	c4F2Fa	Residues 4-55 (25%)	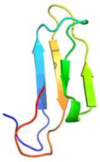	26.2	13	cation-transporting ATPase, e1-e2 family protein
6	c2P9Ra	Residues 33-79 (23%)	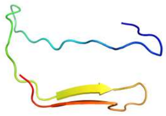	24.3	13	human alpha2-macroglogulin
7	d2J07a2	Residues 61-105 (21%)	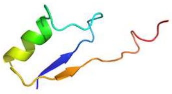	22.2	22	cryptochrome/photolyase, N-terminal domain
8	c4F2Ea	Residues 20-55 (17%)	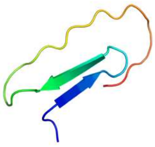	20.6	25	d39 copper chaperone2 cupa with cu(i)

9	c3J0If	Residues 128-197 (35%)	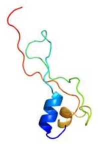	20.0	36	*pseudomonas* virus phiKZ tail sheath protein fold
10	d1W8Oa1	Residues 36-62 (15%)	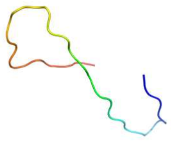	16.6	29	immunoglobulin-like beta-sandwich

Supplementary Figure 5. | The top 10 results given in the Phyre^{2} protein structure comparison.[7] The underlined four letters indicated the relative PDB number.

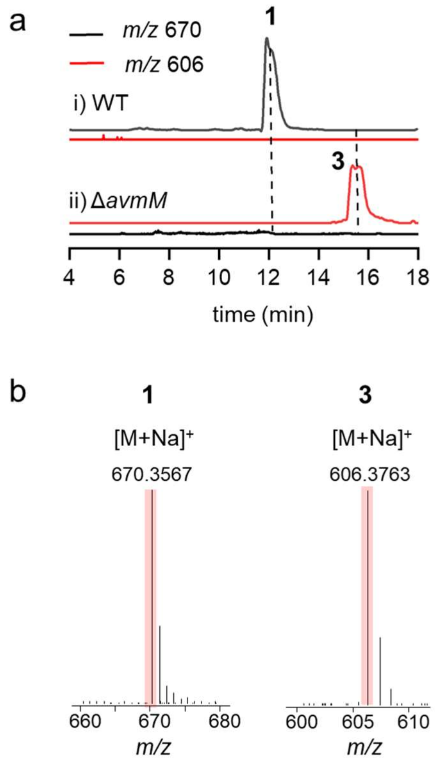

Supplementary Figure 6. HPLC/MS analysis of metabolic extracts from *S*. sp. TP-A0867 wild-type and *avmM* mutant strain.

```
AvmA-KR            ......HAAGVLDDAVVTALTPERLHAVLRPKVDAAWHLHELTAGRDLAAFVLFSSVVGTLGGAGQGNYAAANTFLDALAHH
AvmB-KR            ......HAAGAAEMPDVR-TDLGDYAAVVSAKVAGALHLDELFADEPLDAFVLFSSIAAVWGSGGQGAYAAANAFLDALAEE
AvmC-KR1           ......HTAGVGQLTPLLDTTPGECAEVLGAKAAGAALLDALLPPERLDAFVLFSSNAGVWGSGNQGAYAAANAYLDALAQR
AvmC-KR2           ......HAAGVLDDAVVESLTPERLGTVLRPKVDAAWHLHELTREMDLSAFVLFSSASGTLGAPGQANYAAANSCLDALAQH
AvmD-KR1           ......HAAGVLDDAVVESLTPERLEGVLRPKVDAAWHLHELTREMDLSAFVLFSSAAGTLGGPGQANYAAANSCLDALAEF
AvmD-KR2           ......HAAGVLDDAVVESLTPERLEGVLRPKVDAAWHLHELTREMDLSAFVLFSSASGTLGGPGQANYAAANSYLDALAQH
AvmD-KR3           ......HAAGVLDDGVVGALTPERLDAVLRPKVDAAWHLHELTREMDLSAFVLFSSASGTLGGPGQANYAAANAFLDALAGQ
AvmD-KR4           ......HAAGVVEDGVLQTLEPGQLDRVLRPKVDAALNLHALTEGLPLSRFVLFSSAVATMGAAGQANYAAANAFLDALAQR
AvmE-KR1           ......HAAGVLDDAVVESLTPERLGTVLRPKVDAAWYLHELTREMDLSAFVLFSSASGTLGGPGQANYAAANSYLDALAQH
AvmE-KR2           ......HAAGVLDDAVVESLTPERLGTVLRPKVDAAWHLHELTREMDLSAFVLFSSASGTLGAPGQANYAAANSYLDALAQH
AvmF-KR1           ......HAAGVLDDGVVGALTPERLDAVLRPKVDAAWHLHELTRGMDLSAFVLFSSASGTLGGPGQANYAAANAFLDALAGQ
AvmF-KR2           ......HTEGVVEDCPVVGLSGGRYDELVSGRLAGLTHLYELAEDLELSAFVVFSSITGTVGGVGQAARAMTDAYADALAGR
AmpKR2(A1type)     ......SAGVAHDDAPVADLTLGQLDALMRAKLTAARHLHELTADLDLDAFVLFSSGAAVWGSGGQPGYAAANAYLDALAEH
AmpKR11(A2type)    ......HTAATIELHTLDATTLDDFDRVLAAKVTGAQILDELLDDEELDDFVLYSSTAGMWGSGAHAAYVAGNAYLAALAEH
SpnKR2(B1type)     ......HAAGVLDDGVSESLTVERLDQVLRPKVDGARNLLELIDPD--VALVLFSSVSGVLGSGGQGNYAAANSFLDALAQQ
EryKR1(B2type)     ......HAAATLDDGTVDTLTGERIERASRAKVLGARNLHELTRELDLTAFVLFSSFASAFGAPGLGGYAPGNAYLDGLAQQ
```

Supplementary Figure 7. Analysis of catalytic residues of *avm* KR domains of PKS. The yellow shades indicate four essential catalytic residues; the green or red shades indicate the conserved motif for A type or B type KR domians (AmpKR2,[8] AmpKR3,[9] SpnKR2,[10] EryKR1[11]), reseptivily. Moreover, the residues seperate the A1/A2 or B1/B2 type KR domains are colored as cyan.

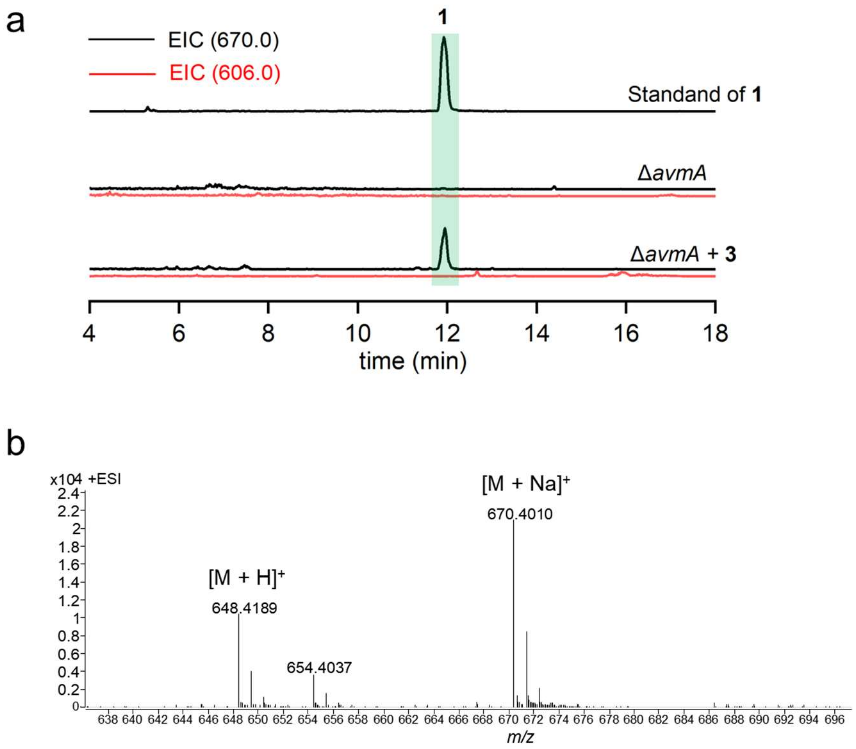

Supplementary Figure 8. LC-MS analysis of chemical complementation of compound **3** into the Δ*avmA* mutant strain. The experiments were independently repeated three times with similar results and the overall turn-over rate of **3** to **1** is about 20% (production of **1** is ~100 μg for feeding 500 μg **3**).

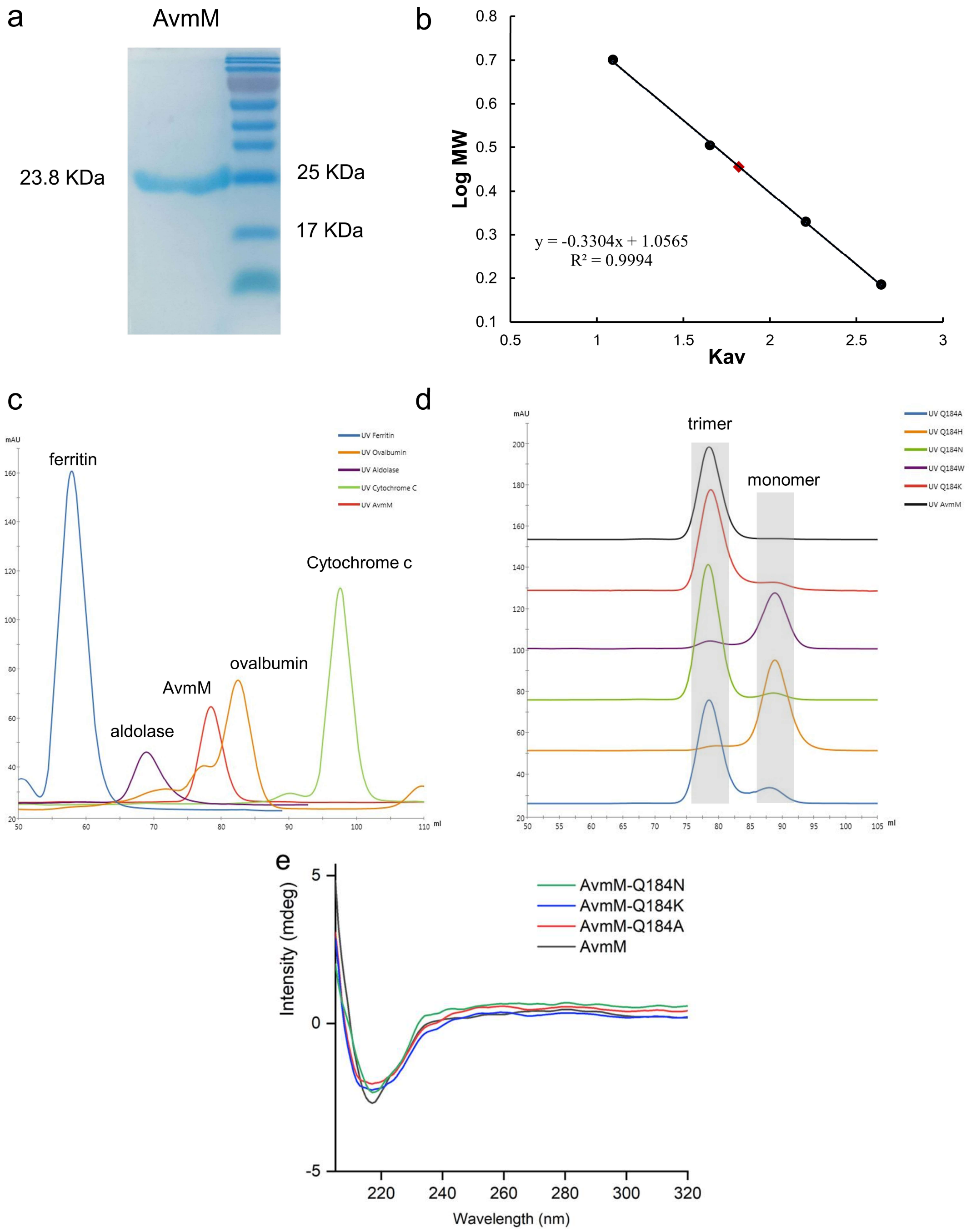

Supplementary Figure 9. SDS page and size-exclusion chromatography (SEC) of the purified proteins. **a,** AvmM (Molecular weight, 23.8 kDa); **b,** Size-exclusion chromatography of AvmM. Cytochrome c (12.4 kDa), ovalbumin (44 kDa), aldolase (160 kDa), and ferritin (440 kDa) were used to construct the standard curve (black circles). The AvmM (red dot) eluted at retention volumes of 78.6 mL, correlating to molecular weights (MWs) of 66.2 kDa. The calculated MW for AvmM is ~23.8 kDa. Thus, AvmM formed a homotrimer in solution. The experiments were repeated twice with similar results (a representative example is shown); **c,** SEC of AvmM together with standards generated by UNICORN 7. **d,** SEC analysis of wild type AvmM together with other mutants. e, the CD analysis of WT with Q184 mutant of AvmM. The experiments were independently repeated twice with similar results.

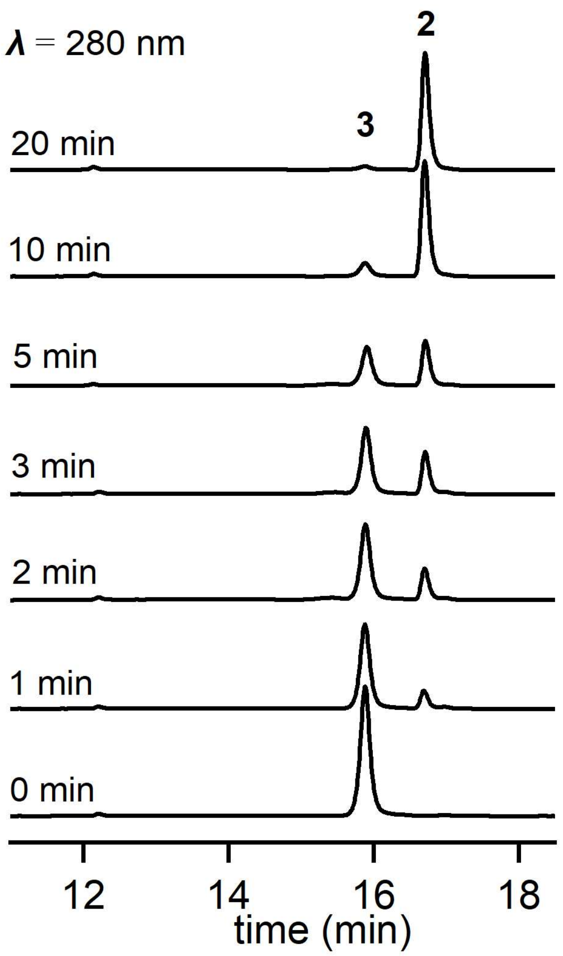

Supplementary Figure 10. Time-course analysis of AvmM catalyzed reaction at room temperature.

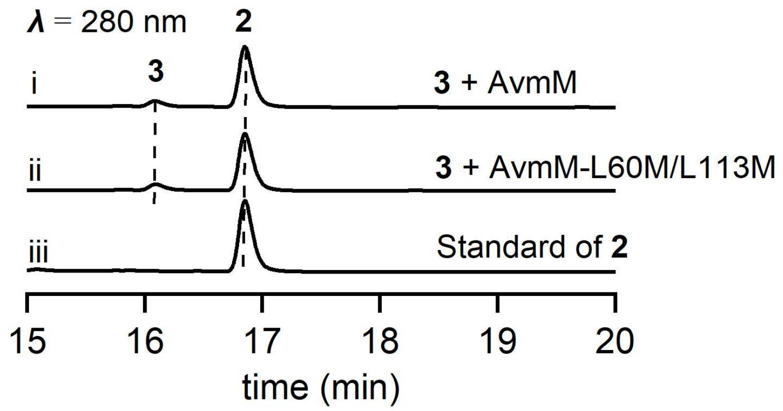

Supplementary Figure 11. In vitro assays of AvmM and AvmM-L60M/L113M.

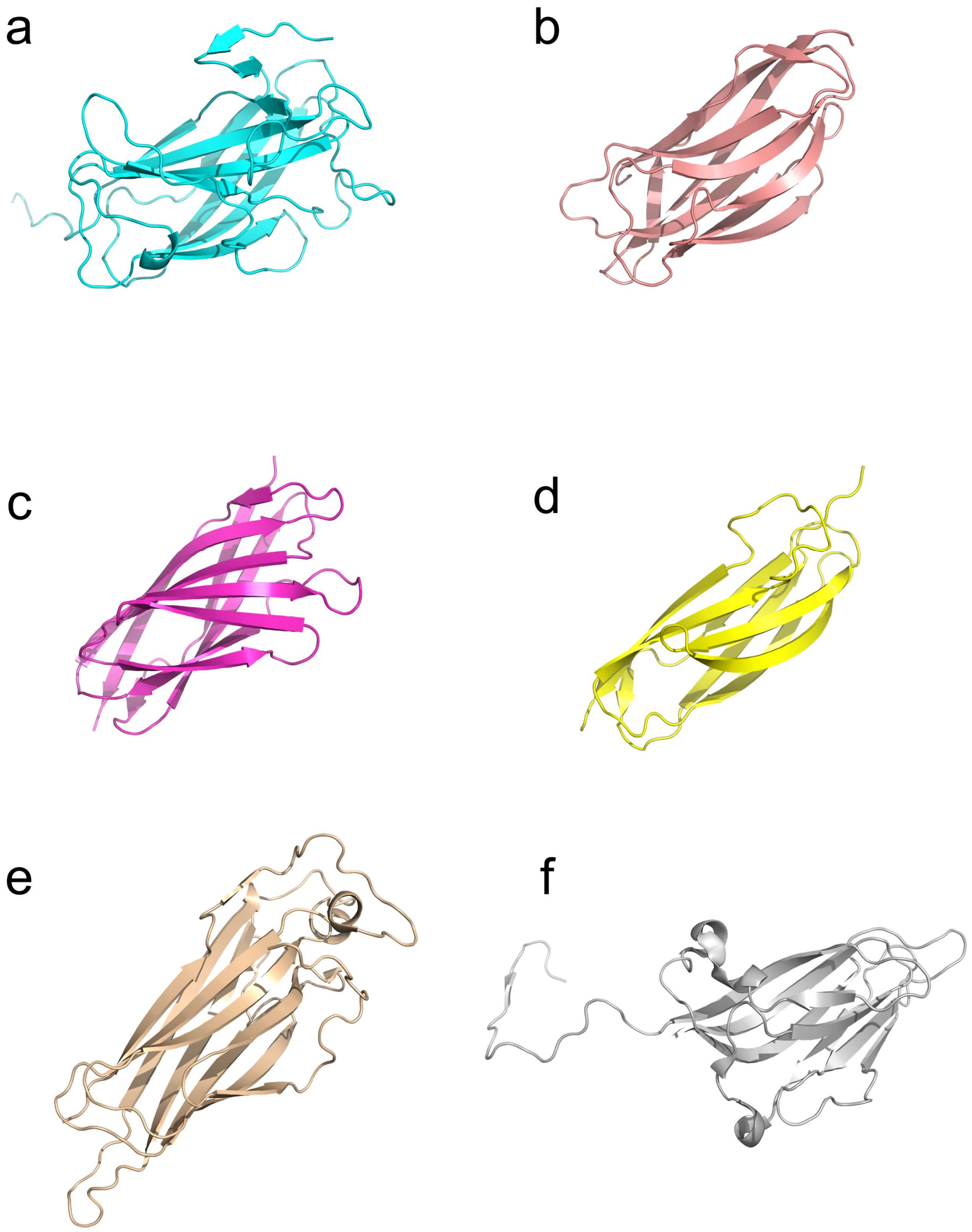

Supplementary Figure 12. The crystal structure of AvmM monomer with other similar proteins searched by Dali sever.[12] **a,** AvmM. **b,** Cohesin module (2VO8). **c,** Cohesin module (2JH2). **d,** CARDB domain of PF1109 from *Pyrococcus furiosus* (2KL6). **e,** Part of Human alpha-V beta-3 Integrin (6AVU). **f,** Classical jelly roll fold structure from *Satellite Tobacco* Mosaic Virus (4OQ9).

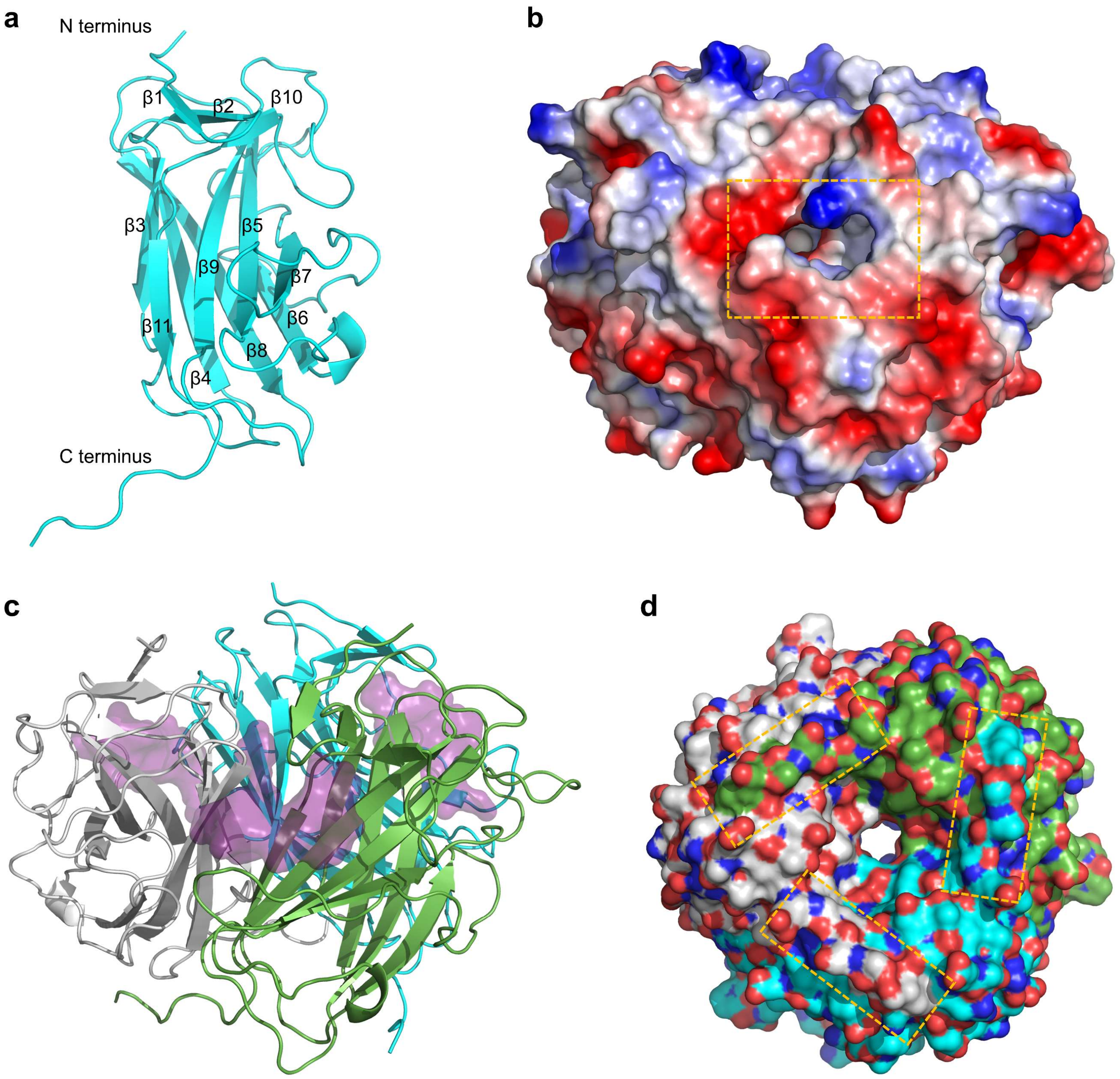

Supplementary Figure 13. The crystal structure of AvmM. **a,** Cartoon representations of the monomeric AvmM. **b,** Electrostatic surface potential of AvmM. Positive surface charge is colored in blue; negative surface charge is colored red; and neutral surface is in white. The dotted square indicated the catalytic cave. **c,** Cartoon representation of apo-AvmM tertiary structure and binding cavity (magenta). The volume of the cavity was calculated using POCASA software.[13] **d,** the surface presentation of trimeric AvmM and the orange dotted square highlight the interaction between C terminus loops with other chains. The Chain A is shown in green, Chain B is shown in white and Chain C is shown in cyan.

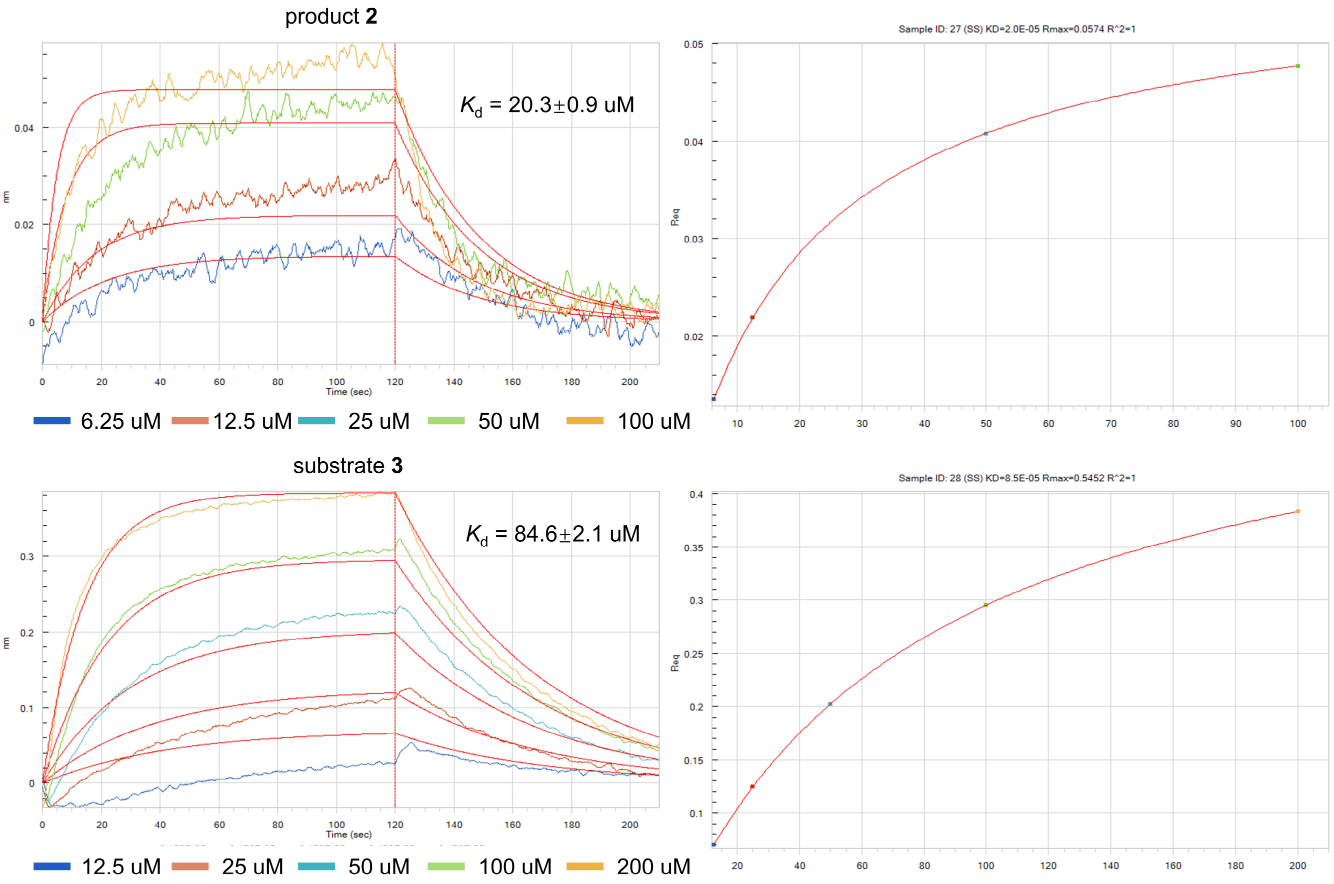

Supplementary Figure 14. Analysis of the interaction between AvmM and product **2** (top panels) or substrate **3** (lower panels) by biolayer interferometry. The fitting view is shown on the left and the steady state analysis on the right.

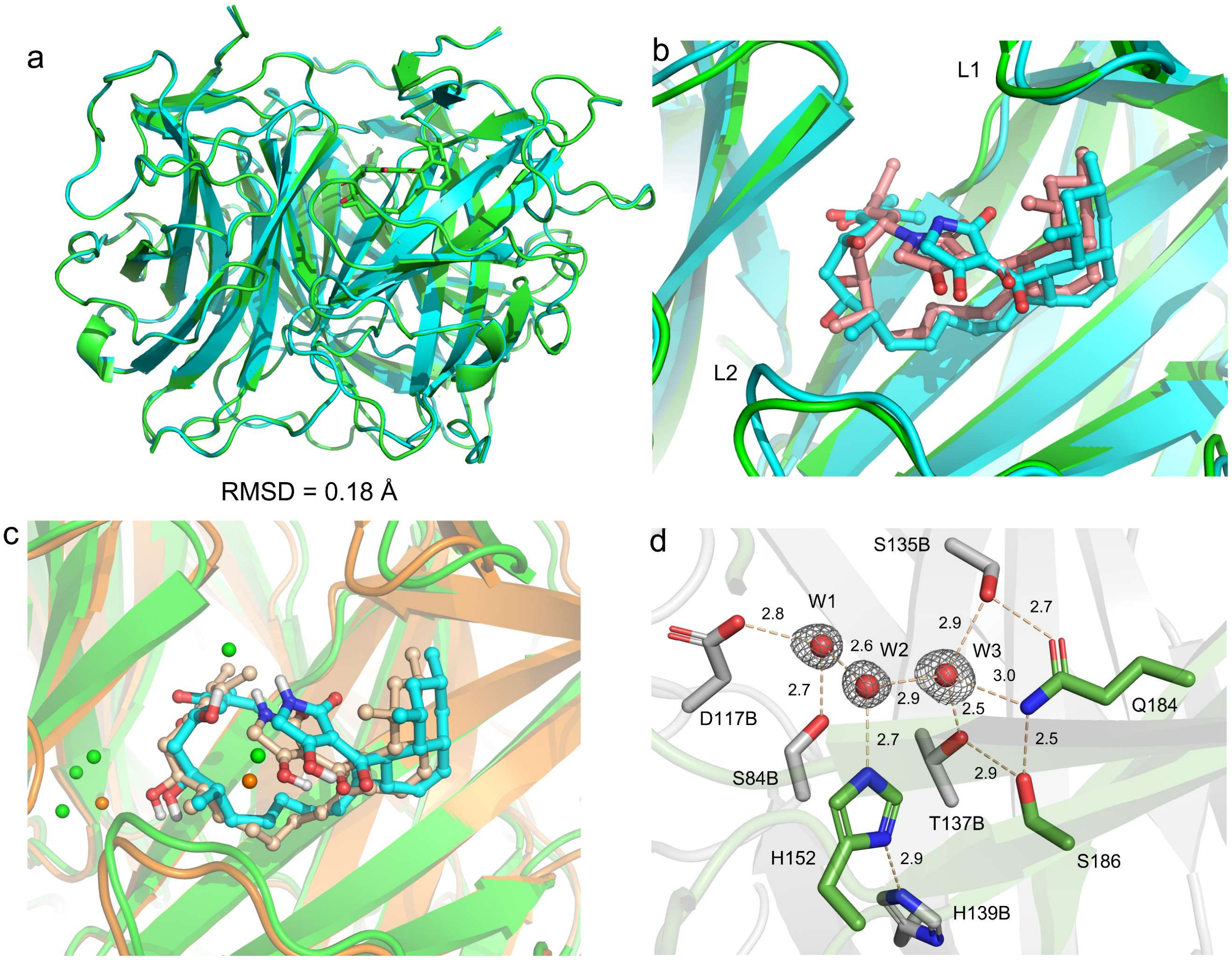

Supplementary Figure 15. Comparison of docking and complex results. **a,** Comparison of AvmM and AvmM-**2** complex. Aligning two structures revealed no significant changes upon substrate binding. Cyan = AvmM. Green = AvmM-**2** complex. **b,** Overlay of AvmM structures docking with **3** and in complex with **2**. The docking result is presented in cyan while the complex structure is in green. **c,** Comparison of docking of **3** and **4** in AvmM and shows similar conformation, compound **3** presents as cyan while compound **4** presents as wheat. The dots presents the water molecules, while waters in **3** is colored as green and water in **4** is colored as orange. **d,** Hydrogen network to activate Gln184. The 2*Fo-Fc* map is shown in thin grey mesh and contoured at 1.0 σ.

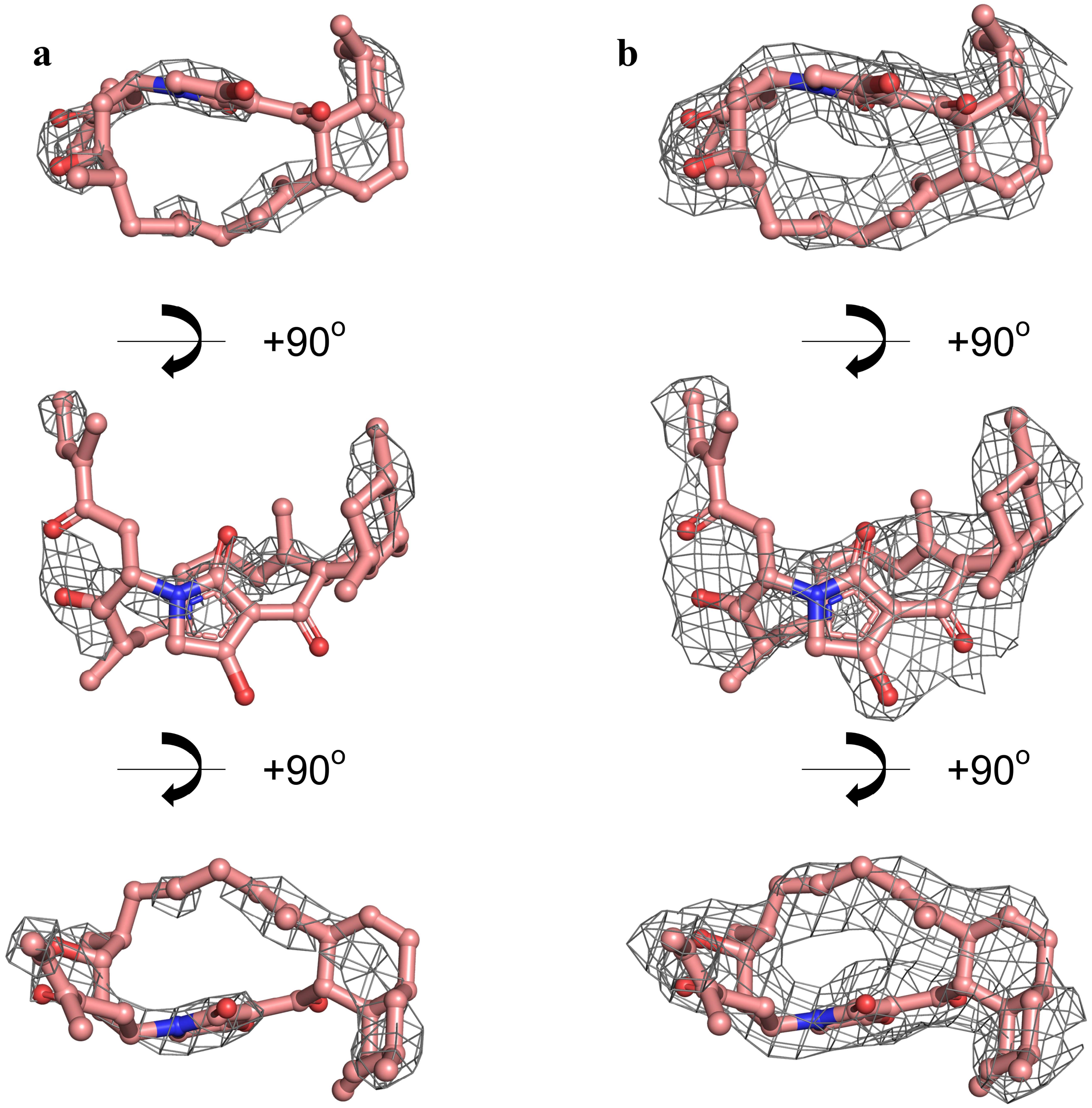

Supplementary Figure 16. Electron density of the **2** in the AvmM co-crystal structure. **a,** The 2*Fo*–*Fc* map contoured at 1.0 σ represented as thin gray mesh. **b,** The polder omit map is shown in thin grey mesh and contoured at 4.0 σ.

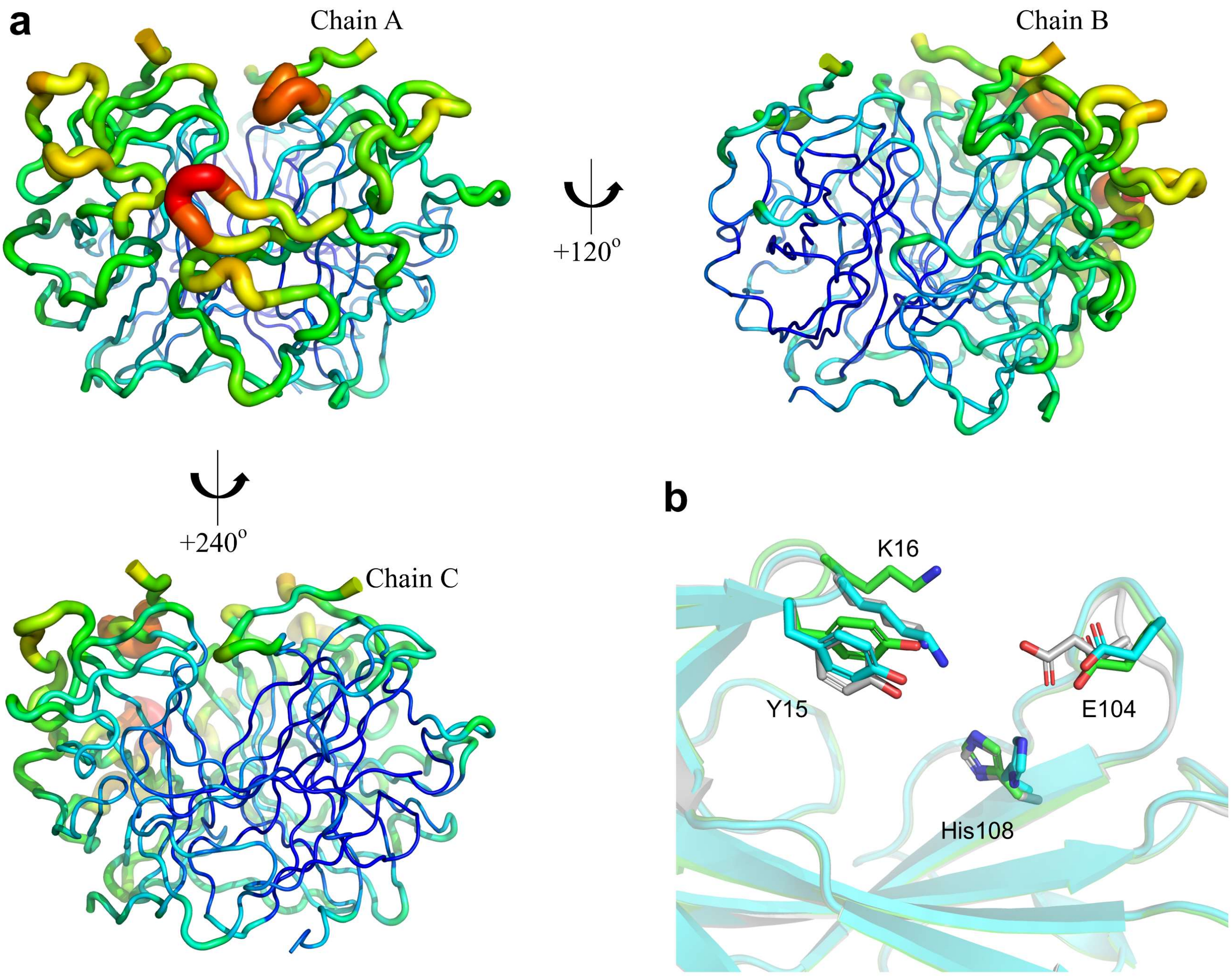

Supplementary Figure 17. Flexibility analysis of AvmM. **a,** B-factor diagram of AvmM represented by the B-factor putty program in PyMOL. The B-factor values are illustrated by color, ranging from low (blue) to high (red). **b,** Four representative residues are shown to emphasize the flexibility, which is observed in the solved structures.

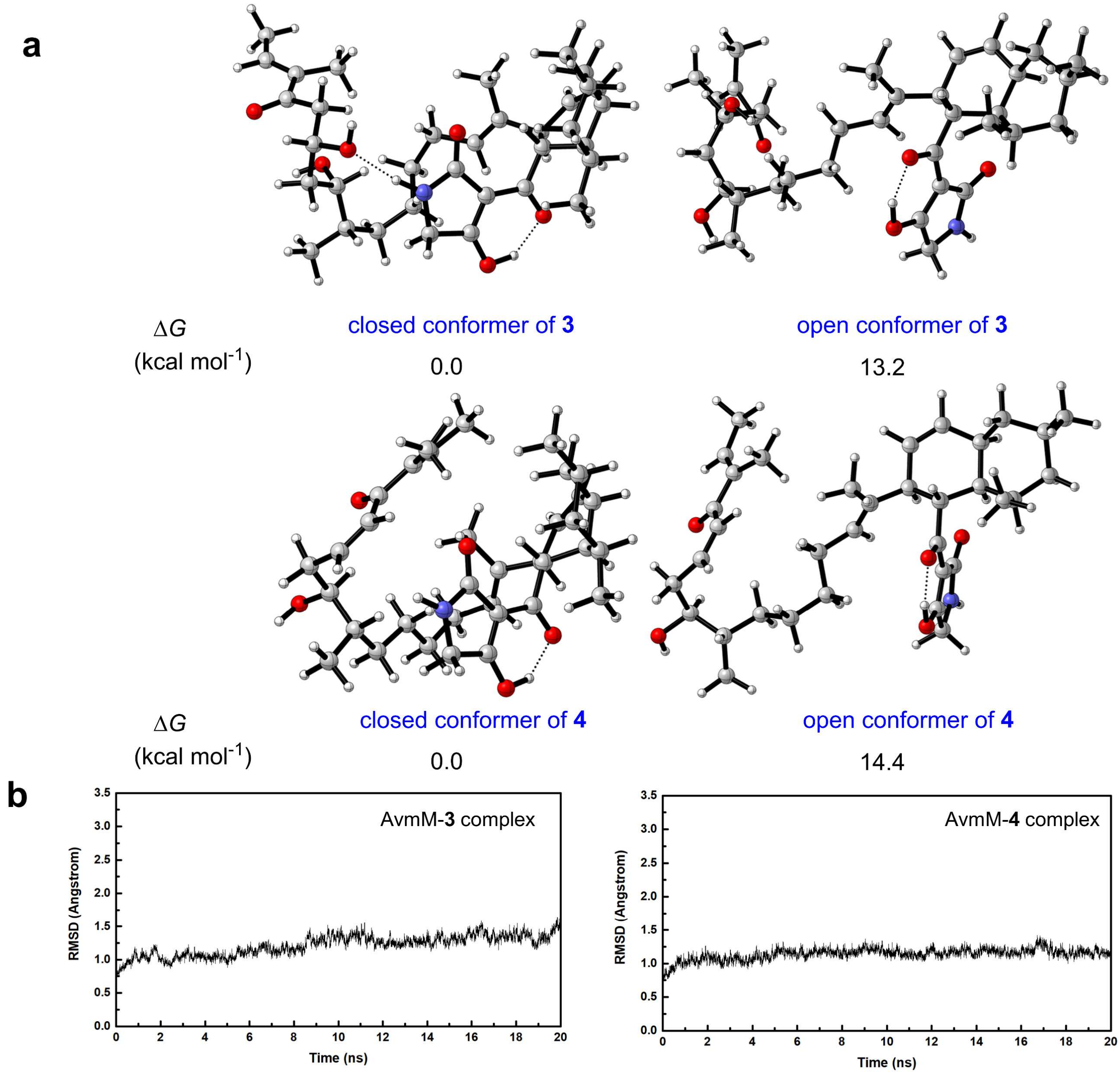

Supplementary Figure 18. DFT and MD and analysis of AvmM and substrates **3** and **4**. a, The computed Gibbs free energies (in kcal mol^{-1}) of two conformers of compound **3** and **4**, computed at the CPCM(water)-B3LYP-D3/6-311++G(2d,p)//CPCM(water)-B3LYP-D3/6-31+G(d) level of theory (carbon: gray, hydrogen: white, oxygen: red, nitrogen: blue); b, RMSD of backbone heavy atoms of AvmM relative to the first snapshot during 20 ns classical MD simulation on AvmM-**3** complex (left) and AvmM-**4** complex (right).

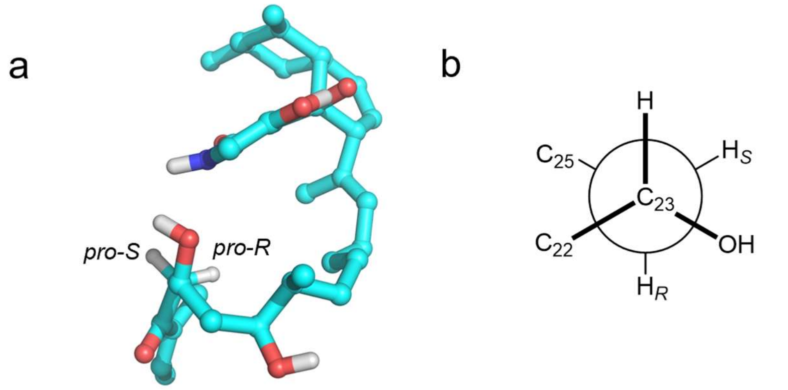

Supplementary Figure 19. Proposed mechanism of dehydration step catalyzed by AvmM. **a.** Close up view of the crystal structure of AvmM active site docked with substrate **3** while all other amino acids are omitted for clarity. The two hydrogen atoms of C24 are labeled as *R* for pro-*R* and S for pro-*S*. **b.** Rotamer for C23-C25 segment of docked **3** in the active sites. A syn-elimination of the water was proposed to generate the *Z* type double bond.

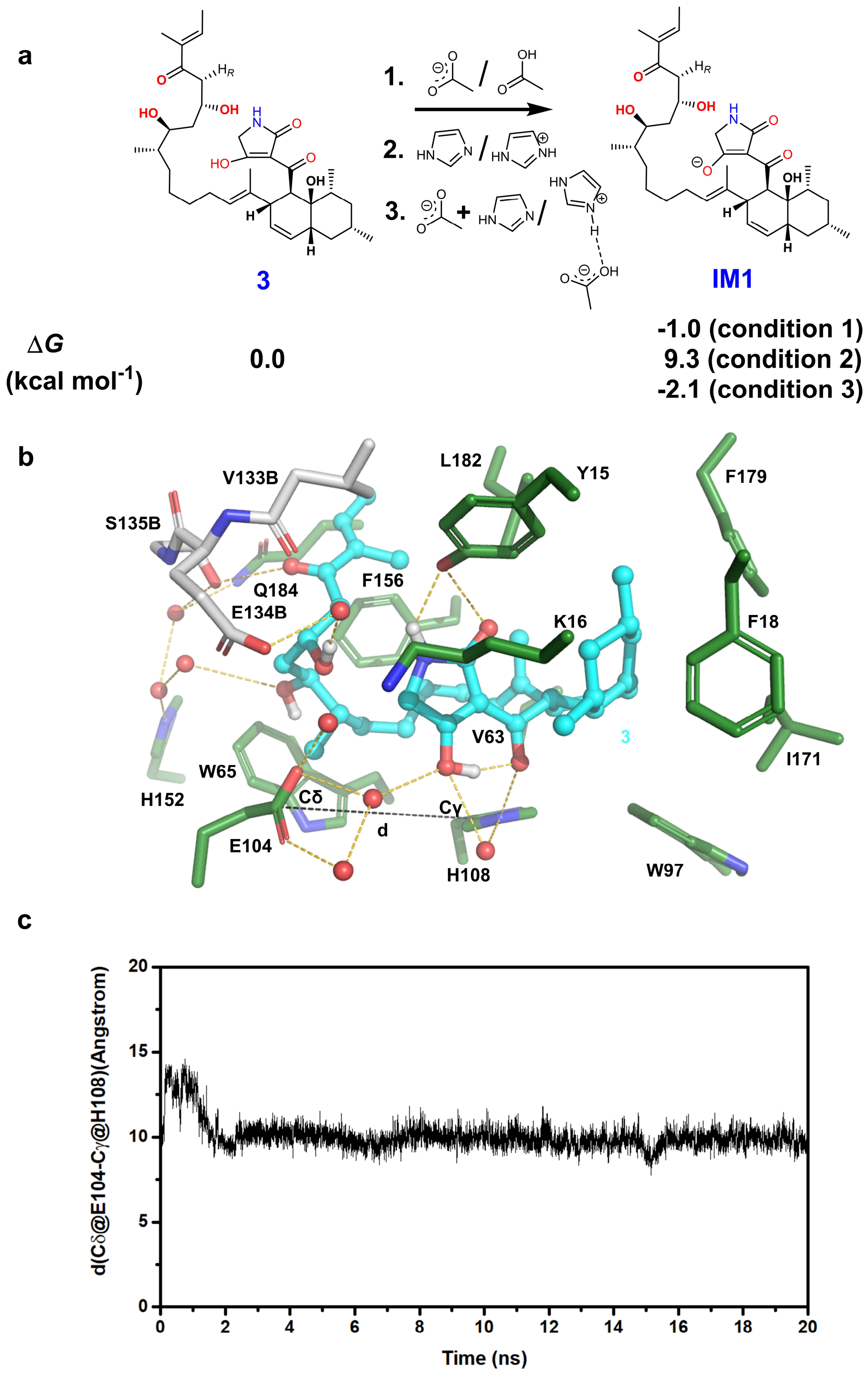

Supplementary Figure 20. a,The computed Gibbs free energies (in kcal mol^{-1}) of **3** reacted with H108 (imidazole) or/and E104 (acetate ion) at the CPCM(water)-B3LYP-D3/6-311++G(2d,p)//CPCM(water)-B3LYP-D3/6-31+G(d) level of theory. **b**, Close-up view of MD representative snapshots of AvmM active site complexed with substrate **3**. Water molecules are shown in red spheres. Hydrogen bonds are in gold dash lines and the distance between the side chains of E104 and H108 is in black dash line. **c**, Distance between the side chains carbon atoms Cδ and Cγ of E104 and H108 during 20 ns classical MD simulation on AvmM-**3** complex.

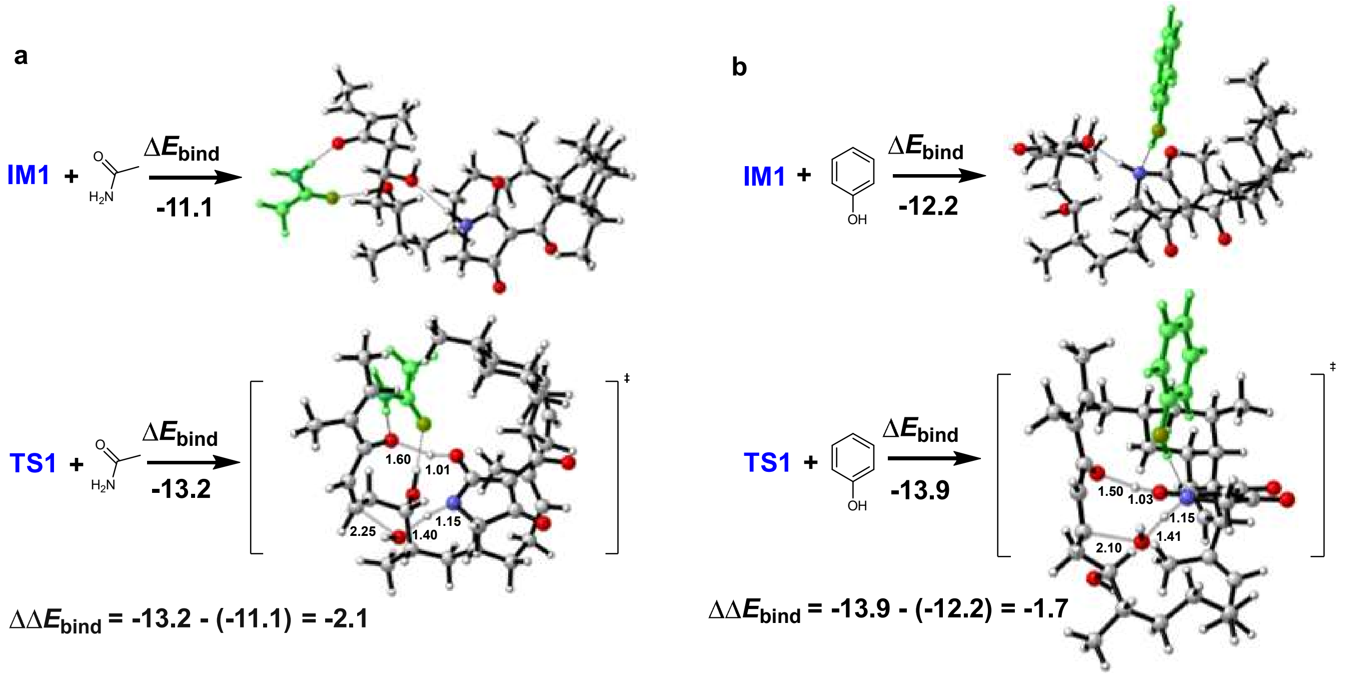

Supplementary Figure 21. The computed energies (in kcal mol^{-1}) of **IM1** and **TS1** stablized by Q184 (acetamide) or Y15 (phenol) at the CPCM(water)-B3LYP-D3/6-311++G(2d,p)//CPCM(water)-B3LYP-D3/6-31+G(d) level of theory (carbon: gray, hydrogen: white, oxygen: red, nitrogen: blue, and distances are shown in Å).

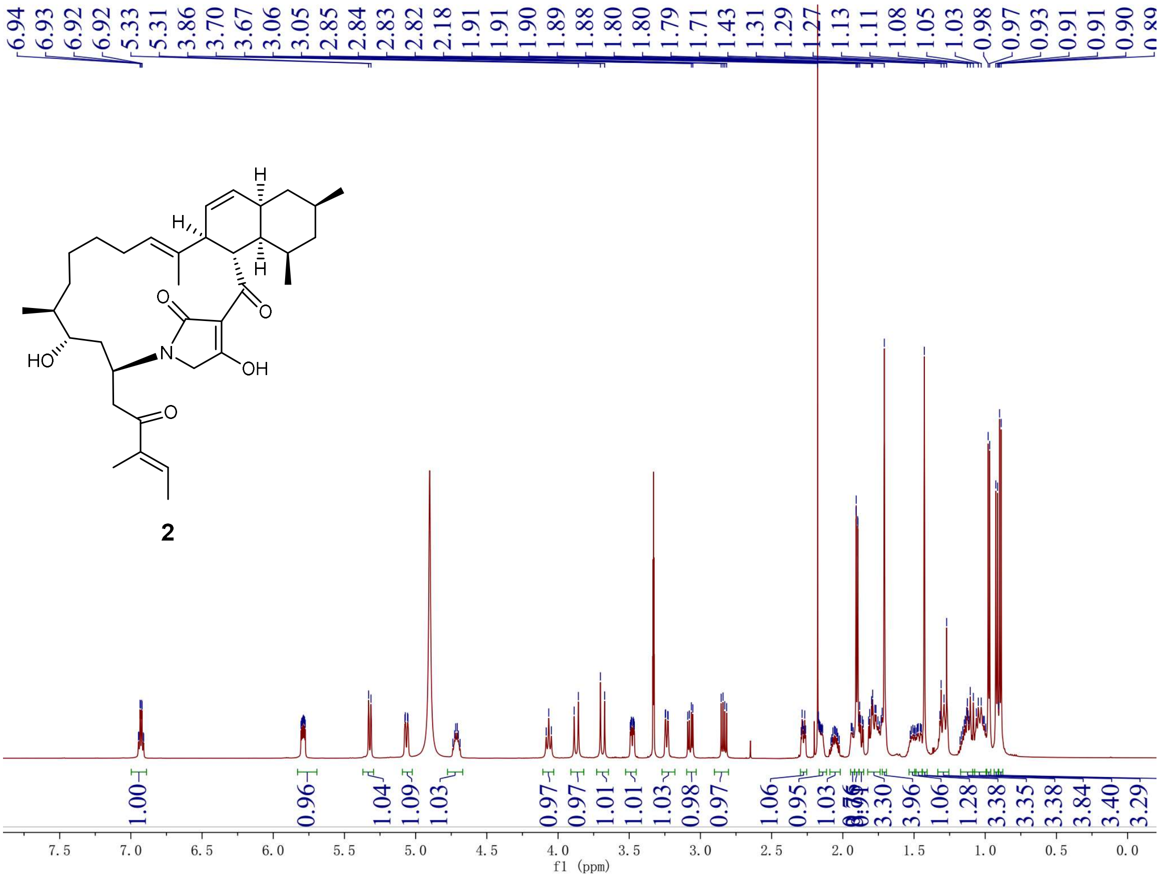

Supplementary Figure 22. ^{1}H NMR spectrum of **2** in methanol-d_4 (600 MHz).

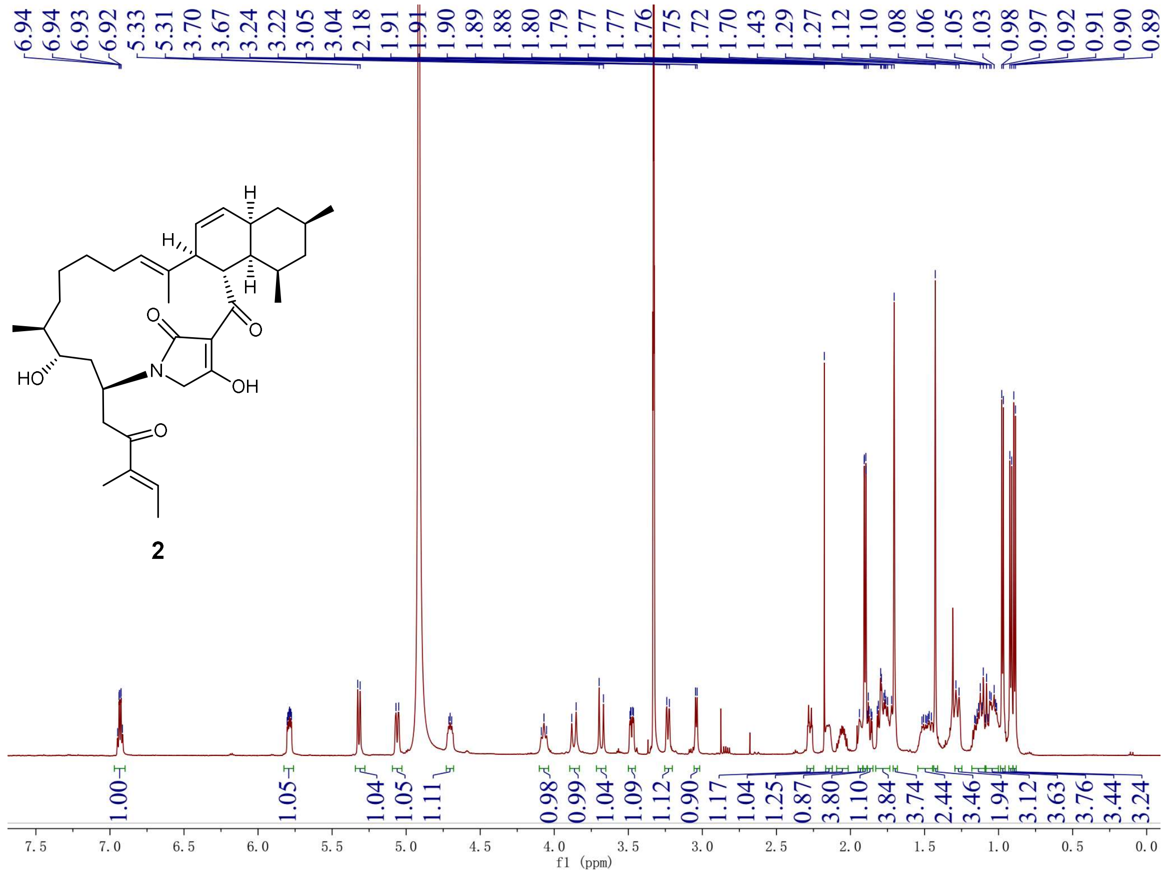

Supplementary Figure 23. ^{1}H NMR spectrum of ^{2}H-**2** in methanol-d_4 (600 MHz).

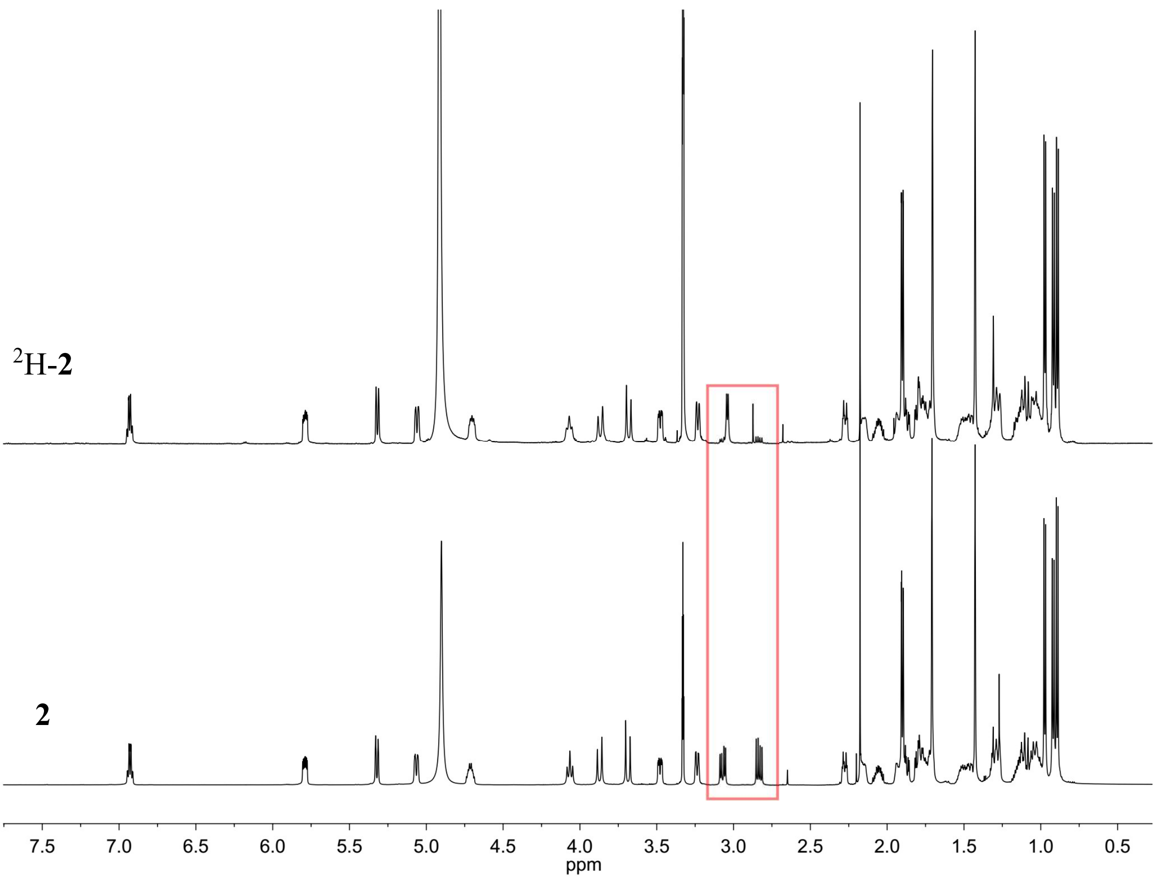

Supplementary Figure 24. Comparison of ^{1}H NMR spectra of **2** and ^{2}H-**2** in methanol-d_4 (600 MHz).

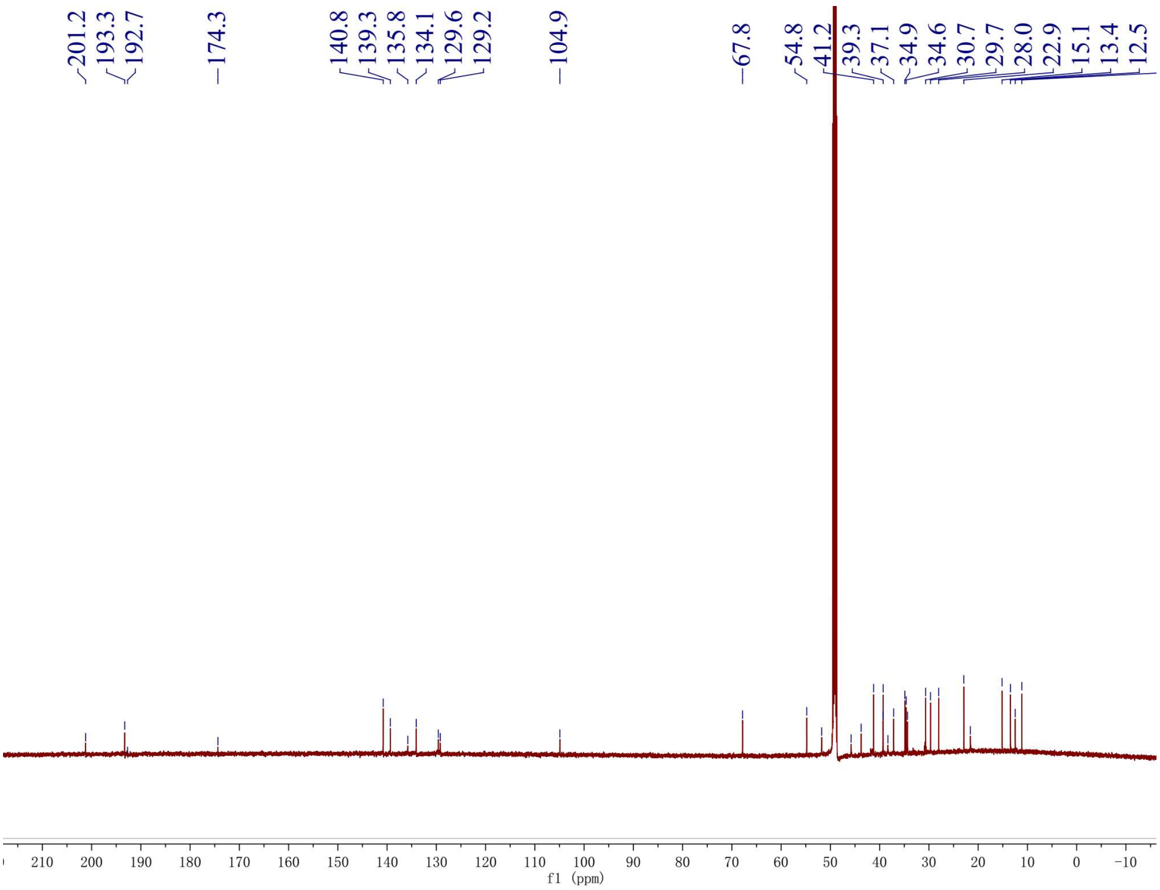

Supplementary Figure 25. ^{13}C NMR spectrum of ^{2}H-**2** in methanol-d_4 (150 MHz).

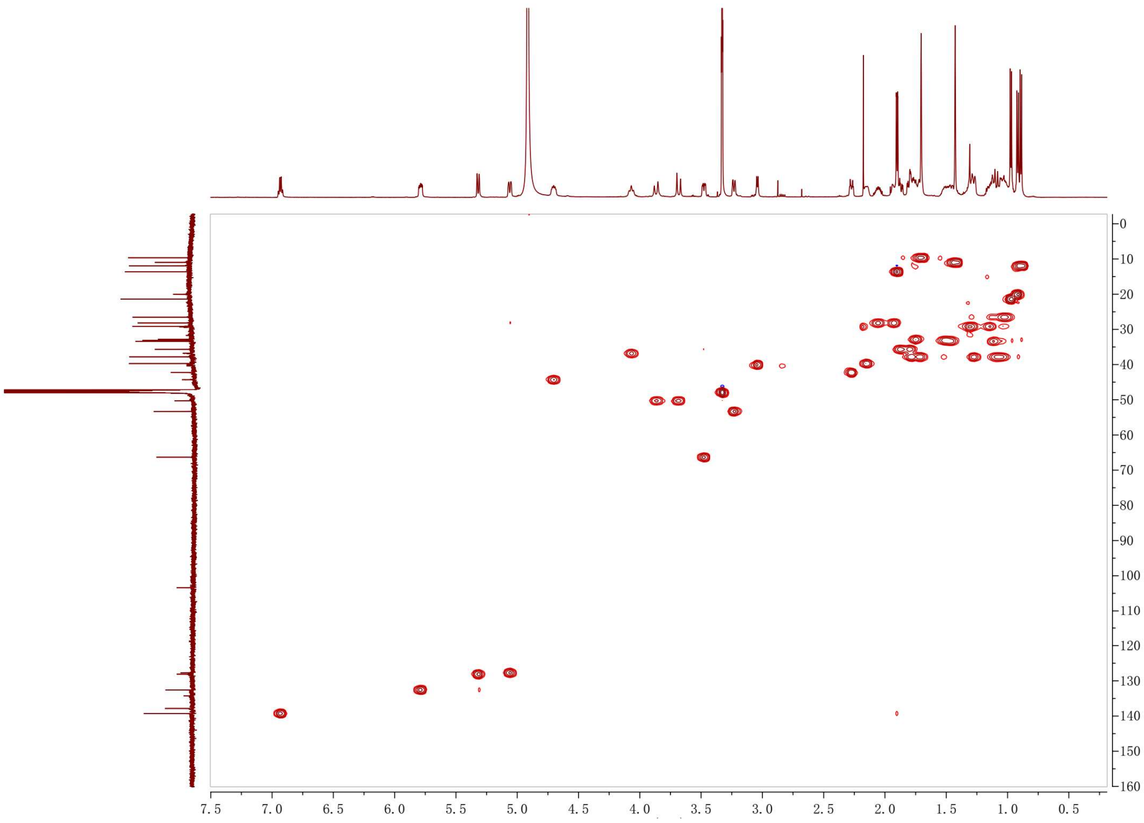

Supplementary Figure 26. HSQC NMR spectrum of ^{2}H-**2** in methanol-d_4.

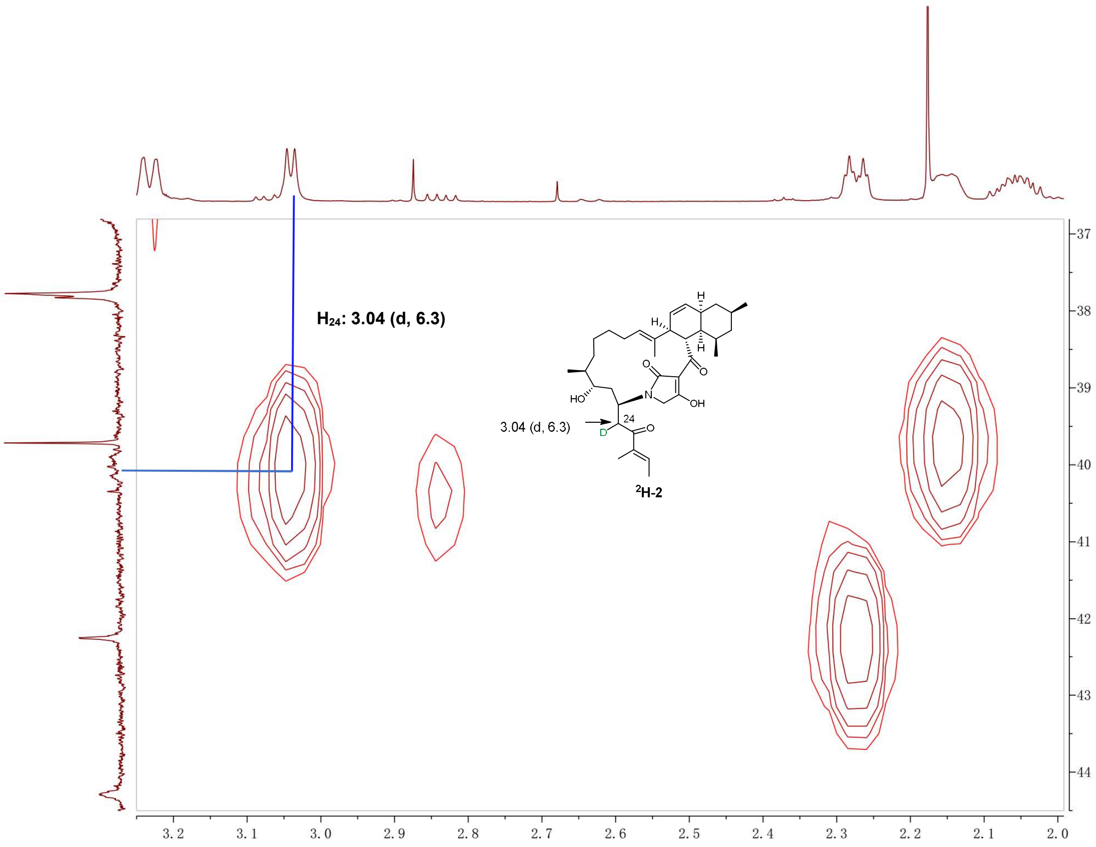

Supplementary Figure 27. Partially amplified HSQC NMR spectrum of ^{2}H-**2** in methanol-d_4.

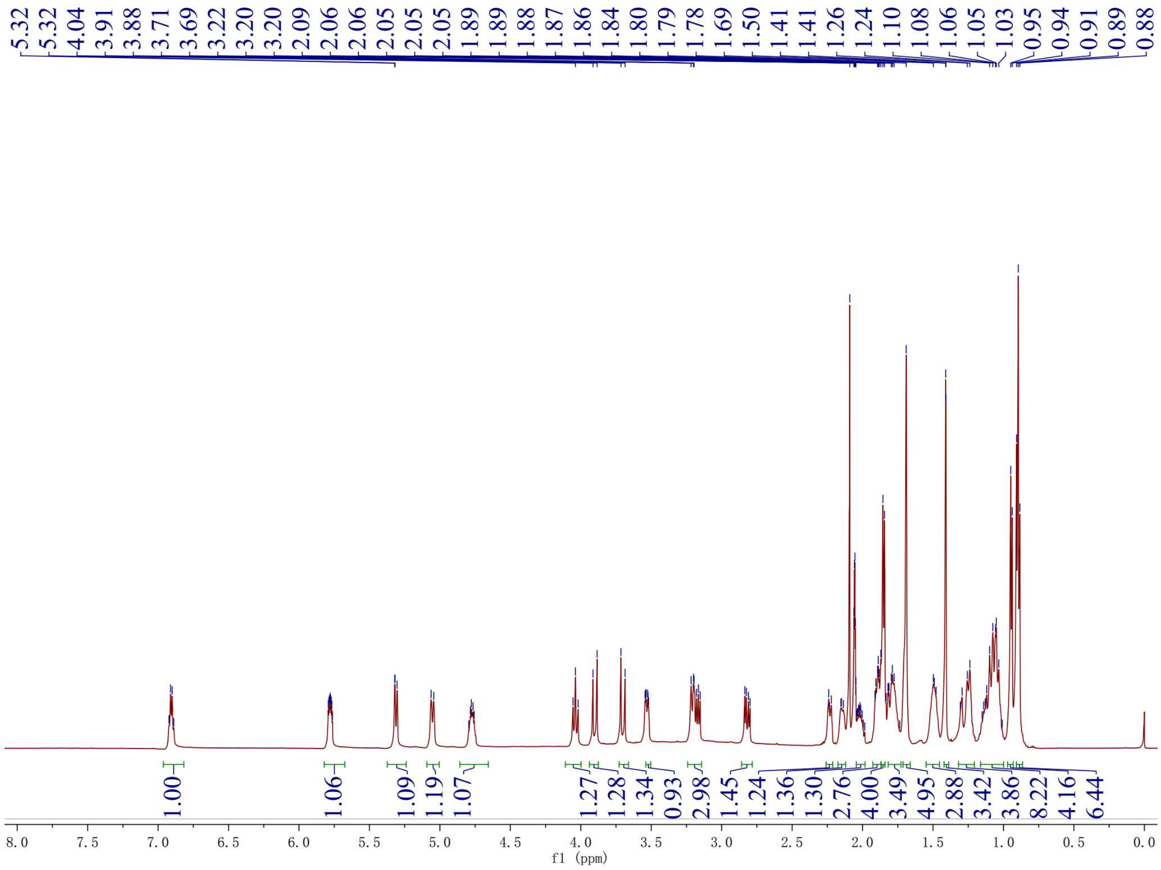

Supplementary Figure 28. ^{1}H NMR spectrum of **2** in acetone-d_6 (600 MHz).

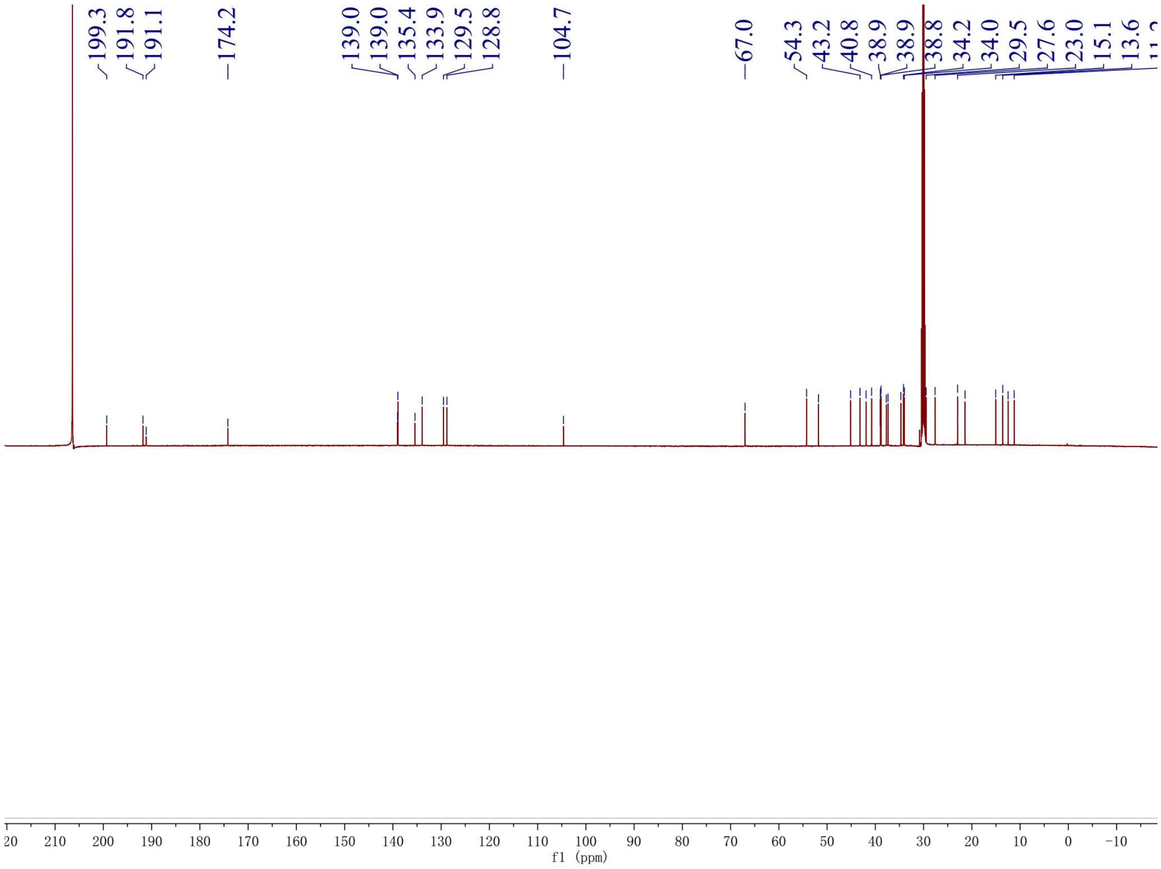

Supplementary Figure 29. ^{13}C NMR spectrum of **2** in acetone-d_6 (150 MHz).

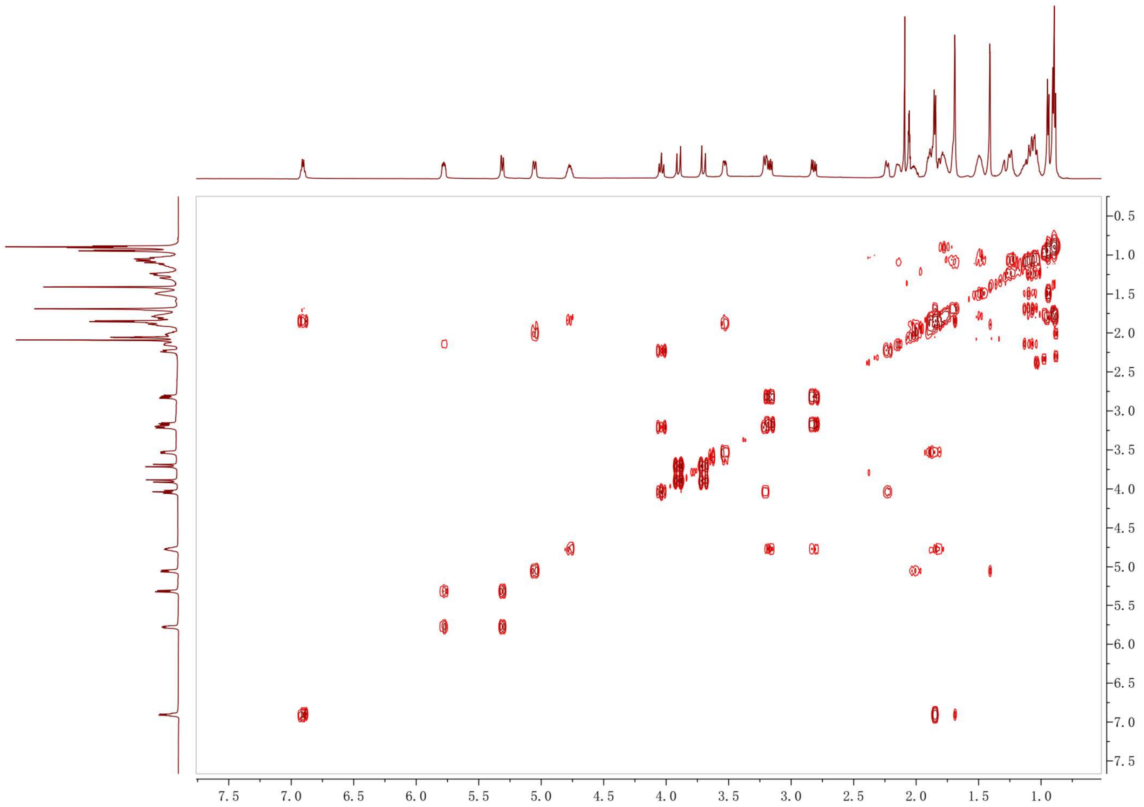

Supplementary Figure 30. ^{1}H-^{1}H COSY NMR spectrum of **2** in acetone-d_6.

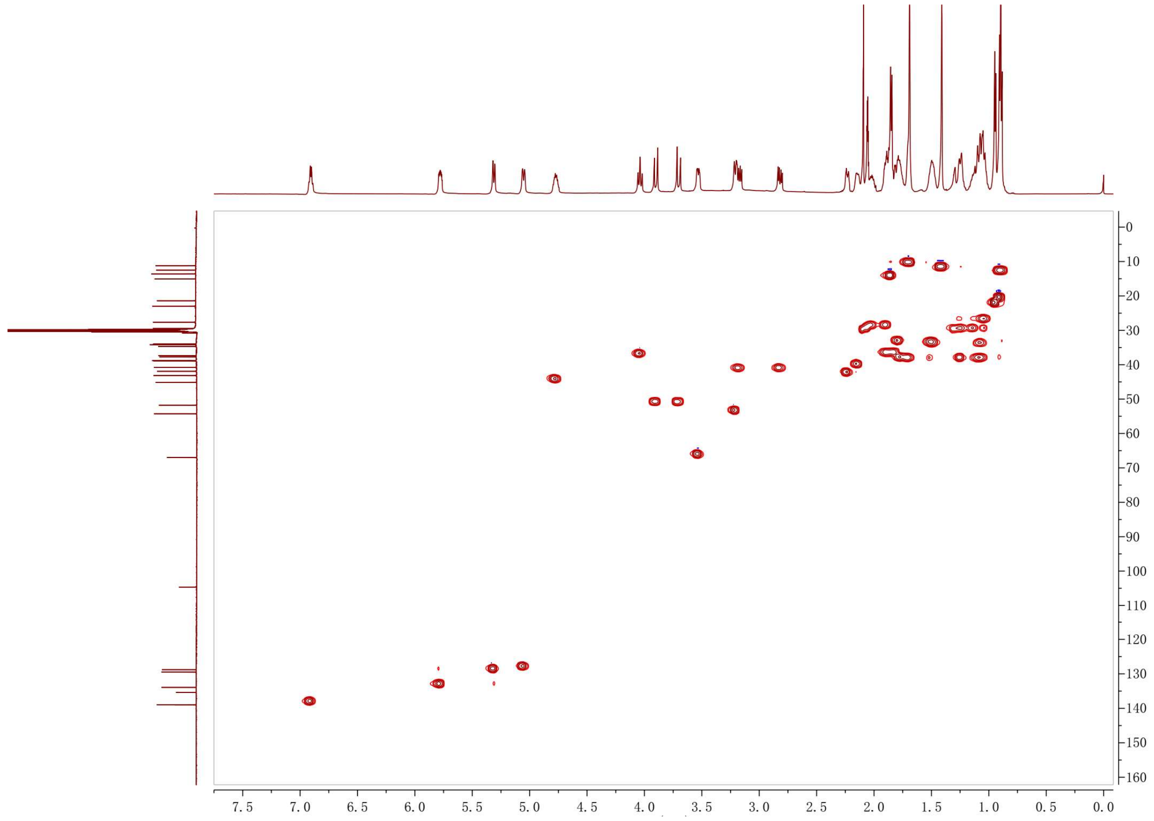

Supplementary Figure 31. HSQC NMR spectrum of **2** in acetone-d_6.

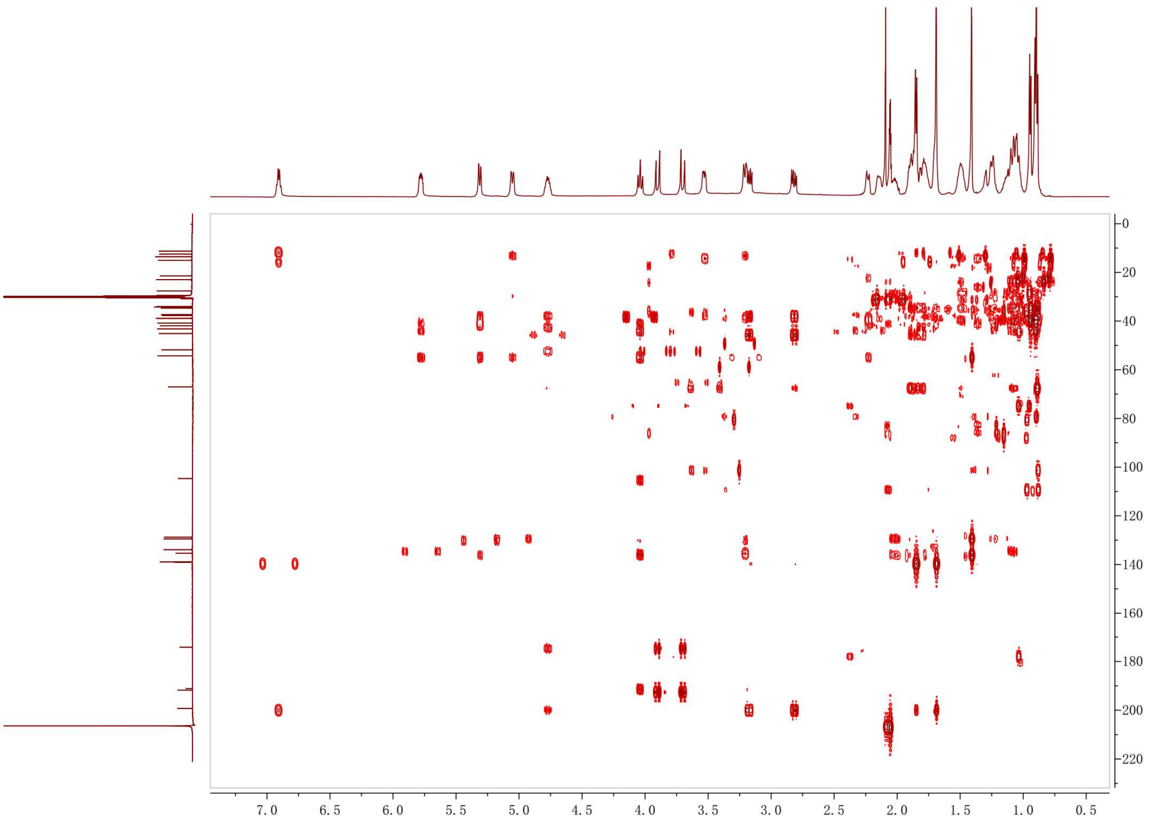

Supplementary Figure 32. HMBC NMR spectrum of **2** in acetone-d_6.

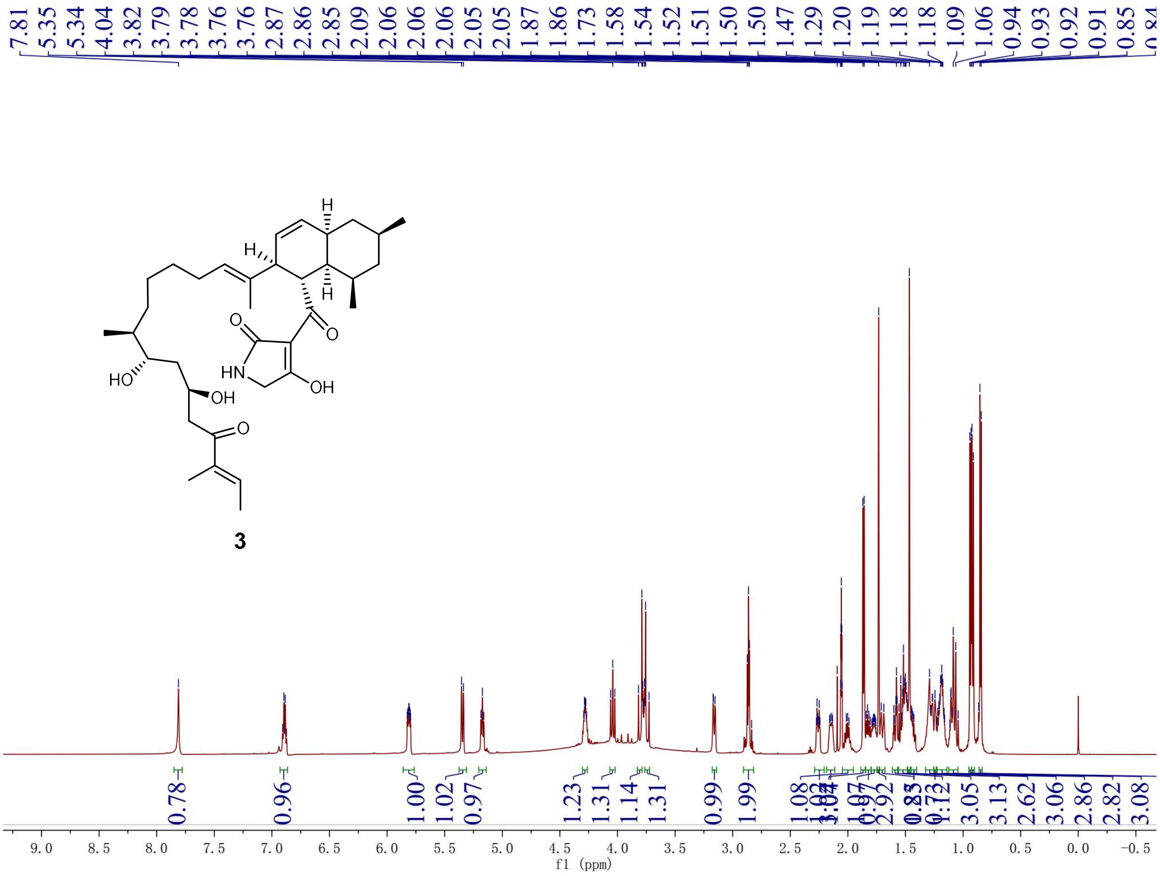

Supplementary Figure 33. ^1H NMR spectrum of **3** in acetone-d_6 (600 MHz).

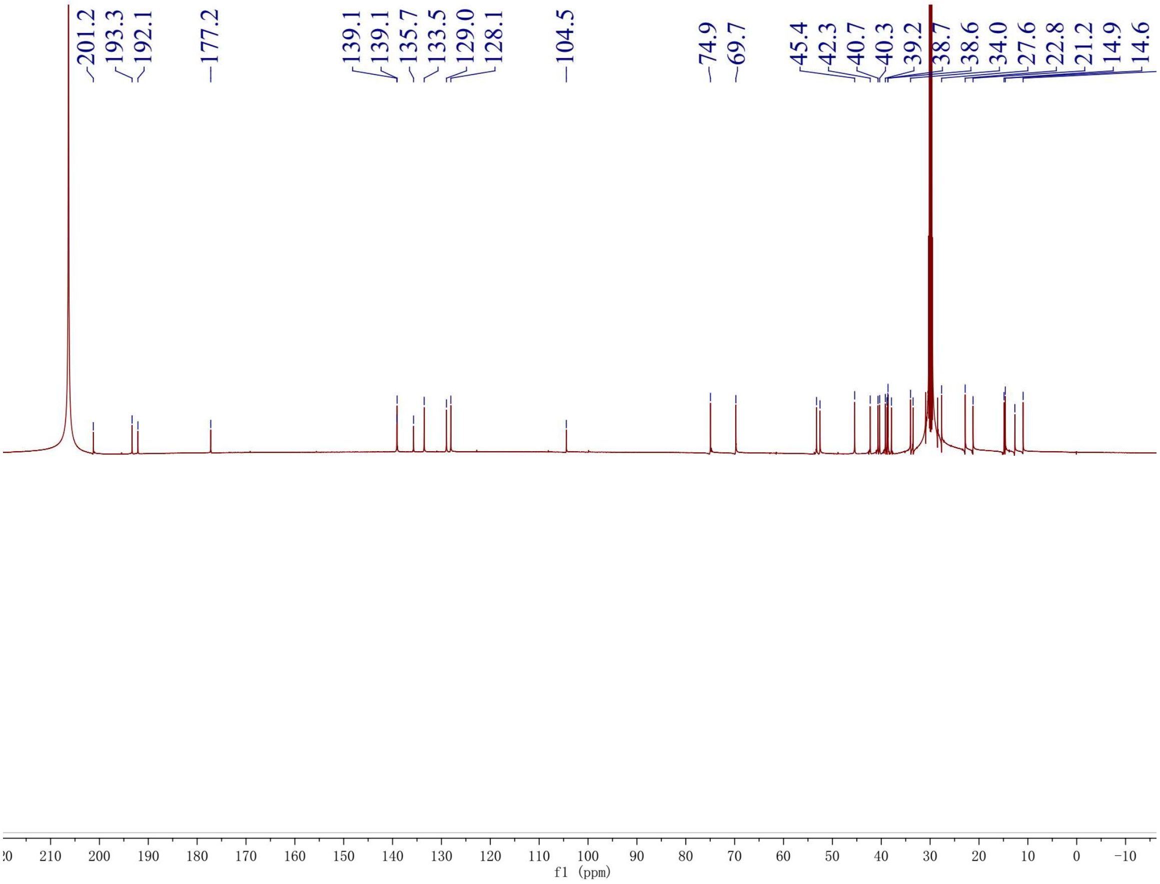

Supplementary Figure 34. ^{13}C NMR spectrum of **3** in acetone-d_6 (150 MHz).

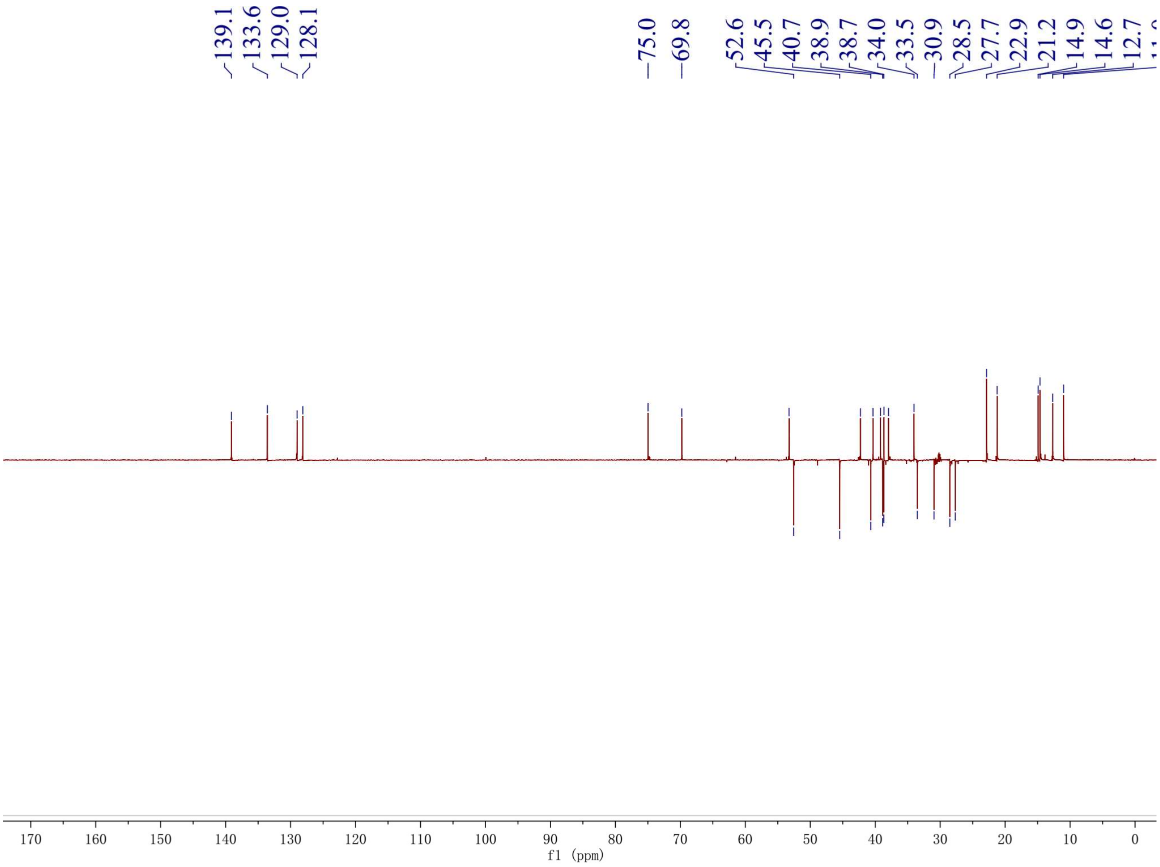

Supplementary Figure 35. DEPT-135 NMR spectrum of **3** in acetone-d_6 (150 MHz).

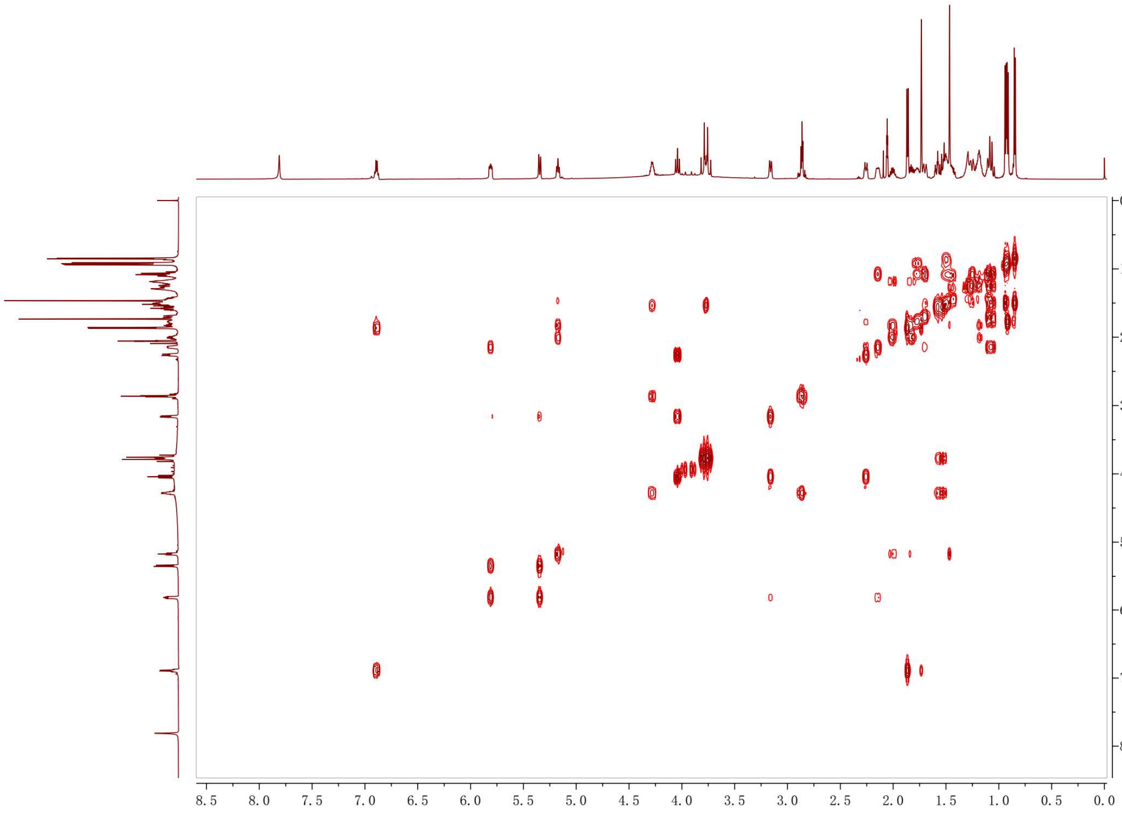

Supplementary Figure 36. ^{1}H-^{1}H COSY NMR spectrum of **3** in acetone-d_6.

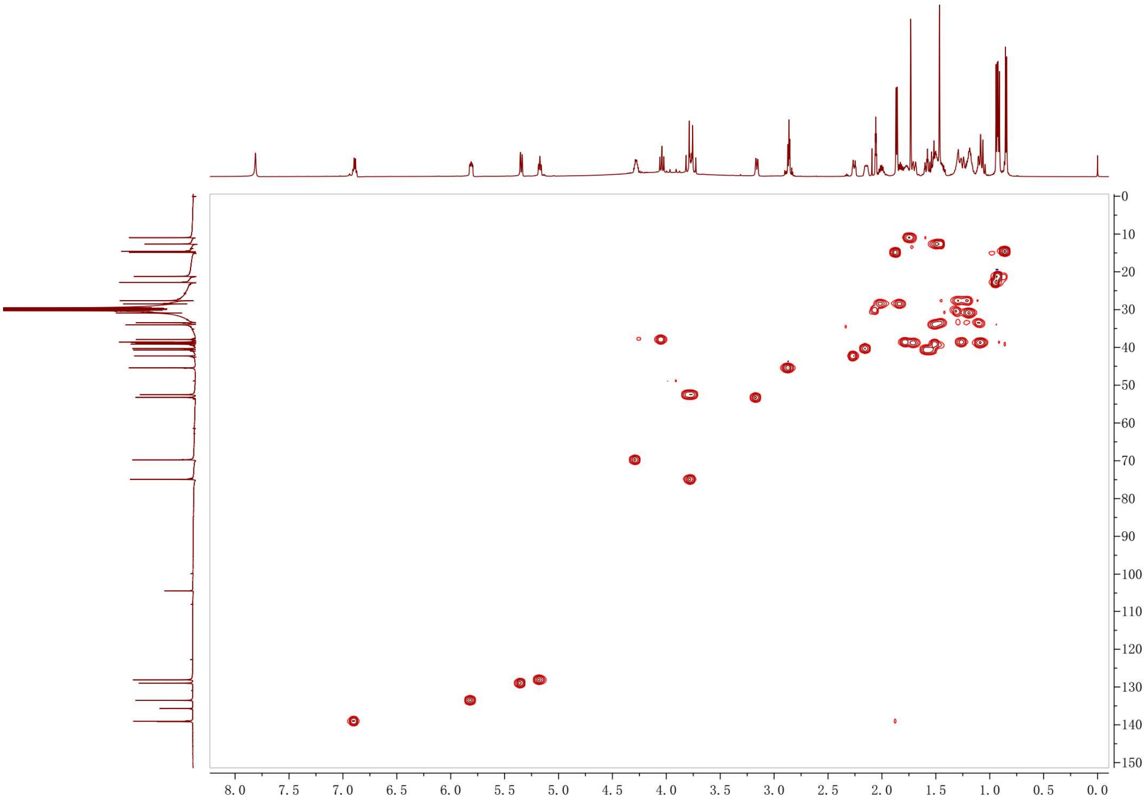

Supplementary Figure 37. HSQC NMR spectrum of **3** in acetone-d_6.

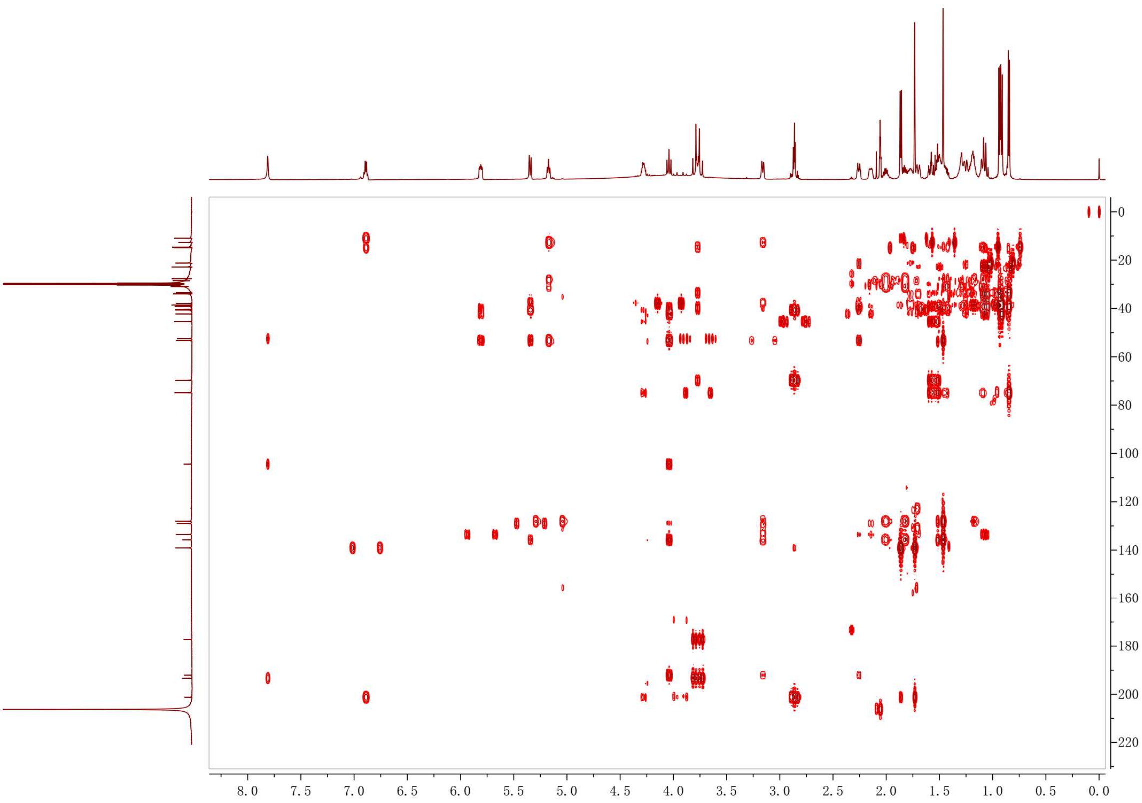

Supplementary Figure 38. HMBC NMR spectrum of **3** in acetone-d_6.

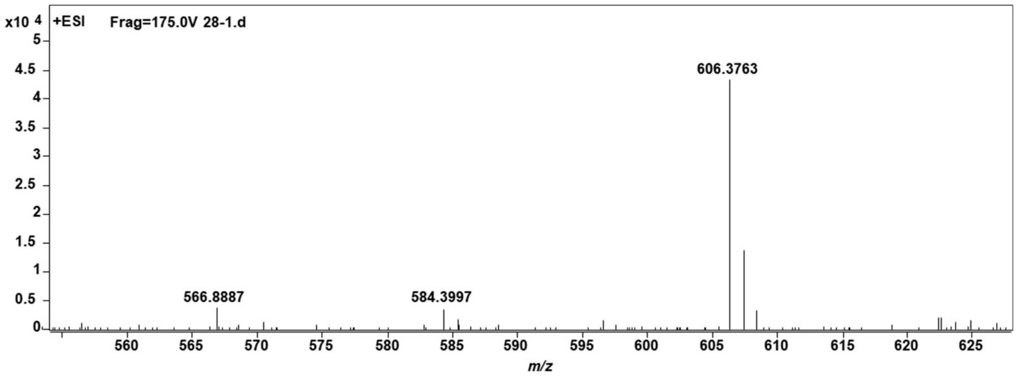

Supplementary Figure 39. HR-ESIMS spectrum of **3**.

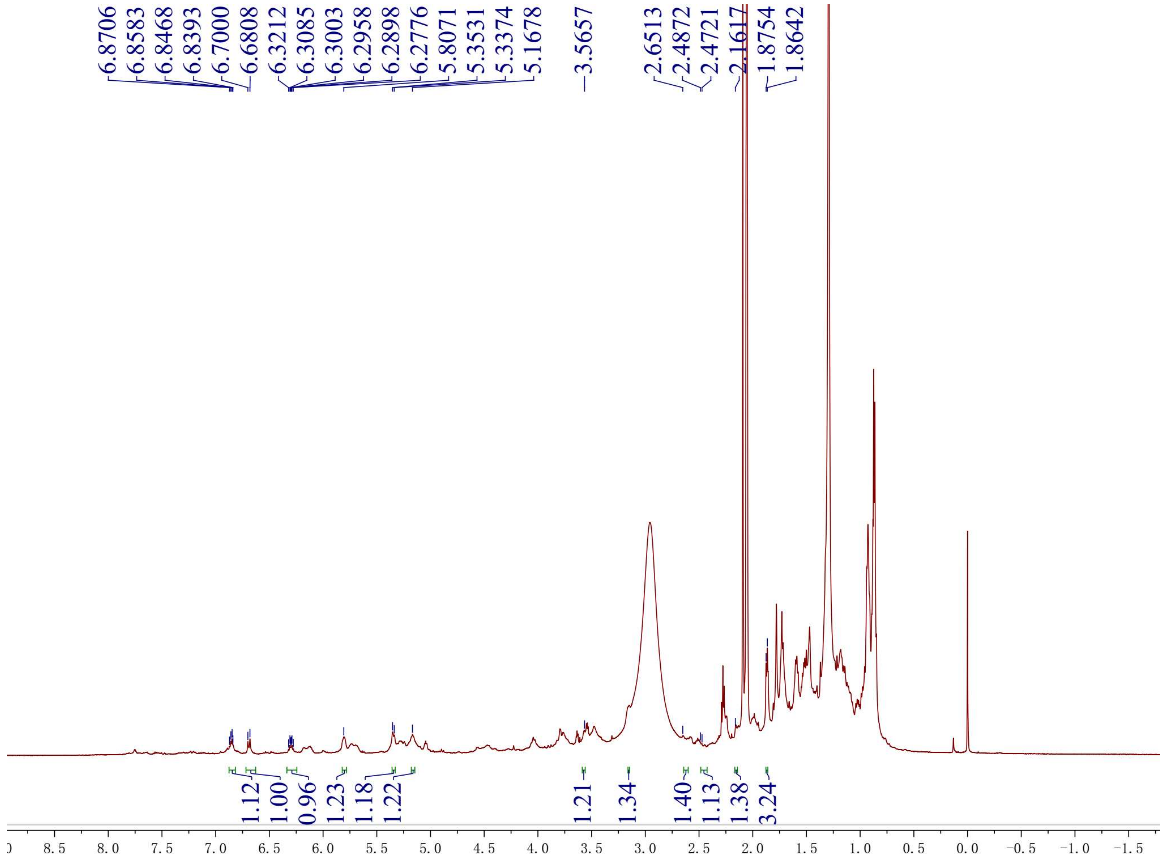

Supplementary Figure 40. ^{1}H NMR spectrum of **4** in acetone-d_6 (600 MHz).

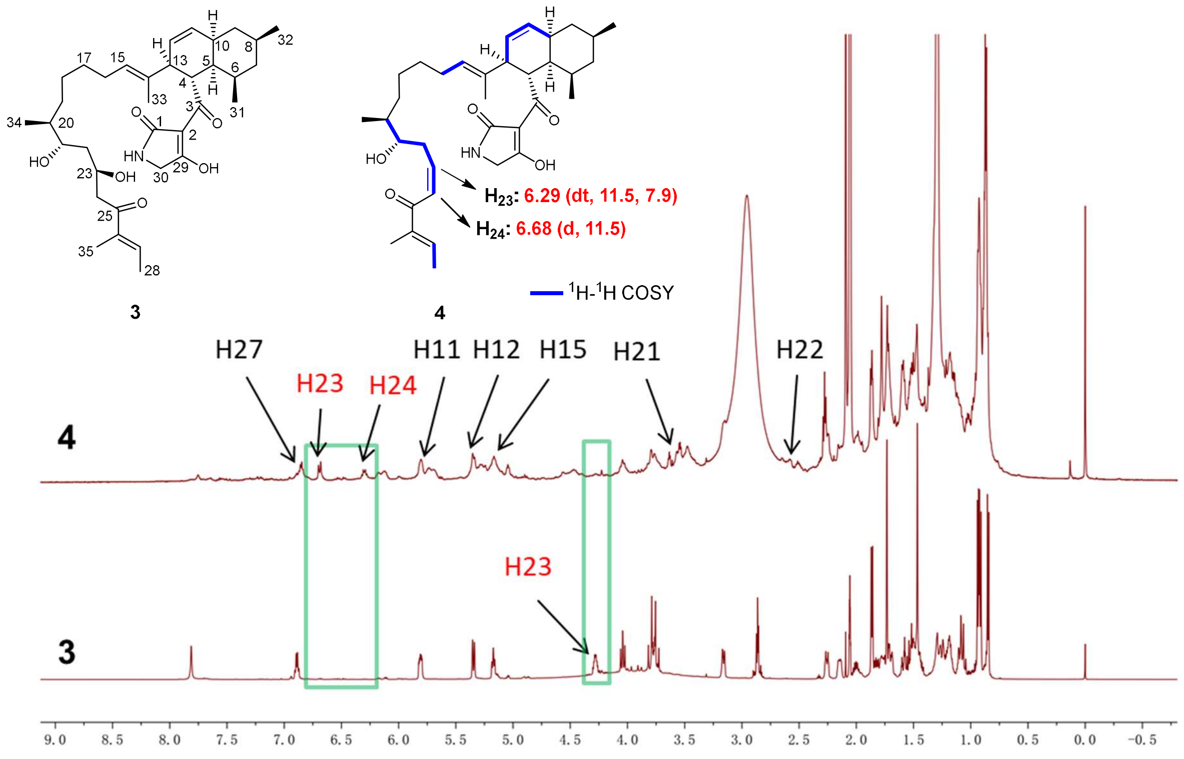

Supplementary Figure 41. Comparison of ^{1}H NMR spectra of **3** and **4** in acetone-d_6 (600 MHz).

a

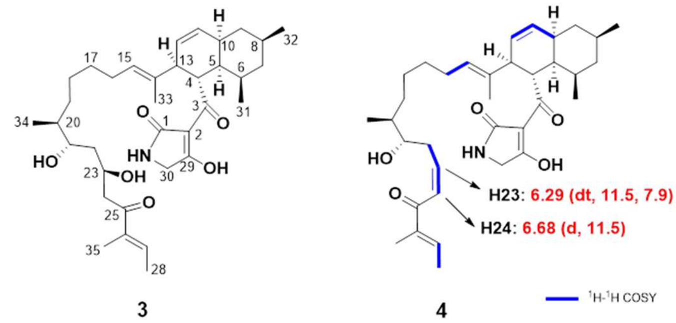

No.	3		4	
	δ_C	δ_H	δ_C	δ_H
10	40.3, CH	2.15	40.3	2.16
11	133.5, CH	5.81	133.4	5.81
12	129.0, CH	5.36	128.9	5.34
13	53.2, CH	3.16	53.4	3.18
15	128.1, CH	5.17	127.9	5.16
16	28.5, CH_2	2.0, 1.84	28.8	2.0
20	33.5, CH	1.50	34.0	1.47
21	74.9, CH	3.71	74.3	3.58
22	40.1, CH_2	1.63	35.3	2.64, 2.47
23	69.7, CH	4.28	144.0	6.29
24	45.4, CH_2	2.91, 2.86	126.6	6.68
27	139.1, CH	6.88	139.3	6.85
28	14.9, CH_3	1.86	14.9	1.89

b

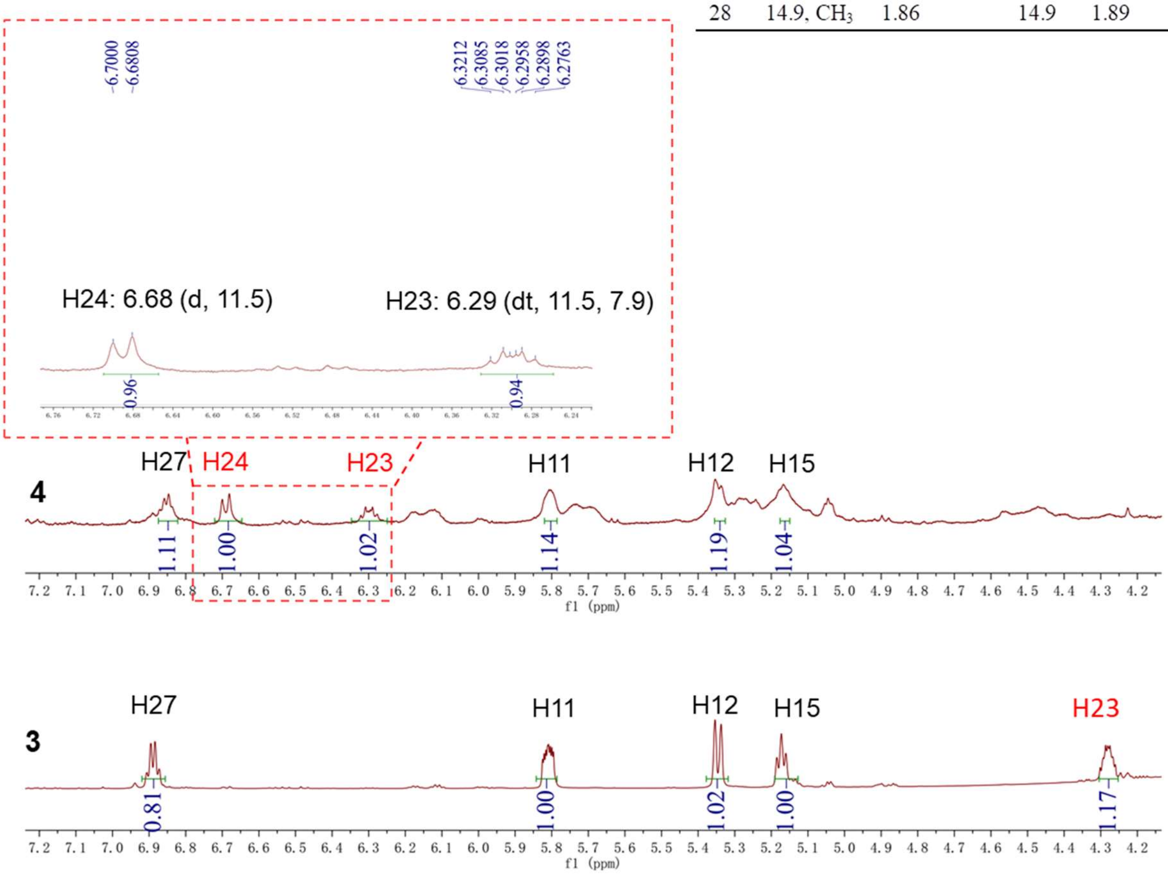

Supplementary Figure 42. Comparison of NMR data of compound **3** and **4**. a) Partial ^{1}H (600 MHz) and ^{13}C NMR (150 MHz) data of **3** and **4** (δ in ppm, acetone-d_6). b) Partially amplified ^{1}H NMR spectra of **3** and **4** in acetone-d_6 (600 MHz).

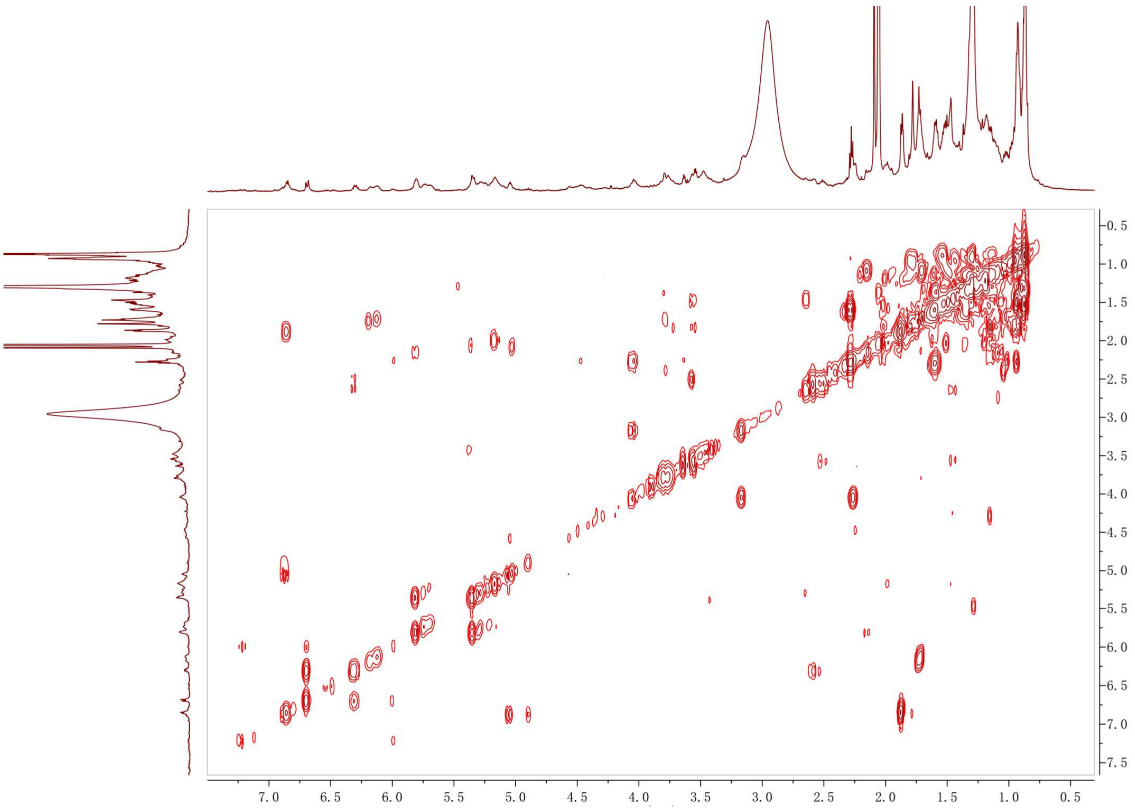

Supplementary Figure 43. ^{1}H-^{1}H COSY NMR spectrum of **4** in acetone-d_6.

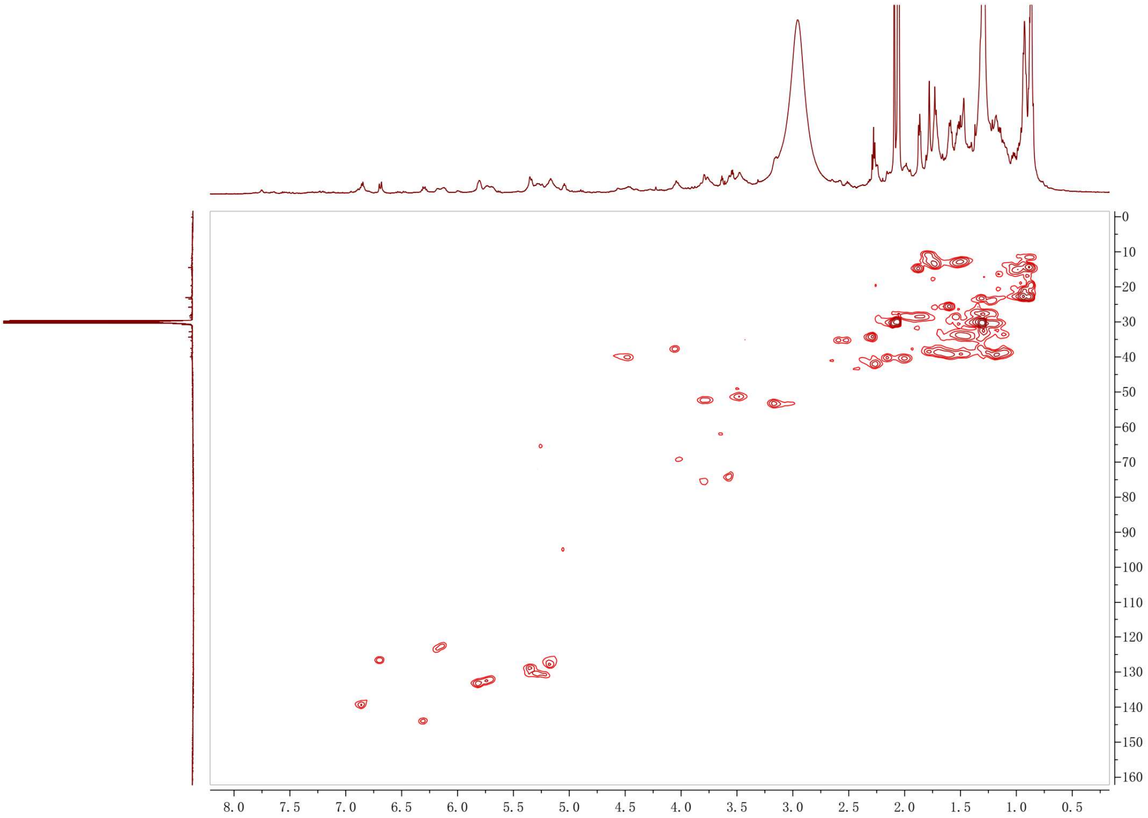

Supplementary Figure 44. HSQC NMR spectrum of **4** in acetone-d_6.

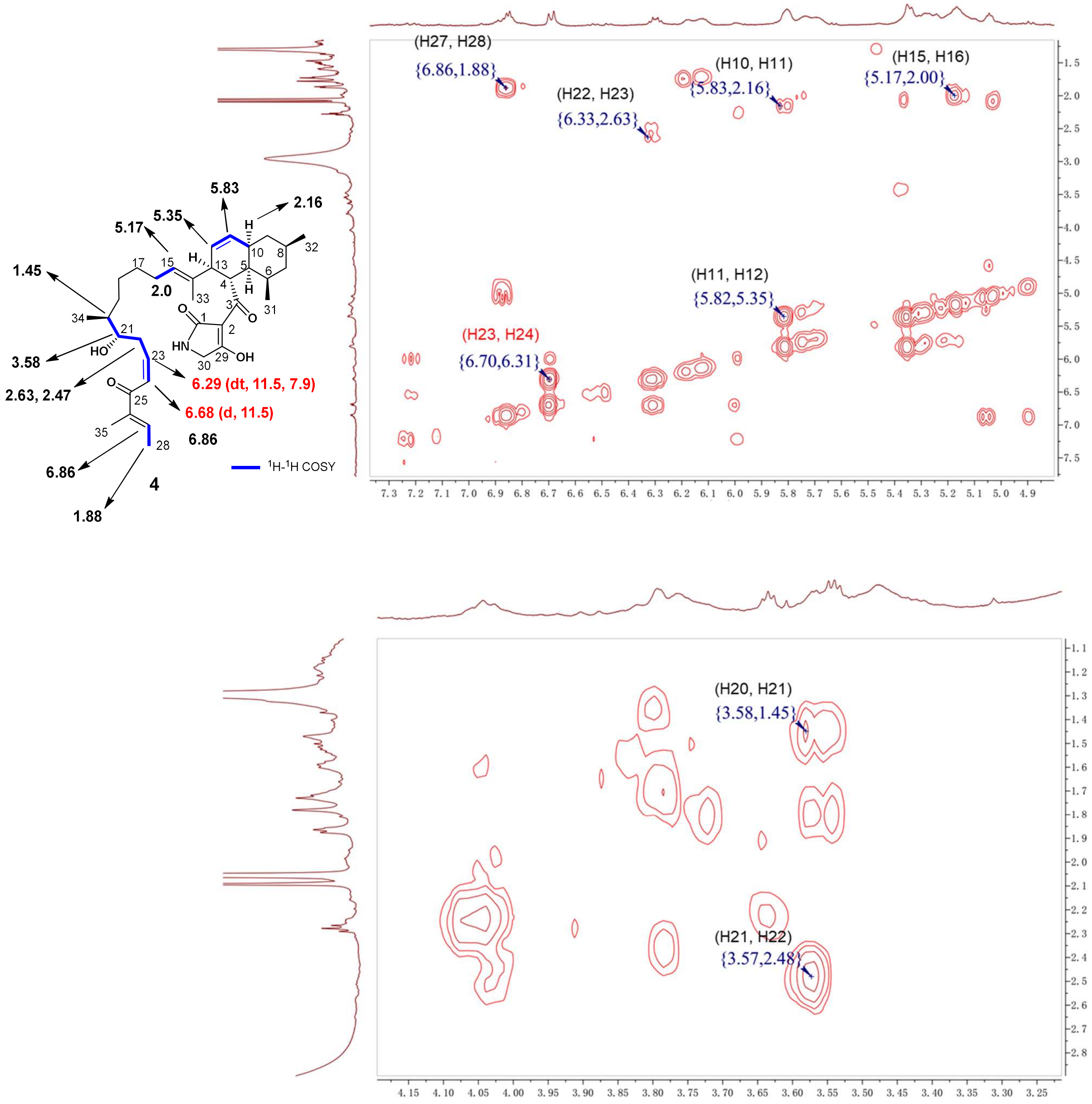

Supplementary Figure 45. Partially amplified ^{1}H-^{1}H COSY NMR spectra of **4** in acetone-d_6.

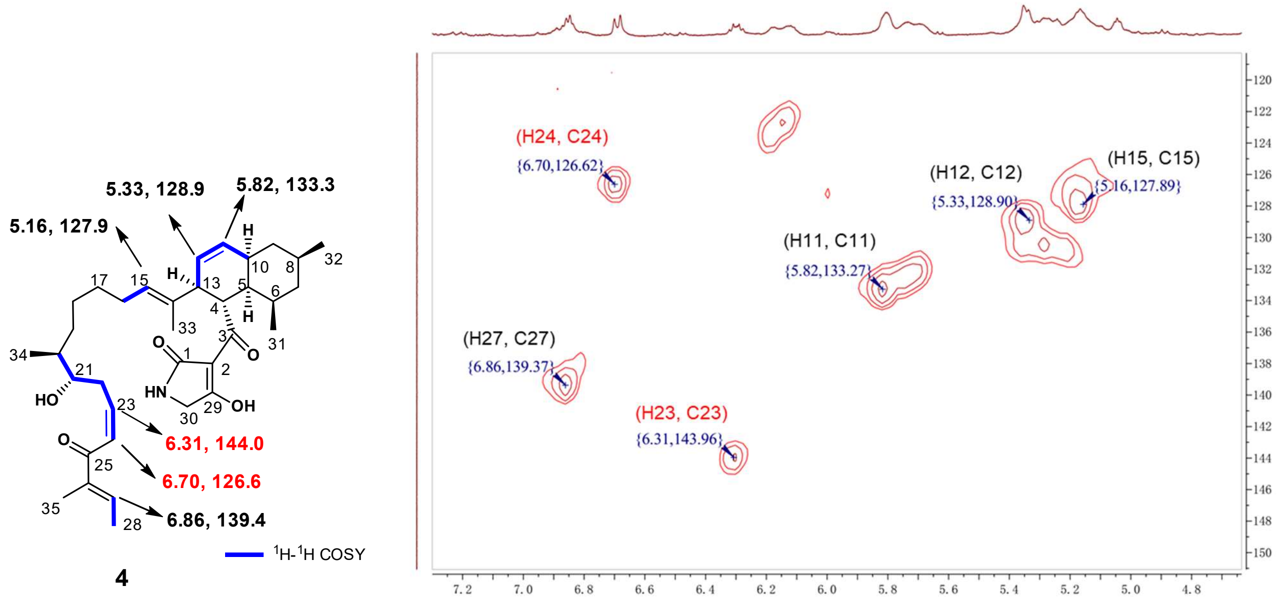

Supplementary Figure 46. Partially amplified HSQC NMR spectrum of **4** in acetone-d_6.

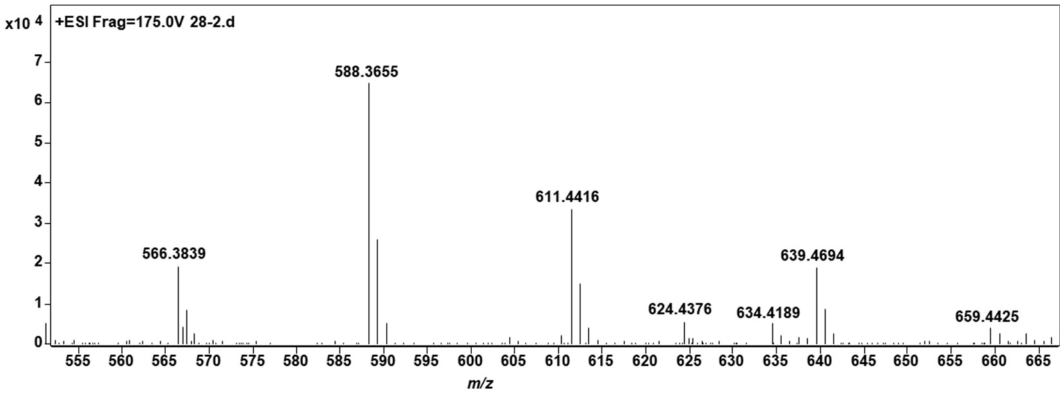

Supplementary Figure 47. HR-ESIMS spectrum of **4**.

Cartesian coordinates

3-conformer 1

G(Water) = -1871.690124 Hartree

C	7.034530	-4.050423	-1.080449
C	2.805025	2.775000	-1.833867
C	1.556315	1.873481	-1.975844
C	1.868401	0.485857	-2.583650
C	0.950536	-0.642028	-2.092513
C	-0.525256	-0.396586	-2.323240
C	-1.526009	-1.176689	-1.882828
C	-2.980629	-0.736000	-2.039252
C	-3.863836	-1.852631	-2.540009
C	-5.007843	-2.233514	-1.964426
C	-5.543487	-1.592968	-0.712196
C	6.592956	-3.037848	-0.070101
C	-5.227486	-2.481921	0.516105
C	-5.742878	-1.891877	1.836735
C	-5.245998	-0.446520	1.994372
C	-5.610858	0.431295	0.783897
C	-5.029038	-0.139402	-0.541645
C	-3.481647	-0.109321	-0.680467
C	-2.917382	1.296020	-0.647611
C	-1.813562	1.652407	0.221875
C	-5.313502	1.910953	1.067406
C	-5.342982	-2.764779	3.031935
C	5.362590	-2.514155	0.116430
C	-1.296859	-2.475915	-1.148938
C	4.140540	-2.871034	-0.697884
C	4.753654	3.609238	-0.445489
C	-0.996595	0.840511	1.161482
C	-0.171424	3.052011	1.207103
C	-1.311240	2.930395	0.243763
C	5.212055	-1.518261	1.230845
C	3.806361	-1.177154	1.698448
C	3.691417	0.156747	2.447633
C	4.110902	1.387592	1.634701
C	3.652236	1.377536	0.166275
C	3.457144	2.785344	-0.430791
N	-0.080048	1.695983	1.725099
O	6.183278	-1.019419	1.804225
O	4.531860	0.559798	-0.632841
O	-3.376285	2.174142	-1.415223
O	-1.732868	3.960900	-0.459311
O	-1.087922	-0.364496	1.418778
H	6.238733	-4.384809	-1.750184
H	7.848322	-3.637371	-1.692718
H	7.450330	-4.932230	-0.573471
H	7.376122	-2.685534	0.600370
H	3.359067	-3.326049	-0.076423
H	3.710582	-1.974996	-1.160241
H	4.375458	-3.578765	-1.495649
H	4.296764	0.121262	3.360340
H	3.708159	2.267565	2.150294
H	5.202465	1.471674	1.671943
H	2.692388	0.856726	0.109855
H	5.439524	0.891613	-0.540247
H	5.192337	3.704212	0.554274
H	5.504035	3.156889	-1.106922
H	4.559139	4.620432	-0.821543
H	2.754486	3.288170	0.249843
H	2.546864	3.814958	-2.073148
H	3.556471	2.471141	-2.575737
H	1.080877	1.738274	-0.994921
H	0.803044	2.381494	-2.589797
H	2.898635	0.209572	-2.337176
H	1.819883	0.556285	-3.679894
H	1.125829	-0.780909	-1.014182
H	1.262830	-1.585200	-2.564594
H	-0.786384	0.521810	-2.851573
H	-1.846315	-2.494676	-0.201179
H	-1.657446	-3.325639	-1.744006
H	-0.241498	-2.645997	-0.922880
H	-3.010405	0.060778	-2.795643
H	-3.508178	-2.358104	-3.438441
H	-5.576442	-3.063871	-2.383255
H	-6.639577	-1.536290	-0.796377
H	-4.139987	-2.624144	0.586067
H	-5.657501	-3.480580	0.356892
H	-6.843413	-1.860863	1.785278
H	-5.735751	-2.354967	3.971276
H	-5.724431	-3.788130	2.923017
H	-4.249532	-2.821372	3.121379
H	-5.678186	-0.006226	2.904046
H	-4.154722	-0.446280	2.139012
H	-6.702976	0.356104	0.656782
H	-5.954621	2.264701	1.884312
H	-4.276109	2.069896	1.383086
H	-5.497445	2.538353	0.188765
H	-5.437908	0.457263	-1.368085
H	-3.032016	-0.668982	0.140064
H	-0.395284	3.786483	1.991405
H	0.742223	3.373292	0.694902
H	3.104007	-1.181473	0.863614
H	0.763679	1.331561	2.169624
H	3.489452	-1.996806	2.362784
O	2.307519	0.366327	2.824127
H	2.002849	-0.389254	3.353537
H	-2.479668	3.596948	-1.025027

3-conformer 2

G(Water) = -1871.669129 Hartree

C	5.720828	-2.227858	0.512055
C	5.303896	-0.716032	0.442296
C	6.476763	0.205235	-0.025904
C	7.788364	-0.319524	0.590125
C	8.193071	-1.743807	0.110582
C	6.959327	-2.553506	-0.344320
C	4.597465	-3.196813	0.246282
C	3.313785	-2.855849	0.087081
C	2.842569	-1.422038	0.143193
C	3.998535	-0.501313	-0.363520
C	9.278789	-1.726957	-0.975437
C	6.604433	0.446039	-1.545387
C	1.553958	-1.162622	-0.626811
C	0.515660	-0.595623	0.012628
C	1.577715	-1.477521	-2.104671
C	-0.778275	-0.097832	-0.571603
C	-0.761397	1.442242	-0.704533
C	-2.046180	2.044475	-1.294924
C	-3.280794	1.881023	-0.395925
C	-4.575637	2.515974	-0.954504
C	-4.519669	4.045230	-0.796382
C	-5.831311	1.882366	-0.273102
O	-6.921929	2.819268	-0.124657
H	-6.644769	3.529458	0.475002
C	-6.480859	0.733497	-1.064699
C	-5.773063	-0.621074	-1.228959
C	-5.341566	-1.294168	0.099239
C	-6.516126	-1.491520	1.046560
O	-6.647177	-0.736447	2.011914
C	-7.508847	-2.570978	0.736599

C	-8.759992	-2.400676	1.216660
C	-9.954852	-3.284193	1.040219
C	-7.054128	-3.743888	-0.103707
O	-4.679777	-0.597911	-2.159460
H	-3.948012	-0.083485	-1.786394
C	3.518988	0.937706	-0.377836
O	3.425561	1.526787	-1.482526
C	3.100890	1.677534	0.808453
C	3.030579	1.331875	2.256573
N	2.451488	2.405777	2.882567
O	3.393767	0.303930	2.838247
C	2.171334	3.521414	1.998885
H	2.404524	2.468078	3.890478
C	2.596844	2.954246	0.679674
O	2.449677	3.647446	-0.426847
H	-9.756299	-4.187350	0.458631
H	-10.34387	-3.585292	2.022829
H	-10.76400	-2.728033	0.547247
H	-8.936531	-1.498454	1.801328
H	-6.169964	-4.222404	0.335201
H	-7.831374	-4.505813	-0.188968
H	-6.782164	-3.440230	-1.121811
H	-4.875511	-2.251240	-0.154000
H	-4.600188	-0.682331	0.618206
H	-6.500208	-1.276663	-1.718225
H	-7.439654	0.526179	-0.573151
H	-6.720393	1.104261	-2.069539
H	-5.551933	1.529721	0.729967
H	-4.635916	2.301627	-2.031690
H	-3.622027	4.447900	-1.276656
H	-5.385207	4.531213	-1.256279
H	-4.478331	4.335841	0.262783
H	-3.436946	0.814796	-0.193901
H	-3.074048	2.318271	0.592276
H	-1.873570	3.110556	-1.487184
H	-2.242383	1.588314	-2.278030
H	-0.570080	1.887826	0.282620
H	0.089757	1.721560	-1.338987
H	-0.974948	-0.537279	-1.556990
H	-1.605755	-0.402184	0.082932
H	0.637872	-0.381724	1.076492
H	2.071476	-2.438997	-2.292756
H	0.577590	-1.519404	-2.543665
H	2.148427	-0.715140	-2.652036
H	2.662062	-1.165974	1.193071
H	2.557150	-3.624867	-0.066520
H	4.878835	-4.250522	0.220764
H	6.032497	-2.408502	1.554661
H	5.078881	-0.418763	1.469426
H	4.184979	-0.751529	-1.412542
H	6.285740	1.193333	0.419414
H	7.182786	-3.626422	-0.272394
H	6.674157	-0.479111	-2.122883
H	5.755625	1.012216	-1.936695
H	7.512589	1.027911	-1.746238
H	8.601753	0.388955	0.383224
H	7.663581	-0.331514	1.681859
H	8.614935	-2.267450	0.980923
H	9.543775	-2.748351	-1.279145
H	8.941430	-1.189088	-1.869090
H	10.190762	-1.235575	-0.613317
H	2.754035	4.416798	2.250211
H	1.108352	3.792028	1.984124
H	6.734898	-2.363647	-1.400997
H	2.787642	3.035092	-1.154212

IM1

G(Water) = -1871.237952 Hartree

C	7.242001	-3.848062	-0.923157
C	2.689446	2.737652	-1.904560
C	1.486725	1.779220	-2.066983
C	1.883700	0.386574	-2.615693
C	1.027541	-0.775147	-2.091362
C	-0.456682	-0.618197	-2.344505
C	-1.430784	-1.377697	-1.816794
C	-2.899132	-1.004019	-2.010990
C	-3.740853	-2.207382	-2.362470
C	-4.876187	-2.544166	-1.742027
C	-5.440787	-1.746398	-0.596914
C	6.713556	-2.847923	0.057629
C	-5.111780	-2.434897	0.751478
C	-5.651083	-1.669712	1.970055
C	-5.212393	-0.197349	1.909110
C	-5.601815	0.466075	0.576441
C	-4.962592	-0.270020	-0.635660
C	-3.413247	-0.224973	-0.747401
C	-2.870787	1.208395	-0.864452
C	-1.897137	1.698395	0.079323
C	-5.399032	1.987324	0.628143
C	-5.222178	-2.337475	3.281950
C	5.451465	-2.388928	0.196083
C	-1.155885	-2.578457	-0.943144
C	4.278722	-2.821145	-0.653443
C	4.552646	3.681942	-0.467664
C	-1.206060	0.930691	1.120253
C	-0.344704	3.111627	1.233681
C	-1.397029	3.039839	0.119036
C	5.209357	-1.389544	1.291590
C	3.772212	-1.118938	1.704966
C	3.555507	0.214061	2.433815
C	3.942844	1.457734	1.624126
C	3.537340	1.409629	0.141260
C	3.296438	2.797926	-0.482786
N	-0.371769	1.787506	1.828840
O	6.132514	-0.834307	1.891971
O	4.485408	0.625741	-0.614394
O	-3.292011	1.906166	-1.811942
O	-1.685207	4.053842	-0.550271
O	-1.278656	-0.280906	1.407016
H	6.487711	-4.233902	-1.613016
H	8.051991	-3.399600	-1.515244
H	7.687773	-4.699389	-0.390198
H	7.454108	-2.445820	0.748248
H	3.501680	-3.309475	-0.051990
H	3.817432	-1.955488	-1.142520
H	4.578065	-3.525234	-1.432667
H	4.124563	0.218866	3.370815
H	3.476379	2.320172	2.115146
H	5.026670	1.598145	1.699233
H	2.605931	0.843213	0.059470
H	5.370310	1.007576	-0.497456
H	4.952596	3.811652	0.544507
H	5.345838	3.256887	-1.096724
H	4.323835	4.677540	-0.865674
H	2.546630	3.274201	0.164912
H	2.391351	3.761644	-2.166436
H	3.475687	2.458980	-2.620303
H	0.968617	1.665332	-1.104596
H	0.743594	2.233845	-2.733155
H	2.925760	0.179591	-2.351888
H	1.842105	0.410660	-3.714478
H	1.202625	-0.866876	-1.008336
H	1.400035	-1.714410	-2.528121

H	-0.750693	0.228157	-2.967154
H	-1.696819	-2.498318	0.005898
H	-1.495916	-3.501240	-1.432638
H	-0.093328	-2.693431	-0.712957
H	-2.957848	-0.308542	-2.860153
H	-3.365591	-2.822372	-3.182059
H	-5.416687	-3.442460	-2.042784
H	-6.538008	-1.736065	-0.694104
H	-4.021497	-2.534534	0.844229
H	-5.515068	-3.457647	0.743291
H	-6.752219	-1.688988	1.918385
H	-5.633257	-1.806769	4.150500
H	-5.563039	-3.379894	3.329720
H	-4.127589	-2.337329	3.375987
H	-5.672046	0.355583	2.741268
H	-4.123547	-0.129989	2.051213
H	-6.686480	0.308236	0.452478
H	-6.082844	2.425187	1.367100
H	-4.379279	2.255195	0.921670
H	-5.598585	2.451590	-0.343937
H	-5.368370	0.187124	-1.549122
H	-2.981099	-0.688590	0.136656
H	-0.605319	3.899812	1.951405
H	0.632596	3.357655	0.802157
H	3.102052	-1.169187	0.845561
H	0.476626	1.380670	2.218917
H	3.475411	-1.948192	2.366870
O	2.148785	0.353813	2.745492
H	1.856723	-0.421026	3.253607

IM2

G(Water) = -1871.223896 Hartree

C	1.023749	5.164743	-0.724352
C	-3.702319	-2.813371	-0.466560
C	-2.194760	-2.853346	-0.753807
C	-1.694407	-4.302101	-0.880382
C	-0.348186	-4.456623	-1.618305
C	0.846126	-3.886966	-0.896600
C	1.521975	-2.755675	-1.167577
C	2.791781	-2.422146	-0.381263
C	3.991901	-2.557467	-1.292288
C	4.944276	-1.632755	-1.438304
C	4.899950	-0.312526	-0.721382
C	-0.075630	4.144835	-0.792098
C	4.368864	0.783280	-1.675170
C	4.337559	2.170475	-1.022716
C	3.542597	2.100582	0.290655
C	4.091152	1.023897	1.247275
C	4.092610	-0.394080	0.601889
C	2.699338	-1.042391	0.368370
C	1.969869	-1.363712	1.686849
C	0.603542	-0.950507	1.893875
C	3.422080	1.124907	2.625109
C	3.782674	3.226082	-1.985028
C	-1.299277	4.197078	-0.220522
C	1.136560	-1.777526	-2.254354
C	-1.778584	5.369905	0.608437
C	-5.843487	-1.572165	-0.004103
C	-0.171635	-0.003357	1.095929
C	-1.623805	-0.775171	2.759554
C	-0.247997	-1.430191	2.945471
C	-2.237175	3.053219	-0.375722
C	-3.508317	3.075326	0.089141
C	-4.488321	1.925717	0.152530
C	-4.628549	1.002087	-1.084057
C	-3.891131	-0.352943	-1.159268
C	-4.319297	-1.435501	-0.147408
N	-1.417071	0.119157	1.642460
O	-1.797460	1.945697	-1.051773
O	-4.134727	-0.893372	-2.484305
O	2.579991	-2.061217	2.524239
O	-0.045308	-2.236707	3.873908
O	0.185199	0.656581	0.080667
H	1.879038	4.762538	-0.164884
H	1.398287	5.390164	-1.731303
H	0.726740	6.105704	-0.255198
H	0.175351	3.257075	-1.368957
H	-2.657302	5.846596	0.155460
H	-2.071173	5.047504	1.615385
H	-1.010785	6.137233	0.718488
H	-3.861241	3.984744	0.563933
H	-5.477161	2.374662	0.312412
H	-5.692470	0.792823	-1.237531
H	-4.302980	1.587349	-1.951536
H	-2.817373	-0.183185	-1.046171
H	-3.731300	-0.293646	-3.131937
H	-6.293858	-0.667571	0.417739
H	-6.315970	-1.771972	-0.974015
H	-6.088829	-2.406709	0.664840
H	-3.924036	-1.107777	0.817393
H	-3.916732	-3.474742	0.386955
H	-4.227503	-3.246935	-1.328638
H	-1.991327	-2.317294	-1.689532
H	-1.634391	-2.328268	0.031426
H	-2.444415	-4.887148	-1.433362
H	-1.626407	-4.757514	0.118773
H	-0.451368	-4.013883	-2.616574
H	-0.168892	-5.530759	-1.775381
H	1.198688	-4.492557	-0.057868
H	0.425744	-2.199494	-2.969488
H	0.674269	-0.885095	-1.814529
H	2.021938	-1.441697	-2.808454
H	2.909933	-3.199162	0.385892
H	4.048152	-3.495885	-1.845811
H	5.772414	-1.804855	-2.126682
H	5.930486	-0.030888	-0.452919
H	3.353977	0.514401	-2.000635
H	4.990435	0.806037	-2.581807
H	5.375339	2.447371	-0.773054
H	3.824783	4.229204	-1.544879
H	4.352934	3.244435	-2.922975
H	2.733879	3.014717	-2.232534
H	3.575085	3.078341	0.793625
H	2.485266	1.894475	0.072569
H	5.158008	1.256590	1.403162
H	3.659606	2.094024	3.082883
H	2.331786	1.052791	2.554458
H	3.765661	0.332518	3.299193
H	4.644600	-1.059918	1.279329
H	2.092543	-0.358625	-0.220128
H	-1.926586	-0.243684	3.669875
H	-2.377426	-1.543721	2.542160
H	-2.187413	0.620075	1.208865
H	-0.972768	1.543436	-0.634623
O	-4.201580	1.109043	1.329620
H	-4.186993	1.704199	2.097614

TS1

G(Water) = -1871.193843 Hartree

C	-1.636885	5.754764	0.585728
C	-1.982479	-3.912289	-0.638899
C	-0.973766	-3.445694	0.424646

C	0.085354	-4.503312	0.781831
C	1.213365	-4.651033	-0.270859
C	2.220477	-3.528074	-0.204546
C	2.302647	-2.422675	-0.966193
C	3.378988	-1.372210	-0.678311
C	4.155728	-1.028199	-1.928306
C	4.448296	0.216777	-2.317238
C	4.013111	1.434501	-1.547000
C	-1.936928	4.332221	0.225865
C	2.799048	2.101379	-2.240945
C	2.304872	3.360549	-1.512277
C	2.056755	3.046552	-0.028012
C	3.289787	2.420230	0.647341
C	3.732962	1.104615	-0.056138
C	2.768379	-0.112875	0.029189
C	2.464305	-0.540482	1.472619
C	1.104790	-0.591705	1.967286
C	3.111082	2.343760	2.170626
C	1.056431	3.941833	-2.186485
C	-3.131551	3.701717	0.193759
C	1.352797	-2.106700	-2.097705
C	-4.449661	4.357211	0.542519
C	-4.172159	-3.607609	-1.861485
C	-0.109869	-0.180968	1.324332
C	-0.816066	-0.940732	3.342642
C	0.708014	-1.082763	3.266541
C	-3.124577	2.249153	-0.189861
C	-4.275699	1.448712	0.088164
C	-4.438935	0.128784	-0.304003
C	-3.788370	-0.486306	-1.523503
C	-2.704559	-1.562970	-1.352753
C	-3.175120	-2.956678	-0.890550
N	-1.197075	-0.321420	2.079386
O	-2.080170	1.766756	-0.732832
O	-2.068427	-1.620918	-2.655657
O	3.424853	-0.882940	2.186661
O	1.374928	-1.542486	4.207371
O	-0.176124	0.290960	0.095140
H	-2.522392	6.352480	0.816734
H	-0.966054	5.789729	1.455809
H	-1.095088	6.244858	-0.235425
H	-1.068101	3.733390	-0.040065
H	-5.216433	4.126622	-0.206285
H	-4.831960	4.007706	1.510817
H	-4.360342	5.444509	0.602401
H	-1.046777	0.762310	-0.137035
H	-5.032781	1.865741	0.745380
H	-5.365624	-0.344705	0.003422
H	-4.595283	-0.897281	-2.143677
H	-3.311628	0.312587	-2.094764
H	-1.980224	-1.187164	-0.630100
H	-1.322842	-2.238210	-2.604933
H	-5.083994	-3.010833	-1.978303
H	-3.727843	-3.740838	-2.855447
H	-4.473664	-4.595543	-1.492243
H	-3.668263	-2.785527	0.072208
H	-2.395590	-4.879743	-0.318812
H	-1.465478	-4.124491	-1.588453
H	-0.457642	-2.536623	0.097655
H	-1.532152	-3.166953	1.326853
H	-0.406562	-5.476713	0.922435
H	0.544421	-4.245068	1.746337
H	0.772618	-4.740369	-1.270557
H	1.739821	-5.597114	-0.084206
H	2.940055	-3.622059	0.611657
H	1.895996	-1.833402	-3.010223
H	0.705990	-2.955595	-2.336290
H	0.716708	-1.250897	-1.839864
H	4.089624	-1.817696	0.030954
H	4.487275	-1.878351	-2.526237
H	5.001753	0.382043	-3.242217
H	4.836873	2.165317	-1.568220
H	1.976399	1.374929	-2.301719
H	3.068041	2.349157	-3.277589
H	3.107764	4.114377	-1.563624
H	0.727560	4.861473	-1.685746
H	1.247865	4.180732	-3.240732
H	0.225406	3.225923	-2.145905
H	1.782604	3.971204	0.500549
H	1.198904	2.367154	0.064771
H	4.129324	3.114243	0.474404
H	3.050321	3.358321	2.585103
H	2.191842	1.819949	2.451347
H	3.950078	1.826400	2.648996
H	4.682527	0.793479	0.401768
H	1.844560	0.149988	-0.476094
H	-1.105783	-0.324946	4.202103
H	-1.289463	-1.924111	3.449910
O	-3.595041	-0.983439	1.433111
H	-2.232452	-0.517259	1.704337
H	-4.123925	-0.433542	2.034651

TS1a

G(Water) = -1871.182271 Hartree

C	-1.828778	3.849573	2.247208
C	3.853121	-1.660589	0.630350
C	2.687396	-1.423575	1.598223
C	2.412900	-2.621290	2.515009
C	1.328420	-2.340162	3.581716
C	-0.069344	-2.236979	3.020020
C	-0.728901	-1.132745	2.624975
C	-2.145744	-1.257532	2.064900
C	-3.097500	-0.307568	2.758370
C	-4.080607	0.352912	2.137787
C	-4.369528	0.210902	0.667417
C	-0.454097	3.515636	1.750070
C	-4.003283	1.507317	-0.098324
C	-4.337729	1.407987	-1.594659
C	-3.637213	0.175751	-2.188220
C	-4.044007	-1.110786	-1.450690
C	-3.696330	-1.053437	0.064515
C	-2.200395	-1.089807	0.510330
C	-1.426799	-2.261565	-0.103420
C	-0.326928	-2.033268	-1.021358
C	-3.595725	-2.366635	-2.212611
C	-3.999823	2.689651	-2.363357
C	0.003868	3.485014	0.482460
C	-0.154624	0.260582	2.675020
C	-0.849740	3.828591	-0.716202
C	5.230136	-0.920431	-1.356112
C	0.306190	-0.812290	-1.410961
C	1.368180	-2.363945	-2.688427
C	0.327143	-3.064257	-1.802757
C	1.435974	3.019877	0.270651
C	1.884723	2.889392	-1.093350
C	3.149274	2.504250	-1.561414
C	4.326101	2.038201	-0.739699
C	4.197953	0.837811	0.220091
C	4.064558	-0.553905	-0.425196
N	1.213077	-0.956117	-2.366258
O	2.164541	2.795710	1.269587
O	5.309360	0.880442	1.150013
O	-1.784670	-3.413701	0.195409

O	0.150271	-4.289422	-1.841900
O	0.070267	0.382366	-0.875429
H	-1.773953	4.585524	3.060794
H	-2.497500	4.241872	1.476654
H	-2.301572	2.950898	2.669401
H	0.255948	3.222330	2.520851
H	-0.427572	4.661941	-1.292062
H	-0.935427	2.976214	-1.399556
H	-1.864309	4.109523	-0.428410
H	3.461871	2.993103	-2.478016
H	5.176854	1.883761	-1.411773
H	4.577256	2.895588	-0.096692
H	3.328496	1.022361	0.845828
H	6.132544	0.734605	0.655546
H	5.319767	-0.210349	-2.182625
H	6.187992	-0.962532	-0.818450
H	5.066702	-1.914530	-1.790834
H	3.162272	-0.506206	-1.035194
H	3.681716	-2.606278	0.094025
H	4.779230	-1.796145	1.206796
H	2.904204	-0.550690	2.227166
H	1.778247	-1.178356	1.033873
H	3.344946	-2.900207	3.028693
H	2.116560	-3.491408	1.910265
H	1.604916	-1.428140	4.126883
H	1.344522	-3.155701	4.317733
H	-0.579762	-3.193167	2.891586
H	-0.018462	0.662995	1.665723
H	-0.832660	0.948969	3.193165
H	0.815959	0.301380	3.173042
H	-2.484209	-2.282505	2.269210
H	-2.948113	-0.175017	3.830433
H	-4.714435	1.038832	2.700965
H	-5.457736	0.077397	0.554286
H	-2.931874	1.716578	0.024256
H	-4.535927	2.355176	0.354749
H	-5.424500	1.242893	-1.680373
H	-4.343734	2.622008	-3.403535
H	-4.475400	3.566820	-1.905764
H	-2.919085	2.865955	-2.381226
H	-3.889851	0.081056	-3.254092
H	-2.546052	0.311376	-2.133746
H	-5.146448	-1.134541	-1.462833
H	-4.130682	-2.421619	-3.169415
H	-2.525169	-2.355844	-2.437324
H	-3.810003	-3.279210	-1.646095
H	-4.169447	-1.932521	0.525120
H	-1.728321	-0.146849	0.240608
H	1.174496	-2.562965	-3.748115
H	2.379653	-2.712728	-2.446993
H	0.734127	1.060597	-1.162279
H	1.927897	-0.169962	-2.637756
H	1.245265	3.322773	-1.855940
O	2.929552	0.908027	-2.909903
H	2.531610	1.424318	-3.631095

IM3

G(Water) = -1871.227303 Hartree

C	-1.068486	5.245092	0.807650
C	-2.616449	-3.396662	0.006844
C	-1.502778	-2.779444	0.870529
C	-0.604463	-3.833754	1.544162
C	0.430302	-4.493656	0.595012
C	1.646456	-3.628643	0.360567
C	1.891209	-2.774233	-0.648528
C	3.156389	-1.909511	-0.634631
C	3.826329	-1.891323	-1.989235
C	4.222075	-0.781379	-2.621582
C	4.049636	0.593803	-2.032444
C	-1.579928	3.975448	0.209140
C	2.833991	1.315614	-2.668420
C	2.594313	2.724192	-2.097935
C	2.565843	2.689924	-0.560315
C	3.806863	1.998767	0.029728
C	3.947590	0.538097	-0.484921
C	2.834851	-0.476171	-0.091578
C	2.639660	-0.599212	1.427804
C	1.386025	-0.248674	2.039926
C	3.890162	2.191978	1.550963
C	1.310161	3.341274	-2.666794
C	-2.851115	3.513373	0.157904
C	0.939268	-2.542055	-1.798139
C	-4.049067	4.241703	0.724377
C	-4.818040	-3.154328	-1.217891
C	0.157639	0.248050	1.436387
C	-0.330348	0.253983	3.598442
C	1.104859	-0.269522	3.450760
C	-3.049503	2.185632	-0.499092
C	-4.387506	1.557432	-0.462842
C	-4.746401	0.447308	-1.149750
C	-3.941118	-0.336950	-2.145514
C	-2.988442	-1.389997	-1.539570
C	-3.644951	-2.399735	-0.574178
N	-0.815910	0.550889	2.250273
O	-2.101028	1.590264	-1.048354
O	-2.406054	-2.045120	-2.685694
O	3.610429	-1.019930	2.095748
O	1.799414	-0.607266	4.431880
O	0.023042	0.381685	0.105099
H	-1.843745	5.881136	1.240620
H	-0.332925	5.014083	1.590868
H	-0.527962	5.823189	0.045349
H	-0.813974	3.345914	-0.240029
H	-4.840138	4.355636	-0.026230
H	-4.482002	3.701838	1.576196
H	-3.784487	5.241645	1.073656
H	-0.823466	0.831303	-0.129217
H	-5.138992	2.019517	0.168854
H	-5.762232	0.091394	-0.978543
H	-4.626033	-0.849339	-2.829949
H	-3.314476	0.340255	-2.732434
H	-2.205286	-0.849736	-1.001513
H	-1.562914	-2.440723	-2.418496
H	-5.622144	-2.478001	-1.529493
H	-4.482833	-3.710496	-2.101166
H	-5.248469	-3.870846	-0.508576
H	-4.035021	-1.808509	0.268634
H	-3.167652	-4.117774	0.627062
H	-2.186764	-3.988429	-0.814699
H	-0.874145	-2.102524	0.282268
H	-1.967521	-2.153397	1.643265
H	-1.239333	-4.616709	1.984133
H	-0.062260	-3.367707	2.378034
H	-0.058961	-4.766878	-0.347619
H	0.763240	-5.437492	1.047544
H	2.388854	-3.673844	1.159779
H	1.448998	-2.610438	-2.765822
H	0.127010	-3.275040	-1.797000
H	0.506553	-1.534682	-1.737490
H	3.860066	-2.367120	0.073948
H	3.974992	-2.865674	-2.457302
H	4.677069	-0.851059	-3.610266
H	4.944192	1.186365	-2.281132

H	1.933716	0.706092	-2.510584
H	2.977417	1.374712	-3.756741
H	3.445849	3.355957	-2.399964
H	1.153461	4.356873	-2.280418
H	1.343703	3.397041	-3.762690
H	0.435368	2.739401	-2.386218
H	2.502426	3.718597	-0.176131
H	1.659202	2.172120	-0.222398
H	4.685486	2.518237	-0.388757
H	4.036027	3.255967	1.779131
H	2.976809	1.867236	2.057901
H	4.728843	1.631653	1.979657
H	4.899030	0.145001	-0.098956
H	1.906780	-0.146145	-0.544761
H	-0.344625	1.148165	4.236772
H	-0.962269	-0.502457	4.084862
O	-3.565630	0.286327	2.408744
H	-2.588443	0.402210	2.233680
H	-3.976192	0.281043	1.530076

TS2

G(Water) = -1871.205498 Hartree

C	-6.571820	1.801047	-0.735975
C	1.888971	-4.317815	-0.464334
C	2.460998	-2.946913	-0.849347
C	3.992910	-2.923548	-0.936717
C	4.535198	-1.725240	-1.750258
C	4.176505	-0.366347	-1.197444
C	3.165967	0.438602	-1.575684
C	2.995376	1.813759	-0.927570
C	3.126131	2.898561	-1.975440
C	2.308841	3.951273	-2.080754
C	1.150807	4.177020	-1.146792
C	-5.366906	0.910270	-0.700595
C	-0.185702	3.813681	-1.840165
C	-1.412562	4.020578	-0.937318
C	-1.207633	3.312581	0.412808
C	0.108126	3.733003	1.089613
C	1.340363	3.419790	0.194444
C	1.668091	1.924844	-0.105739
C	1.859473	1.098094	1.170426
C	0.998903	-0.012611	1.487572
C	0.182561	3.241915	2.542489
C	-2.695877	3.552092	-1.632551
C	-5.152458	-0.193993	0.046237
C	2.148834	0.081324	-2.637123
C	-6.154121	-0.757169	1.030665
C	-0.130403	-5.760080	0.046715
C	-0.134620	-0.559934	0.741393
C	-0.062676	-1.842044	2.576232
C	1.072838	-0.818791	2.674296
C	-3.835009	-0.900391	-0.116502
C	-3.484833	-1.932874	0.793697
C	-2.353666	-2.747771	0.646283
C	-1.938450	-3.303467	-0.703927
C	-0.449087	-3.465929	-1.056835
C	0.387319	-4.320740	-0.094644
N	-0.704384	-1.597775	1.296879
O	-3.040569	-0.525329	-1.047733
O	-0.349357	-4.064992	-2.368840
O	2.811744	1.425939	1.915564
O	1.835388	-0.790567	3.665835
O	-0.562192	-0.061175	-0.415999
H	-6.290123	2.826302	-0.456597
H	-6.969798	1.862255	-1.758694
H	-7.381037	1.481920	-0.074076
H	-4.556713	1.205886	-1.363464
H	-6.303478	-1.831698	0.872240
H	-5.815707	-0.631079	2.067701
H	-7.128483	-0.270557	0.946001
H	-1.501854	-0.384104	-0.649502
H	-4.021719	-1.995619	1.734873
H	-2.156056	-3.424225	1.474881
H	-2.402910	-4.299270	-0.749016
H	-2.396482	-2.705555	-1.494689
H	-0.013367	-2.465083	-1.099217
H	-0.699713	-3.435352	-3.019037
H	-1.148399	-5.794430	0.452129
H	-0.132197	-6.273041	-0.921886
H	0.514557	-6.327864	0.728562
H	0.297153	-3.833094	0.878994
H	2.445755	-4.722664	0.393912
H	2.058125	-5.015062	-1.296893
H	2.050386	-2.658527	-1.824093
H	2.128629	-2.178236	-0.139559
H	4.348499	-3.846933	-1.417786
H	4.421595	-2.916187	0.076201
H	4.175027	-1.827582	-2.781972
H	5.630621	-1.804440	-1.796203
H	4.820974	-0.013887	-0.389051
H	2.492786	-0.716928	-3.300142
H	1.213573	-0.254885	-2.173203
H	1.903035	0.955295	-3.252458
H	3.820443	1.946347	-0.214957
H	3.952499	2.777069	-2.677132
H	2.457857	4.679300	-2.878979
H	1.109285	5.251851	-0.909211
H	-0.148773	2.763151	-2.160437
H	-0.291045	4.411349	-2.756770
H	-1.502091	5.100791	-0.735662
H	-3.575371	3.715184	-0.996624
H	-2.857076	4.086719	-2.577721
H	-2.640997	2.478841	-1.856109
H	-2.051733	3.546306	1.078035
H	-1.216232	2.225007	0.262175
H	0.092934	4.834445	1.146925
H	-0.601705	3.729777	3.135784
H	0.032166	2.161567	2.624529
H	1.151035	3.478954	2.996693
H	2.222492	3.842504	0.696268
H	0.856162	1.505852	-0.692740
H	-0.766968	-1.698476	3.407640
H	0.331091	-2.863011	2.654515
O	2.911204	1.543488	4.691881
H	2.992335	1.873435	3.775185
H	2.537641	0.656537	4.483890

IM4

G(Water) = -1871.244265 Hartree

C	6.993703	-0.651491	-0.722382
C	-2.630525	3.976181	-0.419991
C	-3.064293	2.545550	-0.768051
C	-4.574388	2.295699	-0.693035
C	-4.994173	0.995027	-1.419658
C	-4.352007	-0.267015	-0.890332
C	-3.256092	-0.895258	-1.356901
C	-2.789711	-2.206364	-0.720207
C	-2.836383	-3.317462	-1.748323
C	-1.865552	-4.217990	-1.934024
C	-0.606414	-4.237340	-1.110148
C	5.591071	-0.115322	-0.701243
C	0.591186	-3.678976	-1.919106

C	1.907331	-3.676870	-1.123848
C	1.707694	-2.982108	0.234146
C	0.540863	-3.598352	1.024373
C	-0.797581	-3.496724	0.239962
C	-1.386975	-2.079144	-0.041150
C	-1.564188	-1.258661	1.238927
C	-0.796661	-0.056534	1.464696
C	0.515367	-3.113281	2.481199
C	3.045185	-3.035949	-1.927337
C	5.099158	0.930675	-0.000027
C	-2.419466	-0.398756	-2.514962
C	5.933130	1.776772	0.936976
C	-0.716411	5.596619	0.007391
C	0.132970	0.596577	0.535843
C	0.209163	1.842411	2.497818
C	-0.770180	0.687700	2.683411
C	3.660072	1.282094	-0.138756
C	3.050212	2.213588	0.637835
C	1.607456	2.686042	0.548393
C	1.237231	3.238111	-0.856947
C	-0.260912	3.367921	-1.174695
C	-1.115249	4.122152	-0.143815
N	0.634280	1.725784	1.116307
O	3.022135	0.587441	-1.125726
O	-0.426562	4.027536	-2.451698
O	-2.393215	-1.674291	2.076953
O	-1.352778	0.531614	3.781035
O	0.460867	0.247513	-0.629654
H	7.006446	-1.695082	-0.376558
H	7.381283	-0.665305	-1.750860
H	7.697259	-0.086741	-0.105343
H	4.891910	-0.658235	-1.331964
H	5.865085	2.840649	0.678502
H	5.593577	1.676425	1.976147
H	6.987636	1.496439	0.911230
H	2.036237	0.534448	-0.966309
H	3.640181	2.679775	1.417902
H	1.525051	3.535174	1.232914
H	1.715512	4.220911	-0.941745
H	1.679425	2.612133	-1.637553
H	-0.650924	2.352166	-1.262556
H	-0.064456	3.447633	-3.140698
H	0.331996	5.707493	0.309269
H	-0.856042	6.137431	-0.935444
H	-1.335377	6.082166	0.771874
H	-0.933026	3.621280	0.812537
H	-3.169244	4.322280	0.474622
H	-2.924729	4.649630	-1.237019
H	-2.726501	2.317682	-1.785612
H	-2.555034	1.826516	-0.112627
H	-5.115480	3.134835	-1.154967
H	-4.892777	2.260101	0.359250
H	-4.770689	1.119196	-2.487193
H	-6.085446	0.889453	-1.342871
H	-4.845848	-0.704463	-0.019848
H	-2.946454	0.329836	-3.136474
H	-1.503589	0.078022	-2.144218
H	-2.106975	-1.232667	-3.155381
H	-3.505501	-2.459875	0.073559
H	-3.733506	-3.347522	-2.368290
H	-1.964186	-4.972019	-2.715850
H	-0.372746	-5.287672	-0.873582
H	0.361939	-2.652950	-2.238179
H	0.711359	-4.268984	-2.838916
H	2.176457	-4.727128	-0.922648
H	3.993139	-3.083862	-1.375885
H	3.188299	-3.543170	-2.890546
H	2.832366	-1.977775	-2.124845
H	2.632672	-3.061613	0.824057
H	1.524743	-1.911336	0.075018
H	0.735980	-4.682791	1.076246
H	1.408301	-3.478482	3.005034
H	0.514195	-2.022470	2.556457
H	-0.367764	-3.485153	3.012742
H	-1.554632	-4.045608	0.818774
H	-0.713215	-1.549441	-0.710047
H	1.053727	1.736263	3.192709
H	-0.278002	2.804729	2.699524
O	-2.273890	-1.832279	4.873228
H	-2.443080	-2.252894	4.010583
H	-1.949756	-0.957310	4.551644

4-conformer-1

G(Water) = -1795.239137 Hartree

C	-1.549114	3.766135	2.081508
C	3.921369	-1.749686	0.267657
C	2.784989	-1.573636	1.282336
C	2.535865	-2.818959	2.139976
C	1.483159	-2.583966	3.248083
C	0.068358	-2.479684	2.734271
C	-0.627412	-1.374582	2.410940
C	-2.069555	-1.514917	1.925849
C	-3.025012	-0.741028	2.807750
C	-4.082230	-0.062421	2.350357
C	-4.440348	0.020027	0.890428
C	-0.156685	3.440452	1.638879
C	-4.136575	1.436376	0.343163
C	-4.490406	1.593743	-1.142731
C	-3.805126	0.485528	-1.958136
C	-4.157157	-0.915923	-1.430933
C	-3.754611	-1.097319	0.058354
C	-2.236938	-1.117940	0.422339
C	-1.417077	-2.098000	-0.386899
C	-0.359838	-1.641988	-1.270706
C	-3.684357	-2.015651	-2.393517
C	-4.124936	2.989830	-1.659034
C	0.387509	3.578904	0.411382
C	-0.085320	0.032944	2.483920
C	-0.345277	4.129595	-0.790136
C	5.204097	-0.842680	-1.722786
C	0.087048	-0.273612	-1.635105
C	1.383043	-1.819963	-2.875651
C	0.398026	-2.529379	-1.995101
C	1.796853	3.086757	0.240031
C	2.246087	2.758730	-1.136293
C	3.471658	2.318155	-1.493089
C	4.662977	2.039698	-0.619098
C	4.534675	0.729866	0.198388
C	4.171071	-0.521136	-0.631250
N	1.051580	-0.431196	-2.601371
O	2.550613	2.941280	1.214250
O	5.719065	0.538635	0.991653
O	-1.616476	-3.330321	-0.287371
O	0.308450	-3.841822	-1.979923
O	-0.302999	0.810975	-1.197322
H	-2.176065	4.191002	1.294115
H	-2.043898	2.857573	2.451418
H	-1.525416	4.469944	2.925232
H	0.480794	3.006824	2.407616
H	0.278389	4.837981	-1.348549
H	-0.627913	3.321769	-1.474874
H	-1.256973	4.656296	-0.499187
H	3.611581	2.106044	-2.553710

H	5.563973	1.990914	-1.241996
H	4.796364	2.845506	0.108933
H	3.746795	0.900880	0.930534
H	6.477286	0.404093	0.399382
H	5.343508	-0.008541	-2.419512
H	6.182839	-1.094324	-1.292860
H	4.883777	-1.711293	-2.310040
H	3.219930	-0.276614	-1.123452
H	3.691821	-2.610679	-0.377340
H	4.851074	-2.000321	0.796893
H	3.017089	-0.740219	1.957432
H	1.861063	-1.295787	0.757443
H	3.481149	-3.121861	2.613301
H	2.221031	-3.657814	1.501860
H	1.766689	-1.686621	3.813469
H	1.528248	-3.422206	3.956416
H	-0.431911	-3.439572	2.592317
H	0.033730	0.459500	1.479813
H	-0.778607	0.692016	3.021063
H	0.887572	0.085445	2.977384
H	-2.334303	-2.578804	1.997785
H	-2.811481	-0.765817	3.876536
H	-4.718828	0.484515	3.046256
H	-5.526868	-0.134953	0.798056
H	-3.069850	1.657052	0.481722
H	-4.683103	2.177434	0.942918
H	-5.579819	1.461431	-1.246805
H	-4.408143	3.108998	-2.712775
H	-4.631288	3.773688	-1.081045
H	-3.044441	3.160828	-1.579945
H	-4.106773	0.562640	-3.012586
H	-2.716283	0.631948	-1.927491
H	-5.257566	-0.979558	-1.416035
H	-4.250956	-1.951572	-3.330667
H	-2.625712	-1.913176	-2.653291
H	-3.832794	-3.016025	-1.971474
H	-4.165914	-2.062442	0.387297
H	-1.821718	-0.122588	0.261027
H	1.237210	-2.087808	-3.929042
H	2.413871	-2.070075	-2.595132
H	1.507009	2.862269	-1.922065
H	1.616076	0.346281	-2.914267
H	-0.435396	-4.041429	-1.333647

4-conformer-2

G(Water) = -1795.216126 Hartree

C	4.953217	-1.771497	0.496229
C	4.522824	-0.275427	0.294526
C	5.631374	0.574616	-0.407075
C	7.006682	0.114158	0.114227
C	7.361043	-1.360061	-0.237451
C	6.087448	-2.206772	-0.451018
C	3.812210	-2.756105	0.478935
C	2.516943	-2.426406	0.425539
C	2.047551	-0.992712	0.357486
C	3.135331	-0.148919	-0.381857
C	8.316487	-1.480508	-1.433761
C	5.586011	0.634919	-1.948921
C	0.678858	-0.817306	-0.288824
C	-0.277301	-0.154574	0.385665
C	0.531767	-1.334470	-1.701183
C	-1.642547	0.256586	-0.085856
C	-1.810492	1.792908	-0.092301
C	-3.159656	2.254414	-0.668239
C	-4.373463	1.756232	0.125448
C	-5.741880	2.147746	-0.487743
C	-6.217475	3.493092	0.081599
C	-6.759684	0.997861	-0.256855
O	-8.134273	1.436316	-0.272988
H	-8.293836	1.982858	0.512532
C	-6.723945	-0.095443	-1.351925
C	-5.371448	-0.591321	-1.787793
C	-4.600877	-1.561880	-1.252087
C	-4.902751	-2.326314	-0.019939
O	-5.911049	-2.067307	0.654472
C	-3.939512	-3.410102	0.391870
C	-4.341370	-4.231495	1.386436
C	-3.615755	-5.389718	1.996595
C	-2.605690	-3.522161	-0.315251
C	2.643625	1.279739	-0.515903
O	2.424001	1.732085	-1.666278
C	2.339084	2.153841	0.612291
C	2.435888	1.987304	2.090315
N	1.885330	3.114920	2.643183
O	2.899051	1.048765	2.747394
C	1.466525	4.104181	1.668455
H	1.944550	3.300689	3.635157
C	1.776124	3.392081	0.388105
O	1.485937	3.940027	-0.770436
H	-2.626219	-5.570266	1.570322
H	-3.500589	-5.231209	3.078005
H	-4.210511	-6.306872	1.884034
H	-5.327404	-4.035232	1.805756
H	-2.046463	-2.580553	-0.269772
H	-1.975153	-4.292054	0.134039
H	-2.726368	-3.778458	-1.375204
H	-3.666744	-1.780980	-1.756619
H	-4.964645	-0.104420	-2.673895
H	-7.330702	-0.931202	-0.993506
H	-7.218999	0.340944	-2.228749
H	-6.552183	0.534072	0.716596
H	-5.621774	2.273763	-1.574526
H	-5.433002	4.249615	-0.039356
H	-7.116311	3.860027	-0.424726
H	-6.431422	3.415414	1.157016
H	-4.313556	0.669504	0.210756
H	-4.322267	2.139085	1.155891
H	-3.174796	3.352111	-0.720585
H	-3.242347	1.893672	-1.704661
H	-1.690100	2.175786	0.932053
H	-0.996433	2.232056	-0.684461
H	-1.855340	-0.124358	-1.090367
H	-2.396878	-0.184093	0.580311
H	-0.035046	0.184141	1.395511
H	0.956215	-2.341945	-1.793604
H	-0.508822	-1.378724	-2.030918
H	1.077750	-0.694662	-2.407532
H	1.983621	-0.609903	1.382053
H	1.751875	-3.201883	0.454714
H	4.094625	-3.807356	0.551238
H	5.383158	-1.829045	1.510435
H	4.411091	0.143749	1.297373
H	3.206525	-0.524911	-1.407231
H	5.487032	1.609556	-0.061819
H	6.322724	-3.265699	-0.279039
H	5.593869	-0.352125	-2.417964
H	4.696196	1.159081	-2.306062
H	6.463358	1.180837	-2.317543
H	7.788131	0.786344	-0.265063
H	7.006506	0.231196	1.206874
H	7.881387	-1.781542	0.635043
H	8.550726	-2.532801	-1.642403
H	7.877212	-1.049401	-2.340895

H	9.261048	-0.957315	-1.237917
H	2.031589	5.041589	1.747375
H	0.396440	4.336327	1.735804
H	5.743373	-2.140549	-1.490378
H	1.771375	3.252692	-1.452108

2

G(Water) = -1795.24407 Hartree

C	6.504382	-4.084220	-1.752655
C	1.411717	4.315416	-0.793223
C	0.114619	3.571476	-1.150949
C	-0.945739	4.526219	-1.721063
C	-2.096646	3.825825	-2.473048
C	-2.872927	2.832981	-1.645962
C	-2.833095	1.491023	-1.730755
C	-3.749784	0.636924	-0.850482
C	-4.724793	-0.118067	-1.728823
C	-4.947794	-1.434030	-1.661940
C	-4.265231	-2.319488	-0.655142
C	5.901046	-3.097353	-0.803814
C	-3.099802	-3.106607	-1.305552
C	-2.356008	-4.019855	-0.315932
C	-1.953765	-3.238312	0.946954
C	-3.151979	-2.511642	1.581407
C	-3.801605	-1.511842	0.584827
C	-2.935526	-0.305727	0.098552
C	-2.418246	0.523086	1.254885
C	-1.001139	0.600698	1.561138
C	-2.816751	-1.966954	2.978547
C	-1.141854	-4.682434	-0.978274
C	4.638197	-2.618427	-0.773387
C	-1.936280	0.727852	-2.682043
C	3.547628	-3.013042	-1.744792
C	3.567019	4.482006	0.506313
C	0.183136	0.074850	0.836481
C	0.918198	0.974447	2.884111
C	-0.557316	1.149621	2.735572
C	4.314630	-1.617915	0.300315
C	2.907638	-1.038277	0.296176
C	2.703427	0.063410	1.340960
C	3.392417	1.395329	0.965797
C	2.710318	2.167519	-0.190099
C	2.332995	3.610044	0.217086
N	1.277847	0.279367	1.650822
O	5.133371	-1.284561	1.159354
O	3.486980	2.122015	-1.405272
O	-3.218360	1.126451	2.006306
O	-1.282675	1.727628	3.670218
O	0.200293	-0.436709	-0.288109
H	5.809210	-4.457932	-2.507881
H	7.361530	-3.629916	-2.268869
H	6.903616	-4.943002	-1.195368
H	6.580174	-2.718002	-0.041135
H	2.657887	-3.386597	-1.224028
H	3.225224	-2.161362	-2.356743
H	3.878645	-3.797974	-2.427548
H	2.663803	-0.679494	-0.706866
H	2.209807	-1.864157	0.479097
H	3.156826	-0.287803	2.274544
H	3.432869	2.019638	1.865072
H	4.433846	1.176078	0.709695
H	1.793262	1.646272	-0.471416
H	4.348109	2.540731	-1.244091
H	4.245980	4.022444	1.233652
H	4.136376	4.678288	-0.411701
H	3.260053	5.454509	0.908052
H	1.761363	3.507571	1.152805
H	1.151377	5.295637	-0.367110
H	1.972115	4.524923	-1.715176
H	0.326083	2.779663	-1.882265
H	-0.291441	3.063415	-0.265075
H	-0.464310	5.226369	-2.419486
H	-1.351826	5.138909	-0.902784
H	-1.679350	3.344770	-3.365092
H	-2.790313	4.599772	-2.833436
H	-3.535796	3.274898	-0.898116
H	-1.038791	0.374091	-2.161081
H	-2.450247	-0.156854	-3.076096
H	-1.610385	1.333884	-3.531117
H	-4.334855	1.316714	-0.216275
H	-5.243083	0.489219	-2.471304
H	-5.640008	-1.905561	-2.359576
H	-4.998765	-3.059238	-0.299644
H	-2.387261	-2.395994	-1.746225
H	-3.491413	-3.703735	-2.140698
H	-3.054634	-4.813009	-0.003732
H	-0.633141	-5.362304	-0.282855
H	-1.436030	-5.261012	-1.863355
H	-0.414073	-3.924260	-1.298160
H	-1.516781	-3.930678	1.680442
H	-1.166576	-2.512884	0.696129
H	-3.934163	-3.271624	1.739108
H	-2.671485	-2.803968	3.672487
H	-1.892280	-1.382659	2.988293
H	-3.622292	-1.335515	3.370462
H	-4.694277	-1.091429	1.068480
H	-2.073455	-0.693039	-0.441854
H	1.161171	0.377866	3.773278
H	1.422767	1.943857	2.979644
H	-2.223407	1.699125	3.322068

Acetate ion

G(Water) = -228.6926546 Hartree

C	-0.186158	0.000070	-0.010764
O	-0.766232	1.127650	0.002038
O	-0.761411	-1.130141	0.002042
C	1.354156	0.002279	-0.004226
H	1.703469	-0.013097	1.037078
H	1.755417	-0.885979	-0.502733
H	1.754265	0.904908	-0.477054

Acetic acid

G(Water) = -229.1462085 Hartree

C	-0.088642	0.121350	-0.000020
O	-0.635330	1.210886	-0.000007
O	-0.787214	-1.037503	-0.000047
C	1.395780	-0.119437	0.000032
H	1.676077	-0.703814	0.882827
H	1.676417	-0.702309	-0.883668
H	1.926164	0.833196	0.000872
H	-1.741141	-0.825621	0.000331

Imidazole

G(Water) = -226.2565072 Hartree

C	0.638615	-0.988892	0.000000
N	-0.744258	-0.987332	0.000000
C	1.120343	0.299699	-0.000000
C	-1.092413	0.289655	-0.000000
N	-0.000000	1.103239	-0.000000
H	2.120156	0.707362	-0.000000
H	-2.102829	0.674922	-0.000000
H	-0.010231	2.115303	-0.000000

H	1.203441	-1.911710	0.000000

Imidazolium
G(Water) = -226.6937248 Hartree

C	1.140044	0.348199	0.000000
N	-0.000000	1.129262	0.000000
C	0.719804	-0.950315	-0.000000
H	-0.021617	2.143401	0.000000
C	-1.085274	0.351369	-0.000000
N	-0.662541	-0.914921	-0.000000
H	1.269784	-1.877452	-0.000000
H	-2.111343	0.683681	-0.000000
H	-1.275203	-1.723454	-0.000000
H	2.128728	0.777919	0.000000

Imidazolium and Acetate ion complex
G(Water) = -455.3981236 Hartree

C	1.381239	0.968502	-0.000342
N	0.960832	-0.345170	0.000446
C	2.748802	0.960852	-0.000680
H	-0.092423	-0.652078	0.000646
C	2.026157	-1.140598	0.000554
N	3.125379	-0.369496	0.000040
H	3.472962	1.759877	-0.001372
H	2.018889	-2.219413	0.000997
H	4.077603	-0.716483	-0.000017
H	0.661526	1.771675	-0.000586
C	-2.270860	0.053512	-0.000095
O	-1.797211	1.216282	0.001128
O	-1.561074	-1.019722	-0.000658
C	-3.784985	-0.151444	-0.000408
H	-4.080872	-0.732425	-0.881793
H	-4.081324	-0.729873	0.882556
H	-4.315668	0.803952	-0.001773

H_2O
G(Water) = -76.46379981 Hartree

O	0.000000	-0.000000	0.118576
H	-0.000000	0.768475	-0.474304
H	-0.000000	-0.768475	-0.474304

Acetamide
G(Water) = -209.2622659 Hartree

C	-1.364196	-0.343159	-0.000005
H	-1.866023	0.061226	0.885156
H	-1.466525	-1.431603	-0.002080
C	0.076066	0.132508	-0.000113
O	0.358127	1.339487	0.000010
N	1.033917	-0.825568	-0.000171
H	2.009772	-0.555079	0.000891
H	0.816748	-1.812493	0.000221
H	-1.867622	0.064932	-0.882364

Phenol
G(Water) = -307.5046062 Hartree

C	1.861719	0.027595	-0.000000
C	1.133191	1.222037	0.000000
C	1.175284	-1.193085	0.000000
H	1.650782	2.177922	0.000000
C	-0.265190	1.204138	-0.000000
C	-0.221234	-1.224869	-0.000000
H	1.727755	-2.129308	0.000000
H	-0.827810	2.135179	0.000000
C	-0.940266	-0.023262	-0.000000
H	-0.762202	-2.166910	0.000000
O	-2.311142	-0.114035	0.000000
H	-2.708317	0.772833	0.000000
H	2.947903	0.047241	-0.000000

IM1 and acetamide complex
G(Water) = -2080.497671 Hartree

C	-6.384171	4.126606	-1.016006
C	-2.127921	-2.447401	-1.826714
C	-0.860706	-1.584955	-2.032075
C	-1.163150	-0.170531	-2.585576
C	-0.218783	0.929021	-2.078848
C	1.247168	0.661418	-2.346237
C	2.281126	1.354485	-1.841426
C	3.714909	0.868582	-2.045470
C	4.640790	1.998179	-2.428745
C	5.809203	2.254430	-1.831656
C	6.328981	1.432301	-0.682563
C	-5.853339	3.199156	0.031301
C	6.077083	2.165925	0.658818
C	6.573017	1.380046	1.882669
C	6.020463	-0.054518	1.853124
C	6.336280	-0.766586	0.526106
C	5.735297	-0.002171	-0.688119
C	4.185628	0.075663	-0.774077
C	3.527191	-1.310949	-0.849683
C	2.542038	-1.703020	0.127786
C	6.020679	-2.267189	0.606149
C	6.217252	2.100315	3.188714
C	-4.588820	2.755191	0.201454
C	2.106210	2.584511	-0.982891
C	-3.415379	3.139111	-0.668731
C	-3.995300	-3.264952	-0.319665
C	1.939499	-0.862362	1.167392
C	0.917370	-2.967063	1.353102
C	1.941441	-3.000592	0.211289
C	-4.346649	1.826258	1.349773
C	-2.919339	1.515706	1.754259
C	-2.759414	0.180027	2.497109
C	-3.225884	-1.049240	1.707327
C	-2.832036	-1.038634	0.219162
C	-2.694492	-2.450445	-0.387733
N	1.062715	-1.637454	1.918749
O	-5.273118	1.345452	2.016377
O	-3.708706	-0.185618	-0.529659
O	3.867301	-2.057856	-1.792276
O	2.133520	-4.048005	-0.440612
O	2.109239	0.346652	1.422726
H	-5.629459	4.473890	-1.725298
H	-7.185013	3.629631	-1.581274
H	-6.843735	5.005454	-0.542944
H	-6.595942	2.843870	0.744299
H	-2.633722	3.645909	-0.089014
H	-2.966578	2.246573	-1.118393
H	-3.710321	3.812329	-1.476281
H	-3.313523	0.217665	3.442202
H	-2.804165	-1.930320	2.206532
H	-4.314852	-1.125880	1.790527
H	-1.852685	-0.556511	0.137151
H	-4.636041	-0.509505	-0.502435
H	-4.358292	-3.368169	0.709763
H	-4.790332	-2.794413	-0.908837
H	-3.833922	-4.274252	-0.717906
H	-1.951002	-2.958662	0.243715
H	-1.914970	-3.492995	-2.087565
H	-2.912093	-2.115707	-2.521886
H	-0.311499	-1.502737	-1.083610

H	-0.171929	-2.101805	-2.711632
H	-2.183460	0.111112	-2.307306
H	-1.136342	-0.202968	-3.684786
H	-0.375471	1.039707	-0.994720
H	-0.525898	1.890474	-2.518340
H	1.470989	-0.214001	-2.957814
H	2.636911	2.470473	-0.031510
H	2.522220	3.469958	-1.482537
H	1.056512	2.789851	-0.756499
H	3.709899	0.155873	-2.882224
H	4.300681	2.628405	-3.252104
H	6.412774	3.103390	-2.154946
H	7.420205	1.334112	-0.796538
H	4.999699	2.353536	0.765276
H	6.560087	3.153051	0.627333
H	7.671412	1.312360	1.815716
H	6.598643	1.553097	4.060561
H	6.639124	3.113476	3.213904
H	5.127531	2.187382	3.297729
H	6.449331	-0.628256	2.687618
H	4.932134	-0.035431	2.012080
H	7.427633	-0.693640	0.383137
H	6.678448	-2.742593	1.345577
H	4.987328	-2.453699	0.914373
H	6.175319	-2.760955	-0.359572
H	6.086558	-0.505305	-1.600075
H	3.808024	0.590886	0.106169
H	1.135201	-3.758995	2.080895
H	-0.087662	-3.143218	0.952391
H	-2.254395	1.528304	0.890089
H	0.251900	-1.157925	2.306299
H	-2.588733	2.338357	2.408270
O	-1.357146	-0.024979	2.791580
H	-1.024126	0.736413	3.294713
C	-8.660406	-1.615957	-0.628562
H	-8.939166	-1.283386	-1.633610
H	-9.518791	-1.499348	0.038522
C	-7.437782	-0.841511	-0.181441
O	-6.397721	-0.855244	-0.868651
N	-7.524972	-0.156563	0.972940
H	-6.736914	0.401855	1.309750
H	-8.378817	-0.147611	1.513602
H	-8.398212	-2.677330	-0.692156

Transition state of IM1 and acetamide complex
G(Water) = -2080.455875 Hartree

C	-3.484920	-4.544739	-1.914659
C	-0.526409	4.382555	0.943098
C	0.399533	3.810155	-0.141837
C	1.787443	4.472791	-0.178538
C	2.732560	4.017488	0.961683
C	3.317525	2.646379	0.723745
C	2.933685	1.469992	1.249957
C	3.648531	0.179876	0.841911
C	4.047258	-0.627190	2.056108
C	3.891011	-1.950245	2.167224
C	3.284019	-2.797588	1.081493
C	-3.268259	-3.159078	-1.391203
C	1.843443	-3.218316	1.466851
C	1.182432	-4.109266	0.404575
C	1.248248	-3.413960	-0.963935
C	2.690483	-3.042752	-1.350482
C	3.350986	-2.098829	-0.304146
C	2.796235	-0.650730	-0.179070
C	2.837733	0.126914	-1.502109
C	1.640017	0.712245	-2.065463
C	2.774942	-2.596141	-2.817238
C	-0.254948	-4.474797	0.786971
C	-4.132023	-2.119455	-1.362355
C	1.802938	1.309036	2.236173
C	-5.545385	-2.171126	-1.898178
C	-2.835202	4.650148	1.922200
C	0.276464	0.604978	-1.625060
C	0.134397	1.961441	-3.431633
C	1.601900	1.574357	-3.222966
C	-3.635309	-0.837456	-0.768583
C	-4.386422	0.361021	-0.938474
C	-4.104105	1.561120	-0.301298
C	-3.426580	1.677545	1.042740
C	-2.029872	2.336189	1.170126
C	-1.978773	3.853883	0.923380
N	-0.595909	1.260719	-2.382615
O	-2.530304	-0.847221	-0.126199
O	-1.541597	2.087095	2.496886
O	3.947843	0.275169	-2.047216
O	2.504358	1.984346	-3.972295
O	-0.106161	-0.080821	-0.562799
H	-4.496088	-4.728014	-2.286634
H	-2.778002	-4.753870	-2.729966
H	-3.267579	-5.280795	-1.128078
H	-2.276344	-2.973525	-0.984242
H	-6.260187	-1.770791	-1.169776
H	-5.650969	-1.573924	-2.813479
H	-5.852484	-3.191332	-2.138970
H	-1.104221	-0.207321	-0.486418
H	-5.171621	0.367009	-1.688280
H	-4.743016	2.398492	-0.562816
H	-4.117325	2.226292	1.696085
H	-3.317717	0.675501	1.460724
H	-1.374497	1.848008	0.445647
H	-3.893476	4.366890	1.880794
H	-2.485127	4.498234	2.950120
H	-2.777076	5.723277	1.701036
H	-2.380672	3.999605	-0.084753
H	-0.564257	5.475274	0.816480
H	-0.099764	4.202629	1.938415
H	0.533667	2.732277	-0.004049
H	-0.089456	3.933441	-1.116341
H	1.668935	5.565614	-0.135134
H	2.272512	4.250827	-1.139862
H	2.202710	4.065086	1.919766
H	3.560748	4.736649	1.034071
H	4.130267	2.625628	-0.005846
H	2.184435	1.049606	3.233652
H	1.183305	2.201837	2.330578
H	1.138707	0.490927	1.942298
H	4.572362	0.463597	0.319345
H	4.493158	-0.062162	2.875749
H	4.190113	-2.458193	3.084919
H	3.875491	-3.723990	1.004253
H	1.229952	-2.318767	1.616906
H	1.867790	-3.739946	2.434408
H	1.768893	-5.040302	0.334867
H	-0.708472	-5.137766	0.038880
H	-0.294375	-4.982080	1.759789
H	-0.873844	-3.573851	0.857631
H	0.823623	-4.075237	-1.733309
H	0.623451	-2.509658	-0.946141
H	3.280240	-3.972381	-1.282744
H	2.510235	-3.436684	-3.471781
H	2.082636	-1.777580	-3.038741
H	3.783844	-2.259450	-3.078867
H	4.411284	-2.006059	-0.579195

H	1.774785	-0.700771	0.186726
H	-0.205291	1.662691	-4.430754
H	0.004782	3.046188	-3.337946
O	-2.709900	2.521575	-1.777282
H	-1.586399	1.734093	-2.036903
H	-3.314950	2.273748	-2.495981
C	-1.029267	-2.560087	4.460822
H	-0.056124	-3.016678	4.244411
H	-1.758732	-3.358593	4.622716
C	-1.387752	-1.648456	3.305554
O	-0.813408	-0.552648	3.162493
N	-2.328710	-2.082950	2.444718
H	-2.447778	-1.611009	1.541405
H	-2.704910	-3.017364	2.531103
H	-0.926114	-1.966292	5.373008
H	-1.304700	1.139191	2.583457

IM1 and phenol complex
G(Water) = -2178.741462 Hartree

C	-7.720800	0.066713	-3.714360
C	-3.290699	-2.325010	2.535891
C	-2.196397	-2.653271	1.493602
C	-2.767847	-3.048409	0.110732
C	-1.878161	-2.668589	-1.083468
C	-0.449742	-3.154620	-0.970779
C	0.600275	-2.696587	-1.672769
C	2.020999	-3.133677	-1.318341
C	2.836965	-3.448592	-2.549264
C	4.056181	-2.955973	-2.792753
C	4.759600	-2.019219	-1.846259
C	-7.001831	0.841860	-2.654354
C	4.667023	-0.559096	-2.358864
C	5.354766	0.455921	-1.431457
C	4.854959	0.279929	0.012058
C	5.019953	-1.169742	0.500389
C	4.229074	-2.166549	-0.394385
C	2.678790	-2.042779	-0.405822
C	2.063898	-2.180876	0.994293
C	1.335872	-1.070351	1.567597
C	4.793839	-1.285332	2.014912
C	5.157774	1.892138	-1.929703
C	-5.721971	0.719499	-2.242467
C	0.474059	-1.682996	-2.784856
C	-4.726676	-0.270548	-2.801774
C	-4.741069	-0.621895	3.722663
C	0.854984	0.102534	0.861171
C	0.291460	0.422014	3.126732
C	0.987677	-0.934202	2.950949
C	-5.265830	1.656012	-1.159961
C	-3.771296	1.796525	-0.920673
C	-3.397513	2.347062	0.461645
C	-3.865538	1.488788	1.644011
C	-3.736903	-0.030641	1.438759
C	-3.565843	-0.818652	2.752658
N	0.357720	1.040110	1.802416
O	-6.055239	2.345480	-0.510095
O	-4.834034	-0.524602	0.641351
O	2.236843	-3.251735	1.607622
O	1.184626	-1.681930	3.927807
O	0.828515	0.369465	-0.350300
H	-7.107200	-0.696675	-4.198532
H	-8.604606	-0.424729	-3.284006
H	-8.096882	0.748246	-4.489940
H	-7.599710	1.605369	-2.157710
H	-3.871608	0.234588	-3.268327
H	-4.332593	-0.912063	-2.005191
H	-5.176437	-0.916423	-3.558799
H	-3.811113	3.355143	0.580259
H	-3.283024	1.802201	2.518681
H	-4.911384	1.734618	1.857878
H	-2.857222	-0.225445	0.818741
H	-5.669132	-0.290795	1.077846
H	-4.898872	0.433276	3.972534
H	-5.675223	-1.016213	3.301281
H	-4.556925	-1.161713	4.658994
H	-2.667494	-0.394412	3.223585
H	-3.015185	-2.740749	3.514190
H	-4.228332	-2.821784	2.249682
H	-1.513848	-1.799441	1.385661
H	-1.564688	-3.467297	1.869162
H	-3.738712	-2.561648	-0.028849
H	-2.958935	-4.131537	0.102433
H	-1.882119	-1.572522	-1.174803
H	-2.348619	-3.040725	-2.007027
H	-0.257739	-3.902303	-0.199767
H	1.223381	-0.891964	-2.680827
H	0.640733	-2.159852	-3.760895
H	-0.510950	-1.209568	-2.809129
H	1.952342	-4.053850	-0.720876
H	2.368302	-4.120747	-3.269631
H	4.572473	-3.212243	-3.718649
H	5.827770	-2.287698	-1.831180
H	3.608807	-0.284252	-2.468379
H	5.104973	-0.502105	-3.365586
H	6.434769	0.234035	-1.434987
H	5.683832	2.609909	-1.287083
H	5.534803	2.014671	-2.953561
H	4.095043	2.164835	-1.923903
H	5.410814	0.956911	0.676817
H	3.798850	0.579503	0.078770
H	6.076853	-1.437167	0.333227
H	5.584529	-0.739746	2.546213
H	3.835819	-0.856893	2.322356
H	4.816202	-2.329829	2.345589
H	4.472504	-3.181841	-0.049803
H	2.411304	-1.066614	-0.803995
H	0.812428	1.017983	3.885421
H	-0.741618	0.276019	3.460454
H	-3.257126	0.847599	-1.080468
H	-0.473849	1.536313	1.471949
H	-3.393351	2.478717	-1.699091
O	-1.955775	2.426314	0.563223
H	-1.606966	2.944102	-0.181188
C	3.104561	5.379526	-1.308207
C	2.163111	4.347710	-1.391597
C	3.808296	5.563752	-0.111168
H	1.617705	4.182221	-2.318006
C	1.918008	3.508538	-0.300779
C	3.569364	4.737089	0.989208
H	4.547707	6.357082	-0.030527
H	1.217742	2.682019	-0.383690
C	2.620174	3.707627	0.899113
H	4.109101	4.875615	1.922329
O	2.424881	2.928937	2.002853
H	1.676736	2.284735	1.861681
H	3.292077	6.025169	-2.161874

Transition state of IM1 and phenol complex
G(Water) = -2178.697251 Hartree

C	5.259799	-0.143789	-1.894329
C	-3.777411	3.188846	0.257109
C	-3.307863	2.083920	1.219495

C	-4.450802	1.427616	2.012851
C	-5.324215	0.453358	1.181922
C	-4.640407	-0.867513	0.922586
C	-3.946680	-1.256679	-0.161907
C	-3.276055	-2.631532	-0.188650
C	-3.637760	-3.393315	-1.444030
C	-2.763501	-4.076429	-2.190185
C	-1.298351	-4.163789	-1.855690
C	3.942302	0.554156	-1.763506
C	-0.469716	-3.249570	-2.792749
C	1.038622	-3.290493	-2.498947
C	1.297518	-3.029370	-1.005805
C	0.493907	-3.984326	-0.106245
C	-1.034563	-3.861350	-0.356848
C	-1.727329	-2.518331	0.016530
C	-1.507887	-2.112017	1.478441
C	-0.876943	-0.848733	1.822798
C	0.942389	-3.901741	1.360235
C	1.804735	-2.302818	-3.386848
C	3.696672	1.864155	-1.546095
C	-3.736401	-0.393096	-1.382941
C	4.779924	2.903041	-1.370562
C	-3.246487	5.167783	-1.224646
C	-0.302603	0.136937	0.969170
C	-0.003356	1.006073	3.053343
C	-0.710947	-0.345843	3.171577
C	2.262560	2.280027	-1.410804
C	1.956579	3.595602	-0.968022
C	0.674698	4.144281	-0.896336
C	-0.454099	3.755611	-1.831482
C	-1.724692	3.102321	-1.269893
C	-2.649206	4.000894	-0.423545
N	0.262409	1.176478	1.621947
O	1.352416	1.424887	-1.693038
O	-2.402103	2.598354	-2.449157
O	-1.916742	-2.883894	2.361298
O	-1.033816	-0.827983	4.265827
O	-0.286985	0.083598	-0.341227
H	6.115785	0.532389	-1.968233
H	5.419007	-0.794719	-1.023593
H	5.258262	-0.797244	-2.777434
H	3.072132	-0.094752	-1.824932
H	4.590921	3.793151	-1.982086
H	4.840236	3.232621	-0.324872
H	5.763762	2.515007	-1.644676
H	0.350649	0.757834	-0.792822
H	2.763387	4.185765	-0.546198
H	0.631119	5.174094	-0.555351
H	-0.742226	4.665697	-2.373802
H	-0.063717	3.048041	-2.565069
H	-1.414290	2.250946	-0.661941
H	-3.213695	2.148703	-2.168572
H	-2.470543	5.809374	-1.657831
H	-3.877914	4.805437	-2.044922
H	-3.866954	5.796662	-0.574512
H	-2.011394	4.400408	0.370870
H	-4.400598	3.895823	0.823714
H	-4.449475	2.768487	-0.507550
H	-2.768018	1.299622	0.677843
H	-2.582576	2.521398	1.917295
H	-5.093199	2.212221	2.438406
H	-4.028986	0.874930	2.864008
H	-5.629603	0.939232	0.247827
H	-6.250027	0.257944	1.739832
H	-4.684372	-1.567148	1.759710
H	-3.966617	-0.940914	-2.304412
H	-4.364259	0.501451	-1.360448
H	-2.688727	-0.076422	-1.457269
H	-3.663775	-3.202201	0.666257
H	-4.690164	-3.362537	-1.729319
H	-3.100614	-4.585645	-3.093620
H	-0.968227	-5.198220	-2.040674
H	-0.826458	-2.214825	-2.693368
H	-0.655611	-3.539901	-3.836435
H	1.396882	-4.308344	-2.724467
H	2.884032	-2.349631	-3.192743
H	1.642643	-2.515543	-4.451499
H	1.475682	-1.273641	-3.193367
H	2.370330	-3.142471	-0.796930
H	1.041647	-1.988418	-0.767687
H	0.738847	-5.007140	-0.437670
H	1.969385	-4.275392	1.453742
H	0.930554	-2.876943	1.743417
H	0.298985	-4.505394	2.009578
H	-1.524858	-4.646713	0.235832
H	-1.347621	-1.740415	-0.639222
H	0.921608	1.010093	3.641219
H	-0.648692	1.811565	3.422682
O	-0.003452	3.636612	1.030279
H	0.152846	2.254404	1.244573
H	0.789048	3.998485	1.461630
H	2.147372	1.366194	1.511109
C	5.627191	-1.711633	1.630743
C	6.117405	-0.423278	1.879672
C	4.260116	-1.885322	1.390493
H	7.178441	-0.269682	2.060832
C	5.255453	0.674467	1.893455
C	3.387248	-0.794333	1.401148
H	3.865129	-2.876357	1.187407
H	5.626664	1.678697	2.078420
C	3.885274	0.491772	1.657895
H	2.329346	-0.937064	1.202739
O	3.090777	1.598010	1.686841
H	6.300178	-2.564399	1.617990

Supplementary References

[1] Bierman, M. et al. *Gene* **116,** 43–49 (1992).

[2] Li, L. et al. *Appl. Microbiol. Biot.* **101,** 5291–5300 (2017).

[3] Green M. R. & Sambrook J. Molecular cloning: a laboratory manual 4th ed. *Cold Spring Harbor Laboratory Press*, **2012**.

[4] Igarashi, Y. et al. *Org. Lett.* **12,** 3402–3405 (2012).

[5] Zhu, H. J. et al. *J. Am. Chem. Soc.* **143,** 4751–4757 (2021).

[6] Gui, C. et al. *Org. Lett.* **17**, 628–631 (2015).

[7] Kelley, L. A., Mezulis, S., Yates, C. M., Wass, M. N. & Sternberg, M. J. E. *Nat. Protoc.* **10**, 845–858 (2015).

[8] Zheng, J., Taylor, C. A., Piasecki, S. K., Keatinge-Clay, A. T. *Structure* **18**, 913–922 (2010).
[9] Zheng, J., Piasecki, S. K., Keatinge-Clay, A. T. *ACS Chem. Biol.* **8**, 1964–1971 (2013).
[10] Zheng, J., Gay, D. C., Demeler, B., White, M. A., Keatinge-Clay, A. T. *Nat. Chem. Biol.* **8**, 615–621 (2012).
[11] Keatinge-Clay, A. T., Stroud, R. M. *Structure* **14**, 737–748 (2006).
[12] Holm, L. *Protein Sci.* **29**, 128–140 (2020).
[13] Yu, J. et al. *Bioinformatics* **26**, 46–52 (2010).
